# Low-Cost, Safe, and Anion-Flexible Method for the Electrosynthesis of Diaryliodonium Salts

**DOI:** 10.1021/acs.joc.4c01521

**Published:** 2024-09-20

**Authors:** Anton Scherkus, Aija Gudkova, Jan Čada, Bernd H. Müller, Tomas Bystron, Robert Francke

**Affiliations:** † Leibniz Institute for Catalysis, Albert-Einstein-Str. 29a, 18059 Rostock, Germany; ‡ Department of Inorganic Technology, University of Chemistry and Technology, Prague, Technicka 5, 16628 Prague 6, Czech Republic

## Abstract

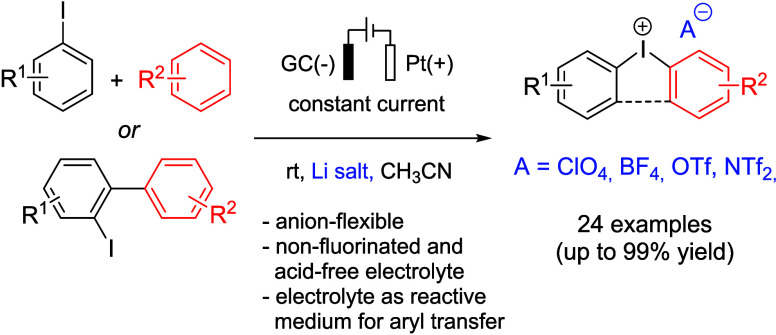

An electrochemical approach toward the synthesis of diaryliodonium salts based on anodic C–I coupling between aryl iodides and arenes is presented. In contrast to previous protocols, our method requires no chemical oxidants, strong acids, or fluorinated solvents. A further advantage is that by use of the appropriate supporting electrolyte, the counterion of choice can be introduced, which is time- and cost-saving as compared to postsynthesis ion exchange. This “anion-flexibility” is particularly interesting when considering the pronounced effect of the counterion on the reactivity of diaryliodonium species in aryl transfer reactions. The scope of our method comprises 24 examples with isolated yields of up to 99%. Scalability was demonstrated by the synthesis on a gram scale. Furthermore, it was shown that the diaryliodonium-containing post-electrolysis solution can be used without further workup as a reactive medium for *O*-arylation reactions. Finally, a series of *para*-substituted diaryliodonium compounds was studied using linear sweep voltammetry on a microelectrode and analyzed with respect to the influence of the electronic structure on the redox behavior.

## 1 Introduction

**Figure 1:**
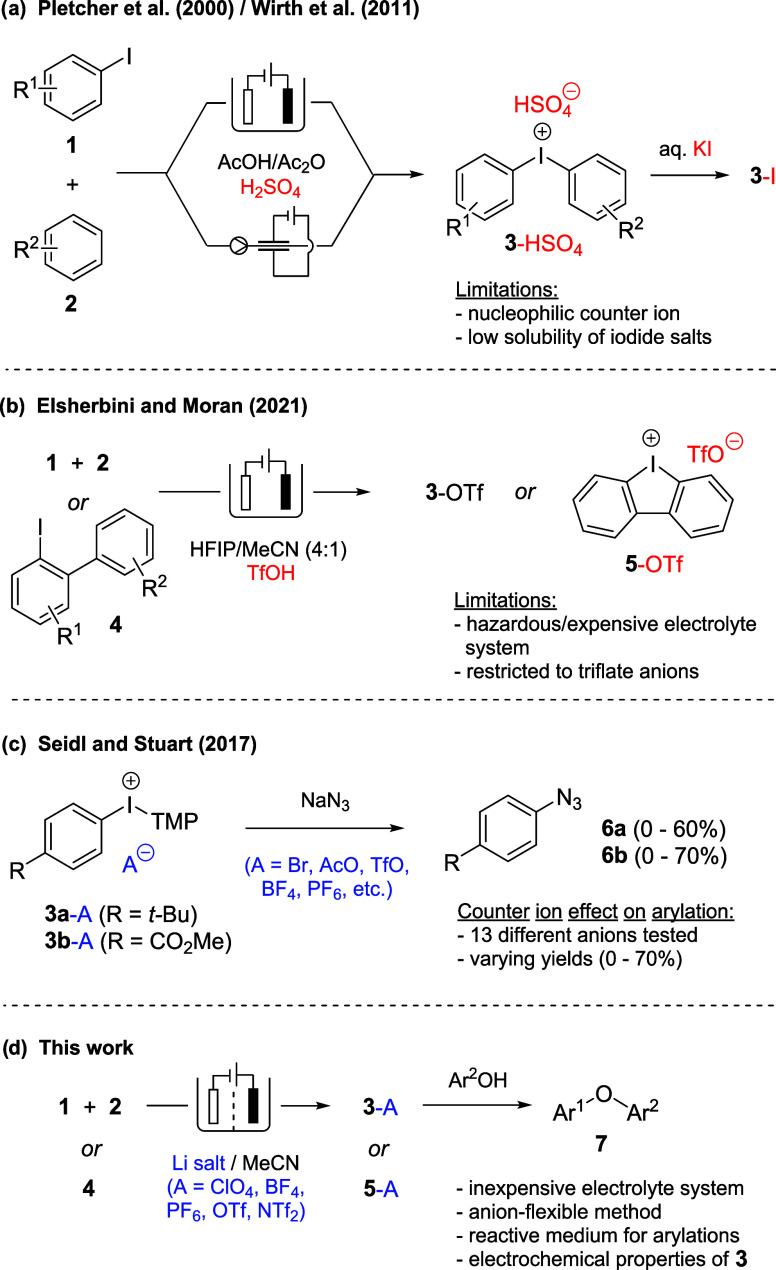
Electrochemical synthesis of diaryliodonium compounds: state of the art and present work.

Diaryliodonium salts have received a growing interest over the last decades as metal-free, easy-to-handle, and highly selective arylation reagents.^1−3^ Since many existing nonelectrochemical procedures are waste-intensive, time-consuming, and involve reagents with a certain toxicity,^4−7^ electrochemical approaches have been developed for the synthesis of hypervalent iodine compounds to harness the major advantages of electric current as traceless and cheap oxidant.^8−11^ Pletcher et al. reported a method that is based on anodic oxidation of an aryl iodide (**1**) in the presence of excess arene (**2**) in an electrolyte consisting of acetic acid, acetic anhydride, and sulfuric acid (5%). Under galvanostatic conditions, a number of diaryliodonium salts **3** were synthesized in up to 92% isolated yield (Figure 1a).^12,13^

Using the same electrolyte components, a protocol for a microflow reactor was later developed by Wirth et al. (Figure 1a).^14^ According to the authors, it is, in principle, possible to isolate the bisulfate salts of **3**, albeit in poor yields. Improved results were achieved by exchanging the anion with iodide after completing electrolysis.^12^ However, the utility of **3-I** salts in organic synthesis is limited due to their low solubility in many organic solvents and the nucleophilicity of the anions.^15^

Recently, another electrochemical protocol has been developed by Elsherbini and Moran (Figure 1b), wherein acyclic and cyclic diaryliodonium triflates (**3**-OTf and **5**-OTf) are synthesized in the presence of triflic acid from **1** and **2** and *via* intramolecular cyclization of 2-iodobiaryls (**4**). The method is straightforward (galvanostatic conditions, undivided cell) and readily scalable but requires the expensive 1,1,1,3,3,3-hexafluoroisopropanol (HFIP) as a solvent.^15^

The electrochemical methods for the synthesis of **3** and **5** have in common that they only provide access to the diaryliodonium species with a specific counterion. However, the nature of the counterion can have a strong influence on the success of the arylation reactions.^16−21^ This is particularly well illustrated through a systematic study by Stuart et al., in which the influence of a total of 13 different counterions on the formation of aryl azides **6** from **3** is investigated (Figure 1c).^22^ Depending on the anionic species, the yields vary between 0 and 70%, emphasizing the importance of selecting the appropriate counterion. Therefore, the development of an anion-flexible method for the preparation of **3** and **5** is desirable, which is preferentially free of hazardous and costly chemicals. In this context, we present an approach based on the anodic oxidation of **1** (or **4**) in acetonitrile in the presence of Li salts, enabling the introduction of different counterions and avoiding the addition of acids and fluorinated additives (Figure 1d). The present study is based on a brief report by Miller and Hoffmann, which describes the feasibility in three examples using a LiClO_4_–CH_3_CN electrolyte under potentiostatic conditions in a divided cell.^23^ Our work includes an optimization of the method, an investigation of the substrate scope, and the introduction of different counterions. Furthermore, the possibility of using the postelectrolysis solution as a reactive medium for one-pot arylations is explored. Finally, compounds **3** are analyzed with regard to their electrochemical properties.

## 2 Results and Discussion

In terms of practicability and scalability, the approach presented by Hoffmann and Miller has the disadvantage of potentiostatic reaction control.^23^ We therefore first addressed the question of whether electrolysis could also work in the much more practical galvanostatic mode. To this end, reactions were carried out in an H-type divided cell under ambient conditions (glassy carbon cathode and platinum anode), applying different current densities and charge equivalents (for details, see Figure S1). The optimum current density was identified as 10 mA cm^–2^. Since the conversion of iodobenzene and benzene to **3a** is two-electron oxidation, theoretically, 2 F per mole of iodoarene would be needed for full conversion. However, an excess of charge (4 F) is necessary to achieve a 93% yield under the standard conditions (Table 1, entry 1). Inferior results were obtained by increasing the current density (entry 2) and by decreasing the number of charge equivalents (entry 3).

**Table 1:**
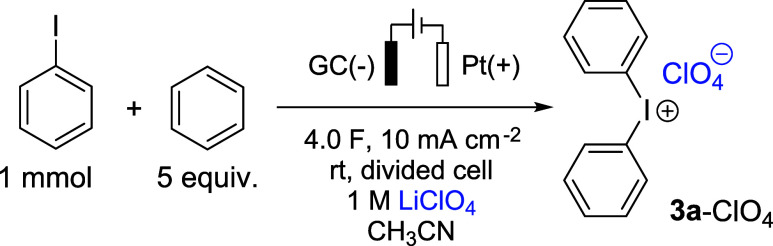
Optimization of the Reaction Conditions

entry	deviation from standard conditions	yield [%]^a^
1	none	93
2	15 mA cm^–2^	85
3	3.0 F	75
4	quasi-divided cell	10
5	0.5 M LiClO_4_	–b
6	graphite anode	84
7	glassy carbon anode	83
8	2 equiv benzene	72

a Yields determined by ^1^H NMR spectroscopy using mesitylene as the internal standard.

b Electrolysis could not be finished due to increasing cell resistance.

With the intention of further increasing practicability, an experiment was carried out in an undivided cell that was equipped with a platinum sheet anode of standard size and a stainless steel wire cathode, a so-called quasi-divided cell,^24^ which resulted in a very low yield (entry 4). Similarly, reducing the supporting electrolyte concentration to 0.5 M did not produce a useful result; the electrolysis had to be interrupted due to a sharp increase in the cell voltage (entry 5). Further tests showed that the platinum electrode can be substituted by graphite or glassy carbon, although with a slight reduction in yield to 84 and 83%, respectively. Moreover, the impact of reducing the amount of benzene equivalents on the yield was checked (Table 1, entry 8). Using two equiv of benzene, **3a** is furnished in 72% yield, whereby formation of the homocoupling product (**3b**) occurred as a side reaction (20% yield).

Aiming at an improved understanding of the reaction at hand, the product mixture was subjected to additional tests. A qualitative pH determination showed that the anolyte becomes more acidic during electrolysis, which can be ascribed to the release of a proton in the anodic coupling process. On the other hand, a precipitate is formed in the cathode compartment, which was identified as LiOH *via* powder X-ray diffraction analysis of the dried solid (Figure S3). Both residual water and atmospheric O_2_ may serve as precursors for hydroxide formation, whereby the exact mechanism of formation is presently unclear.

**Figure 2:**
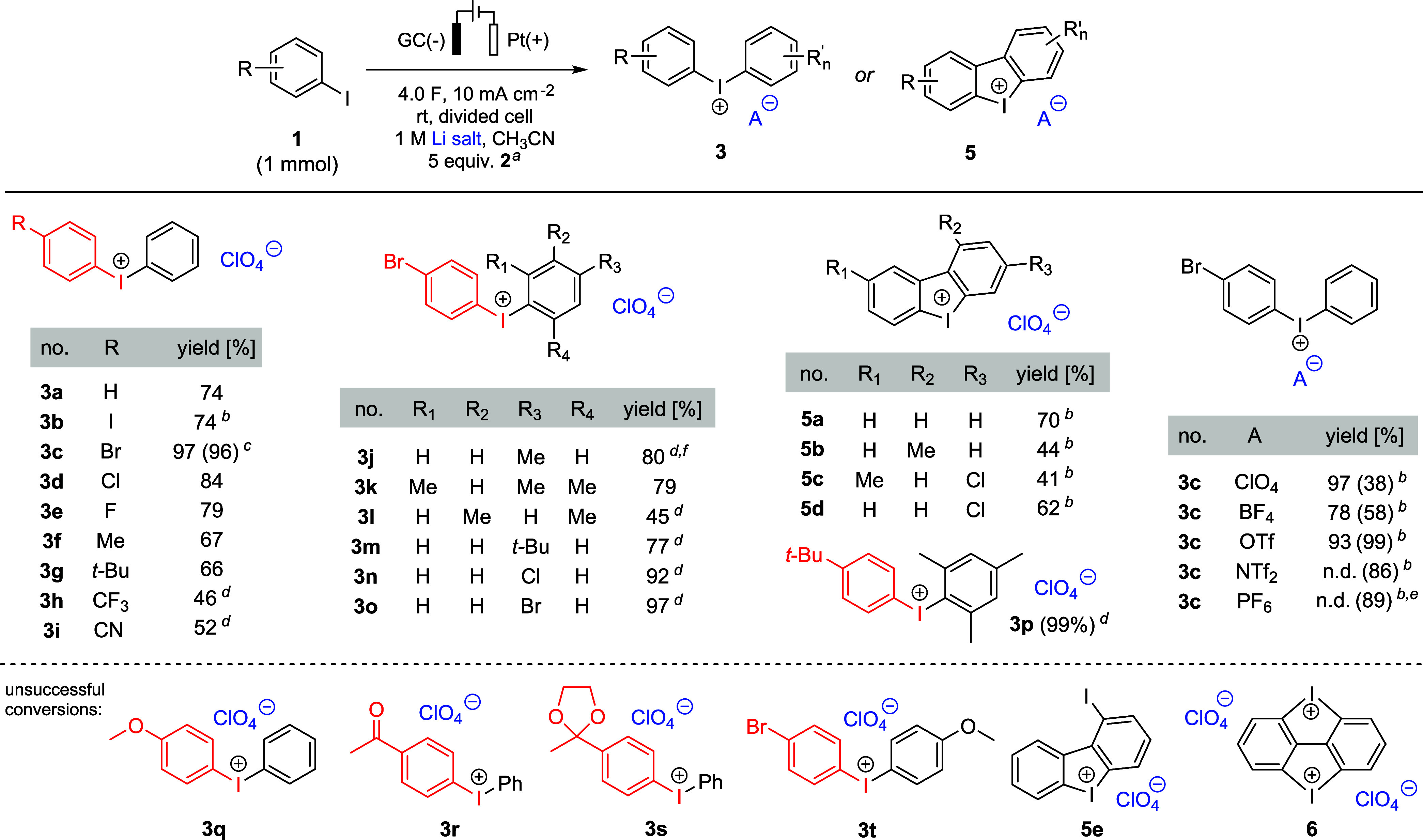
Scope of the electrochemical synthesis of diaryliodonium salts. ^a^ Addition of **2** occurred only when intermolecular coupling was intended. ^b^ 2.0 F per mole of iodoarene applied. ^c^ Batch size: 7 mmol of **1**. ^d^ Merely 2 equiv of arene **2** were used. ^e^ Isolation of 449 mg of a resinous diaryliodonium salt that contains different counterions derived from hydrolysis of PF_6_^–^ (yield calculated assuming 100% hexafluorophosphate content). ^f^ The *ortho*-tolyl isomer could be observed in a quantity of 0.08 mmol, which corresponds to a molar ratio of 1:9 of *ortho*-tolyl isomer to the desired compound **3j** (see the Supporting Information (SI) for further details).

Applying the optimized conditions, the scope of our method was investigated by oxidizing different aryl iodide derivatives to diaryliodonium salts in the presence of LiClO_4_ and various arene compounds (Figure 2). For the isolation of **3**-ClO_4_, the anolyte was concentrated under reduced pressure, followed by column chromatography. In several cases, the product was then obtained as an oil, which had to be further purified by crystallization from CH_2_Cl_2_/pentane to obtain a crystalline solid. In the case of **3a**-ClO_4_, *e.g.*, repeated purification was necessary to remove small amounts of homocoupling product **3b**. This led to a diminished yield of 74%, which is significantly smaller than the spectroscopically determined yield (93%, compare Table 1). On the other hand, the homocoupling product can be synthesized by oxidizing iodobenzene in the absence of a second arene, rendering **3b**-ClO_4_ in 74% yield. Here, as in several other cases, unidentified side products occurred upon passing 4 F mol^–1^ of iodoarene, which is why the number of charge equivalents was reduced to 2 F mol^–1^ (footnote b in Figure 2).

Further investigations involved the conversion of a series of *para*-substituted iodoarenes with benzene (**3c**–**i** as perchlorate salts). In general, iodoarenes with slightly electron-withdrawing or -donating substituents (R = Br, Cl, F, Me, *t*-Bu) give better yields (66–97%) than strongly electron-withdrawing ones (R = CF_3_, CN with 46 and 52%, respectively). To demonstrate the scalability of the protocol, synthesis of **3c**-ClO_4_ was repeated using a 7 mmol batch, furnishing 3.09 g of the product (96% yield; for details, see Figure S2). A strongly electron-donating *para* group such as OMe, on the other hand, leads to a complex mixture of various products, from which it was not possible to isolate the desired product **3q**. Furthermore, 4-iodoacetophenone as well as its acetal protected derivative are unsuitable starting materials since the formation of **3r** and **3s** could not be observed.

In the next step, arene component **2** was varied in the presence of 4-bromoiodobenzene. Toluene, mesitylene, 4-xylene, and *tert*-butyl benzene proved to be suitable coupling partners, rendering the corresponding diaryliodonium salts **3j**–**m** in 45–80% yield. Excellent results were obtained when using chloro- and bromobenzene, giving **3n** and **3o** in 92 and 97% yield, respectively. Another outstanding combination consists of 4-*tert*-butyliodobenzene and mesitylene, which affords **3p** in an almost quantitative yield. In contrast, the reaction of 4-bromoiodobenzene with anisole does not lead to the formation of the desired product **3t** but instead to a complicated reaction mixture with unidentified products.

For the synthesis of cyclic iodonium species **5a**–**d**, the corresponding 2-iodobiphenyls were electrolyzed under optimized conditions. Oxidative cyclization was feasible in all four cases, whereby the yields varied between 41 and 70%. A limitation is represented by 2,2′-diiodobiphenyl. In an attempt to synthesize **5e** and bisiodonium species **6**, respectively, one-fold deiodination led to the formation of **5a**, which was isolated in 64% yield. A similar case, where oxidation of 2,2′-diiodobiphenyl with peracetic acid led to the formation of **5a**, has been reported previously.^25^

Preparation of diaryliodonium salts with a specific counterion is often connected to additional resource-intensive anion exchange steps.^22,26^ Given the strong influence of the anion on the reactivity of **3**, an electrochemical method that provides access to diaryliodonium salts with the desired counterion without additional anion exchange steps would be highly attractive. With this in mind, we carried out electrolysis under optimized conditions with different Li salts as supporting electrolytes and isolated the resulting products (Figure 2, right). For comparison, yields were determined after passing both 4.0 and 2.0 F per mole 4-bromoiodobenzene. In the cases of **3c**-ClO_4_ and **3c**-BF_4_, better results were obtained when applying 4.0 F (97 and 78% yield). However, in the cases of **3c**-OTf, **3c**-NTf_2_, and **3c**-PF_6_, the formation of byproducts was observed when passing excess charge, and reducing the charge equivalents to 2.0 F rendered superior results. Under these circumstances, the products were isolated in 99, 86, and 89% yield, respectively. It should be noted that the hexafluorophosphate anion turned out to undergo partial hydrolysis during the reaction, which is why **3c**-PF_6_ contains a mixture of different counterions (mostly PO_3_F^2–^ and PO_2_F_2_^–^; for details, see the SI). The sensitivity of the PF_6_^–^ ion to residual water in the presence of acids is well known and has been reported elsewhere.^27−29^

**Scheme 1:**
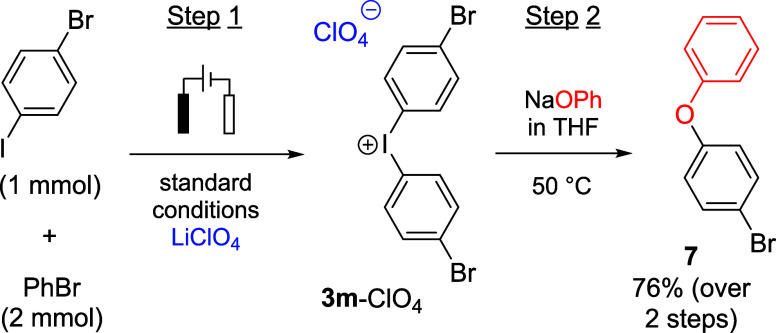
*O*-Arylation of Phenol in a Two-Step One-Pot Protocol

To demonstrate the utility of our electrochemical method with respect to metal-free arylation reactions, a two-step, one-pot method for the preparation of diaryl ether **7** was developed (Scheme 1; for details on optimization, see the SI). To minimize the synthetic effort, the electrolyte solution was to be used as the reactive medium without further workup. Since the reaction of electrochemically generated **3m**-ClO_4_ with phenolate in pure acetonitrile proceeds rather slowly, the anolyte was mixed with a NaOPh solution in tetrahydrofuran (THF). Under these conditions, **7** could be isolated in a 76% yield with respect to the iodoarene precursor.

To explore the electrochemical behavior of **3**, a series of derivatives bearing one 4-substituted and one unsubstituted phenyl ring was analyzed by linear sweep voltammetry (LSV) using a Pt microelectrode in 0.1 M LiClO_4_–CH_3_CN (for details, see the SI). Half-wave potentials *E*_1/2_ (**3**) were approximated by the potential *E*_1/2_′ (**3**) at which inflection of the first wave/peak in voltammogram occurs (determined *via* the maximum of the first derivative of the current, d*I* d*E*^–1^). In most cases, the measured currents significantly decreased from cycle to cycle. This can be ascribed either to reductive grafting onto the electrode surface^30,31^ or to blocking of the electrode surface byproducts of radical recombination/oligomerization.^32^ Therefore, the very first potential sweep after diaryliodonium salt addition was always used for analysis.

**Figure 3:**
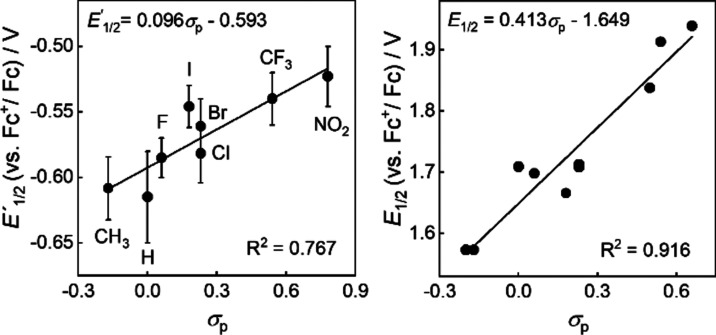
Plot of half-wave potentials of (4-*R*-phenyl)(phenyl)iodonium compounds (left) and 4-substituted iodoarenes (right) vs the corresponding *σ*_p_ parameters.

As can be seen in Figure 3 (left), a scattered but obvious linear dependence between *E*_1/2_′ (**3**) values and Hammett substituent constants (σ_p_)^33^ was found, showing clear electronic effects of the para substituents on the redox behavior. However, it is noticeable that the experimental values deviate significantly from the redox potentials predicted using quantum chemical methods. For diphenyl-substituted **3a**-ClO_4_, *e.g.*, *E*_1/2_′ (**3**) is positively shifted by approximately 0.4 V compared to the computed value.^34^ Furthermore, the slope of the linear fit (0.1 V) is somewhat smaller than the one obtained from predicted values.^35^ A possible explanation is that under electrochemical conditions, the formed species are different from the products assumed in the modeling studies. In the latter case, dissociative single electron reduction (concerted electron transfer) is assumed to render Ar_(solvated)_^1•^ and Ar^2^I_(solvated)_, which was also supported by low-temperature electron spin resonance (ESR) studies using ionizing irradiation.^36^ In this respect and considering the above-mentioned grafting abilities of diaryliodonium salts,^30,31^ it appears likely that the observed shift of reduction potentials to less negative values is due to attachment of the arene radicals to the electrode surface leading to their stabilization. More specifically, the products of electrochemical reduction of **3**-ClO_4_ are not fully solvated Ar^1•^ and Ar^2^I but Ar^1^I in solution and only partially solvated surface-bound aryl moiety Ar^1^.

For comparison, the oxidation potentials *E*_1/2_(ArI) of relevant iodoarenes were measured and correlated with the respective σ_p_ values. In this case, a clear correlation was found (Figure 3, right). The fact that the slope of the linear fit (0.41 V) is more than four times higher than observed for diaryliodonium salt reduction, suggesting that the influence of the electronic effect of the para substituent is much more pronounced. This stronger impact is not unexpected, since the diaryliodonium species bear a second aryl moiety (in our case, Ph), which can provide or accommodate electron density, thus buffering the effect of the first aryl group with investigated substituent R.

## 3 Conclusions

In conclusion, a straightforward method for the electrosynthesis of diaryliodonium salts in a nonfluorinated medium was developed. The scope comprises a broad structural variety, *i.e.*, acyclic diaryliodonium salts **3** derived from electron-rich and electron-poor iodoarene/arene precursors, as well as cyclic diaryliodonium species **5**. In contrast to previous protocols, our method does not require the addition of strong acids. Under these conditions, the desired anion can be introduced by choice of the appropriate supporting electrolyte, which is more resource-efficient compared to postsynthesis counterion exchange. Key to success is the use of a divided cell, which prevents diaryliodonium ions from being reduced at the cathode.

The scalability of the approach was demonstrated by synthesis on a gram scale. Furthermore, the possibility of using the electrolyte solution as a reactive medium was successfully demonstrated, allowing for the use of *in situ*-generated diaryliodonium species for *O*-arylations in a two-step one-pot procedure. Finally, the electrochemical behavior of a series of diaryliodonium salts was investigated. A clear influence of the electronic substituent effect on the reducibility was observed, whereby the impact of the 4-substituent on the redox potential is less pronounced for **3** than for iodoarenes **1**. Further studies with respect to the anion effect on electrochemical behavior and reactivity of **3** are ongoing in our laboratory.

## 4 Experimental Section

### 4.1 General Electrolysis Procedure

Electrolyses were carried out at room temperature in an H-type divided glass cell (Figure S2, left) in which the half-cells were separated by a G4 glass frit. A Rohde & Schwarz HMP 4040 galvanostat or a Wenking LPG03 potentiostat-galvanostat was used as the power source. The anolyte solution was prepared by dissolving iodoarene **1** (1.0 mmol, 1.0 equiv, 0.2 M), arene **2** (5.0 mmol, 5.0 equiv, 1.0 M), and the respective lithium salt (5.0 mmol, 5.0 equiv, 1.0 M) in acetonitrile (5 mL). The catholyte solution consisted of the respective lithium salt (5.0 mmol, 5.0 equiv, 1.0 M) in acetonitrile (5 mL). After complete dissolution of all compounds, both solutions were carefully added to their respective half-cell compartment *via* syringes at the same time. A glassy carbon plate (thickness: 3 mm, width: 10 mm, immersion depth: 1 cm, SIGRADUR G, HTW GmbH, Germany) was used as the cathode, and a platinum sheet was used as the anode (width: 10 mm, immersion depth: 1 cm). The distance between both electrodes was 3.5 cm. Reactions were carried out under atmospheric conditions at room temperature at *j* = 10 mA cm^–2^, applying four charge equivalents per mole iodoarene (*Q* = 4.0 F, unless stated otherwise).

Upon complete electrolysis, the anolyte was concentrated under reduced pressure, and the residue was subjected to column chromatography for purification using a CH_2_Cl_2_/MeOH (97:3) eluent mixture unless noted otherwise. In several cases, an oily residue was obtained after chromatography instead of a solid product. In these cases, the oil was taken up in a small amount of CH_2_Cl_2_, layered with pentane, and stored at −40 °C overnight to induce the crystallization of the product.

### 4.2 Gram-Scale Electrosynthesis of **3c**

The anolyte was prepared by dissolving *p*-bromoiodobenzene (1.98 g, 7.0 mmol, 1.0 equiv, 0.2 M), benzene (2.73 g, 35.0 mmol, 5.0 equiv, 1.0 M), and lithium perchlorate (3.72 g, 35.0 mmol, 5.0 equiv, 1.0 M) in acetonitrile (35 mL). The catholyte solution consisted of lithium perchlorate (3.72 g, 35 mmol, 5.0 equiv) in acetonitrile (35 mL). After complete dissolution of all compounds, both solutions were carefully added to their respective half-cell compartment *via* syringes at the same time. Electrolysis was carried out at room temperature in a larger H-type divided batch glass cell equipped with a G4 frit as a separator (see Figure S2, right) at *j* = 10 mA cm^–2^ applying four charge equivalents per mole of iodoarene (*Q* = 4.0 F). A glassy carbon plate (thickness: 3 mm, width: 10 mm, SIGRADUR G, HTW GmbH, Germany) was used as the cathode and a platinum sheet (width: 10 mm) as the anode. Both electrodes were immersed 3 cm into the solutions. The interelectrode distance was 6 cm.

### 4.3 Linear Sweep Voltammetry

The experiments were performed in a glass cell of 4 mL volume using an Autolab PGSTAT128N instrument (Metrohm). A 0.1 M solution of LiClO_4_ in CH_3_CN (LiChrosolv Reag. Ph Eur.) was used as the electrolyte. A platinum wire served as the counter electrode, and an Ag/AgNO_3_ electrode (Ag wire in 0.1 M AgNO_3_/CH_3_CN) was the reference. A platinum microelectrode of 25 μm diameter was used as the working electrode. For the analysis of each diaryliodonium salt (**3**) and iodoarene (**1**), respectively, a fresh 2 mL solution of blank electrolyte was prepared, and the working electrode was polished prior to the measurements. For this purpose, a polishing cloth was used with an aqueous suspension of 0.03 μm SiO_2_ particles (Sensolytics). Before the analyte (∼2 mM for iodoarenes and ∼0.5 mM for diaryliodonium salts) was added to the electrolyte solution, a series of scans were performed until a stable current response was achieved. Details regarding evaluation of the LSVs are provided in the SI.

## Data Availability

The data underlying this study are available in the published article and its Supporting Information.

## Supporting Information Available

Procedures for preparation of starting materials, compound characterization data, copies of NMR spectra, powder XRD data from analysis of the product of the cathodic half-cell reaction, and analysis of the counterions in **3c**-PF_6_ (PDF)
