# Supplementary material for: Low-Cost, Safe, and Anion-Flexible Method for the Electrosynthesis of Diaryliodonium Salts
Source: J Org Chem. 2024 Sep 20;89(19):14129–34. doi: 10.1021/acs.joc.4c01521 (PMC11460726; doi:10.1021/acs.joc.4c01521)
Supplement: Supplementary file 1 — jo4c01521_si_001.pdf [file jo4c01521_si_001.pdf]

Supporting Information

A Low-cost, Safe, and Anion-flexible Method for the Electrosynthesis of Diaryliodonium Salts

Anton Scherkus,[1] Aija Gudkova,[1] Jan Čada,[2] Bernd H. Müller,[1] Tomas Bystron,[2] Robert Francke [1,*]

[1] Leibniz Institute for Catalysis, Albert-Einstein-Str. 29a, 18059 Rostock, Germany.

[2] Department of Inorganic Technology, University of Chemistry and Technology Prague, Technicka 5, 16628, Prague 6, Czech Republic.

* Corresponding author. E-mail: robert.francke@catalysis.de.

Content

1. General remarks 2

2. Synthesis of starting materials 3

   2.1 2-(4-Iodophenyl)-2-methyl-1,3-dioxolane 3

   2.2 2-Aminobiphenyls 3

   2.3 2-Iodobiphenyls 4

3. Preparative scale electrolysis 6

   3.1 Optimization of the reaction conditions 6

   3.2 General procedure for the electrosynthesis of diaryliodonium salts 6

   3.3 Aryl transfer reactions 8

   3.4 Characterization of diaryl iodonium salts 9

4. NMR spectra 15

5. Powder XRD patterns 53

6. Analysis of the counter ions in **3c**-PF_6 54

7. Determination of redox potentials 56

1. General remarks

^{1}H and ^{13}C NMR spectra were recorded using an AVANCE 300 or 400 spectrometer (Bruker). Chemical shifts (δ) are reported in parts per million (ppm) with the residual solvent peak as an internal reference. High resolution mass spectrometry (HRMS) was carried out with time-of-flight electrospray ionization (ESI-TOF) using a UPLC H-Class/XEVO G2-XS (Waters Acquity). All starting materials were purchased from BLDpharm, Carbolution, Sigma-Aldrich, TCI, or Alfa Aesar and used without further purification. Acetonitrile in HPLC grade was purchased from Fisher Scientific and used as solvent for electrolysis without purification.

2. Synthesis of starting materials

2.1 2-(4-Iodophenyl)-2-methyl-1,3-dioxolane

4-Iodoacetophenone (1.23 g, 5.0 mmol), ethylene glycol (0.56 mL, 10.0 mmol) and *p*-toluenesulfonic acid monohydrate (0.047 g, 0.25 mmol) were dissolved in 30 mL of toluene. Triethyl orthoformate (1.25 mL, 7.5 mmol) was added and the mixture was boiled under reflux conditions for two hours using an oil bath as heat source. The solution was then concentrated under reduced pressure and taken up in 10 mL of ethyl acetate. The organic phase was extracted with water (3 x 10 mL). Upon removal of the organic solvent the product was obtained as orange solid (1.26 g, 87%).

^{1}H NMR (300 MHz, CD_2Cl_2) δ 7.68 (d, *J* = 8.6 Hz, 2H), 7.23 (d, *J* = 8.5 Hz, 2H), 4.06 – 3.93 (m, 2H), 3.79 – 3.67 (m, 2H), 1.59 (s, 3H). ^{13}C{^{1}H} NMR (75 MHz, CD_2Cl_2): δ 143.8, 137.6, 127.8, 108.7, 93.7, 64.9, 27.6. Spectral data are in agreement with the literature.[1]

2.2 2-Aminobiphenyls

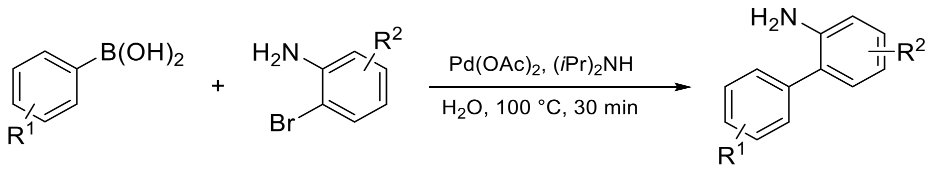

A1 **A2** **A3**

The 2-aminobiaryl precursors **A3** required for synthesis of 2-iodobiaryls **4** were synthesized by Suzuki-Miyaura coupling between *o*-bromoanilines and the corresponding arylboronic acids according to a procedure reported in the literature.[2]

2-Amino-2'-methyl-1,1'-biphenyl (A3b)

A mixture of *o*-tolylboronic acid (2.04 g, 15.0 mmol), 2-bromoaniline (2.58 g, 15.0 mmol), $Pd(OAc)_2$ (8.40 mg, 37 µmol/0.25 mol%), and diisopropylamine (3.04 g, 30 mmol) in 30 mL H_2O was stirred for 30 minutes at 100 °C using an oil bath as heat source. The reaction mixture was then added to 45 mL of brine and extracted with ethyl acetate (3 x 60 mL). The combined organic layers were dried over anhydrous $MgSO_4$ and then subjected to flash column chromatography for further purification (9:1 *n*-heptane/ethyl acetate) to eventually obtain the desired product as colourless oil (1.49 g, 54%).

^{1}H NMR (300 MHz, $CDCl_3$) δ 7.25 – 7.05 (m, 5H), 6.98 (dd, *J* = 7.5, 1.2 Hz, 1H), 6.76 – 6.65 (m, 2H), 3.39 (br s, 2H), 2.09 (s, 3H). ^{13}C{^{1}H} NMR (75 MHz, $CDCl_3$) δ 143.7, 138.7, 137.1, 130.4, 130.2, 130.1, 128.4, 127.8, 127.5, 126.2, 118.3, 115.1, 19.8. Spectral data are in agreement with the literature.[3]

---

[1] Murakami, M.; Ishida, N.; Masuda, Y. Synthesis of Acylphosphonates by a Palladium-Catalyzed Phosphonocarbonylation Reaction of Aryl Iodides with Phosphites. *Chem. Asian J.* **2014**, *10*, 321-324.
[2] Elsherbini, M.; Moran, W. J. Scalable electrochemical synthesis of diaryliodonium salts. *Org. Biomol. Chem.* **2021**, *19*, 4706-4711.
[3] Chen, Y.-Z.; Ding, T.-H.; Li, Q.-Q.; Qu, J.-P.; Kang, Y.-B. Ambient Temperature Dehydrogenative C(Ar)–H Carbonylative Lactamization of 2-Arylanilines Using DMF as C1-Source. *Org. Lett.* **2023**, *25*, 2611-2615.

2-Amino-4'-chloro-5-methyl-1,1'-biphenyl (A3c)

A mixture of (4-chlorophenyl)boronic acid (2.35 g, 15.0 mmol), 2-bromo-4-methylaniline (2.79 g, 15.0 mmol), $Pd(OAc)_2$ (8.40 mg, 37 µmol/0.25 mol%) and diisopropylamine (3.04 g, 30 mmol) in 30 mL of H_2O was stirred for 30 minutes at 100 °C using an oil bath as heat source. The reaction mixture was added to 45 mL of brine and extracted with ethyl acetate (3 x 60 mL). The combined organic layers were dried over anhydrous $MgSO_4$ and the solvent was removed under reduced pressure to obtain the product as light brown oil (2.95 g, 90%).

^1H NMR (300 MHz, $CDCl_3$) δ 7.56 – 7.43 (m, 4H), 7.08 – 7.04 (m, 1H), 7.01 – 6.98 (m, 1H), 6.75 (d, J = 8.0 Hz, 1H), 3.67 (br s, 2H), 2.36 (s, 3H). $^{13}C\{^1H\}$ NMR (75 MHz, $CDCl_3$) δ 140.9, 138.2, 133.0, 130.9, 130.5, 129.4, 129.0, 128.1, 126.5, 116.0, 20.5. Spectral data are in agreement with the literature.[4]

2.3 2-Iodobiphenyls

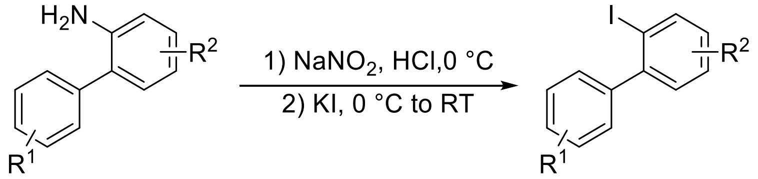

A3 **4**

2-Iodobiaryl compounds **4** were prepared from **A3** using the Sandmeyer reaction according to a procedure reported in the literature.[2]

2-Iodo-2'-methyl-1,1'-biphenyl (4b)

2-Amino-2'-methyl-1,1'-biphenyl (**A3a**, 1.49 g, 8.12 mmol) was dissolved in 20 mL of diluted hydrochloric acid (4 mL conc. HCl in 16 mL H_2O) and stirred for one hour at 0 °C. Afterwards, an aqueous solution of $NaNO_2$ (0.84 g, 12.18 mmol in 11 mL of H_2O) was added dropwise and the mixture was stirred for another hour at 0 °C. Next, KI (2.70 g, 16.2 mmol in 16 mL H_2O) was added and the mixture was allowed to warm to room temperature and stirred overnight. The pH was adjusted to 7 by addition of $NaHCO_3$. The product was extracted from the reaction mixture with ethyl acetate (3 x 27 mL) and the combined organic layers were then dried over $MgSO_4$. After filtration, the solvent was removed under reduced pressure to yield the crude product, which was purified by flash column chromatography using 100% *n*-heptane as eluent. The product was obtained as colorless oil (1.24 g, 52%).

^1H NMR (300 MHz, $CDCl_3$) δ 7.84 (ddd, J = 7.9, 1.3, 0.4 Hz, 1H), 7.28 (td, J = 7.5, 1.2 Hz, 1H), 7.23 – 7.09 (m, 4H), 6.99 – 6.89 (m, 2H), 1.98 (s, 3H). $^{13}C\{^1H\}$ NMR (75 MHz, $CDCl_3$) δ 146.9, 144.5, 139.0, 135.7, 130.0, 129.8, 129.3, 128.8, 128.2, 128.1, 125.7, 100.2, 20.1. Spectral data is in agreement with the literature.[5]

4'-Chloro-2-iodo-5-methyl-1,1'-biphenyl (4c)

2-Amino-4'-chloro-5-methyl-1,1' (**A3b**, 2.94 g, 13.5 mmol) was dissolved in 17 mL of diluted hydrochloric acid (3.5 mL conc. HCl in 13.5 mL H_2O) and stirred for one hour at 0 °C. Afterwards, an aqueous solution of $NaNO_2$ (1.4 g, 20.25 mmol in 9 mL of H_2O) was added

[4] Chatterjee, T.; Lee, D. S.; Cho, E. J. Extended Study of Visible-Light-Induced Photocatalytic [4 + 2] Benzannulation: Synthesis of Polycyclic (Hetero)Aromatics. *J. Org. Chem.* **2017**, *82*, 4369-4378.

[5] Pan, S.; Jiang, H.; Zhang, Y.; Chen, D.; Zhang, Y. Synthesis of Triphenylenes Starting from 2-Iodobiphenyls and Iodobenzenes via Palladium-Catalyzed Dual C–H Activation and Double C–C Bond Formation. **2016**, *18*, 5192-5195.

dropwise and the mixture was stirred for another hour at 0 °C. Next, KI (4.48 g, 27.0 mmol in 13.5 mL H_2O) was added and the mixture was allowed to warm to room temperature and stirred overnight. The pH was adjusted to 7 by addition of $NaHCO_3$. The product was extracted from the reaction mixture with ethyl acetate (3 x 22.5 mL) and the combined organic layers were then dried over $MgSO_4$. After filtration, the solvent was removed under reduced pressure to yield the crude product, which was purified by flash column chromatography using 100% *n*-heptane as eluent. The product was obtained as a light orange solid (2.11 g, 48%).

^{1}H NMR (300 MHz, $CDCl_3$) δ 7.81 (d, *J* = 8.1 Hz, 1H), 7.39 (d, *J* = 8.8 Hz, 2H), 7.27 (d, *J* = 8.7 Hz, 2H), 7.10 (d, *J* = 1.2 Hz, 1H), 6.95 – 6.81 (m, 1H), 2.33 (s, 3H). ^{13}C{^{1}H} NMR (75 MHz, $CDCl_3$) δ 145.3, 142.7, 139.5, 138.4, 133.8, 131.1, 130.8, 130.2, 129.2, 128.3, 94.3, 21.0. Spectral data are in agreement with the literature.[6]

4'-Chloro-2-iodo-1,1'-biphenyl (4d)

2-Amino-4-chlorobiphenyl (611 mg, 3.0 mmol) in 2 mL of H_2O was added to 5.5 mL of diluted hydrochloric acid (1.5 mL conc. HCl in 4 mL H_2O) at 0 °C. After one hour of stirring, $NaNO_2$ (310 mg, 4.5 mmol) in 4 mL H_2O was added dropwise and the mixture was stirred for another hour. Afterwards, KI (996 mg, 6.0 mmol) in 6 mL of water was added. The mixture was allowed to warm to room temperature, stirred overnight, and then treated with $NaHCO_3$ to adjust the pH to 7. The brown solution was extracted with ethyl acetate (3 x 10 mL) and the combined organic layers were then dried over $MgSO_4$. After filtration the solvent was removed under reduced pressure to yield the desired product as brown oil (0.49 g, 52%).

^{1}H NMR (300 MHz, $CDCl_3$) δ 7.9 (dd, *J* = 7.9, 1.7 Hz, 1H), 7.3 (d, *J* = 8.7 Hz, 3H), 7.2 (d, *J* = 8.7 Hz, 3H), 7.0 (ddd, *J* = 7.9, 7.3, 1.8 Hz, 1H). ^{13}C{^{1}H} NMR (75 MHz, $CDCl_3$) δ 145.4, 142.5, 139.6, 133.8, 130.7, 130.0, 129.1, 128.2, 128.2, 98.4. Spectral data are in agreement with the literature.[7]

2,2'-Diiodobiphenyl (4e)

Compound **4e** was synthesized according to a literature-known procedure.[8] At -78 °C, *n*BuLi (6.42 mmol, 2.5 M in hexanes) was added to a solution of 2,2'-dibromobiphenyl (1.00 g, 3.21 mmol) in THF (40 mL). After one hour, a solution of iodine (1.63 g, 6.42 mmol) was added, and the solution was allowed to reach room temperature. After addition of saturated aqueous solution of sodium thiosulfate (30 mL), the organic layer was separated, and the aqueous layer was extracted with ethyl acetate (3 x 30 mL). The combined organic layers were dried over sodium sulfate before being evaporated. The obtained brown material was further purified using flash column chromatography using 100% cyclohexane as eluent. The product was obtained as colorless solid (0.98 g, 75%).

^{1}H NMR (400 MHz, $CDCl_3$) δ 7.95 (dd, *J* = 8.0, 1.2 Hz, 2H), 7.43 (td, *J* = 7.5, 1.2 Hz, 2H), 7.20 (dd, *J* = 7.6, 1.7 Hz, 2H), 7.10 (ddd, *J* = 8.0, 7.4, 1.7 Hz, 2H). ^{13}C{^{1}H} NMR (101 MHz, $CDCl_3$) δ 149.1, 139.0, 130.0, 129.5, 128.2, 99.8. Spectral data are in agreement with the literature.[9]

---

[6] Möckel, R.; Hille, J.; Winterling, E.; Weidemüller, S.; Faber, T. M.; Hilt, G. Electrochemical Synthesis of Aryl Iodides by Anodic Iododesilylation. *Angew. Chem. Int. Ed.*, **2018**, *57*, 442-445

[7] Iwasaki, M.; Iino, S.; Nishihara, Y. Palladium-Catalyzed Annulation of *o*-Iodobiphenyls with o-Bromobenzyl Alcohols: Synthesis of Functionalized Triphenylenes via C–C and C–H Bond Cleavages, *Org. Lett.*, **2013**, *15*, 5326-5329.

[8] Bonnafoux, L.; Gramage-Doria, R.; Colobert, F.; Leroux, F. R. Catalytic Palladium Phosphination: Modular Synthesis of C1-Symmetric Biaryl-Based Diphosphines, *Chem. Eur. J.*, **2011**, *17*, 11008 – 11016.

[9] Xu, Y; Sun, M.; Xu, W; Deng, G.; Liang Y.; Yang, Y. Versatile Tetrasilane for Time-Controlled Palladium-Catalyzed Divergent Synthesis of Silacycles via C–H Activation. *J. Am. Chem. Soc.*, **2023**, *145*, 15303-15312.

3. Preparative scale electrolysis

3.1 Optimization of the reaction conditions

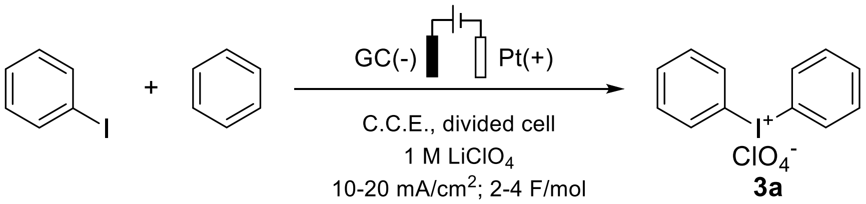

3a

Optimization of current density (*j*) and charge (*Q*) was performed for synthesis of **3a**-ClO_4 (Figure S1) using the electrolysis set up described in section 3.2. Upon completed electrolysis, mesitylene was added to the anolyte as internal standard and the yield was quantified *via* ^{1}H NMR spectroscopy.

j [mA cm^{-2}]	*Q* [F]	NMR Yield [%]	*FE* [%]
10	2	59	59
	3	75	52
	4	93	49
15	2	67	67
	3	85	61
	4	83	44
20	2	64	64
	3	79	56
	4	77	41

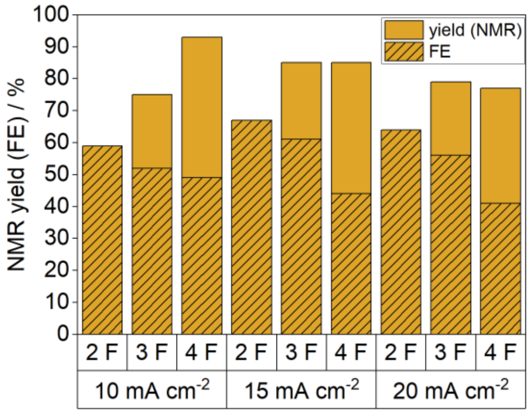

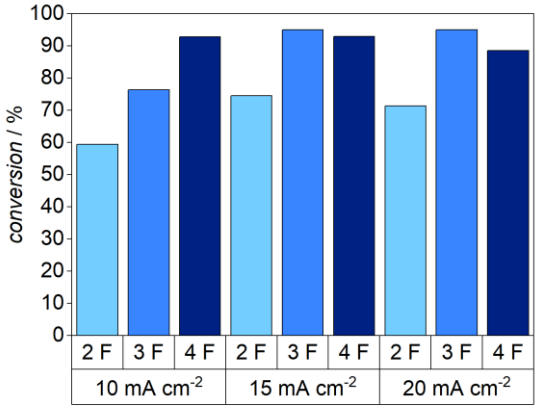

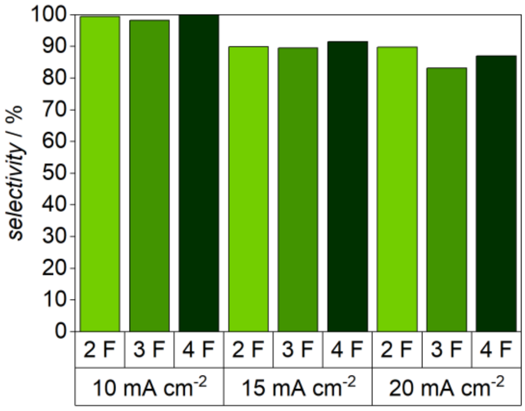

Figure S1. Top left: Results of the screening of charge equivalents and current density. Top right and bottom: Plots of yield, Faraday efficiency (*FE*), conversion and selectivity for product **3a**-ClO_4.

3.2 General procedure for the electrosynthesis of diaryliodonium salts

Electrolyses were carried out at room temperature in an H-type divided glass cell (see Figure S2, left), in which the half-cells were separated by a G4 glass frit. A Rohde & Schwarz HMP 4040 galvanostat or a Wenking LPG03 potentiostat-galvanostat was used as the power source. The anolyte solution was prepared by dissolving iodoarene **1** (1.0 mmol, 1.0 equiv., 0.2 M), arene coupling partner **2** (5.0 mmol, 5.0 equiv., 1.0 M), and the respective lithium salt (5.0 mmol, 5.0 equiv., 1.0 M) in acetonitrile (5 mL). The catholyte solution consisted of the

respective lithium salt (5.0 mmol, 5.0 equiv., 1.0 M) in acetonitrile (5 mL). After complete dissolution of all compounds, both solutions were carefully added to their respective half-cell compartment *via* syringes at the same time. A glassy carbon plate (thickness: 3 mm, width: 10 mm, immersion depth: 1 cm, SIGRADUR G, HTW GmbH, Germany) was used as the cathode and a platinum sheet as the anode (width: 10 mm, immersion depth: 1 cm). The distance between both electrodes was 3.5 cm. Reactions were carried out under atmospheric conditions at room temperature at j = 10 mA cm^{-2} applying four charge equivalents per mole iodoarene (Q = 4.0 F, unless stated otherwise).
Upon completed electrolysis, the anolyte was concentrated under reduced pressure, and the residue subjected to column chromatography for purification using a CH_2Cl_2/MeOH (97:3) eluent mixture, unless noted otherwise. In several cases, an oily residue was obtained after chromatography instead of a solid product. In these cases, the oil was taken up in a small amount of CH_2Cl_2, layered with pentane, and stored at −40 °C overnight to induce crystallization of the product.

For gram-scale electrosynthesis of **3c**, the anolyte was prepared by dissolving *p*-bromoiodobenzene (1.98 g, 7.0 mmol, 1.0 equiv., 0.2 M), benzene (2.73 g, 35.0 mmol, 5.0 equiv., 1.0 M), and lithium perchlorate (3.72 g, 35.0 mmol, 5.0 equiv., 1.0 M) in acetonitrile (35 mL). The catholyte solution consisted of lithium perchlorate (3.72 g, 35 mmol, 5.0 equiv.) in acetonitrile (35 mL). After complete dissolution of all compounds, both solutions were carefully added to their respective half-cell compartment *via* syringes at the same time. Electrolysis was carried out at room temperature in a larger H-type divided batch glass cell equipped with a G4 frit as separator (see Figure S2, right) at j = 10 mA cm^{-2} applying four charge equivalents per mole iodoarene (Q = 4.0 F). A glassy carbon plate (thickness: 3 mm, width: 10 mm, SIGRADUR G, HTW GmbH, Germany) was used as the cathode and a platinum sheet (width: 10 mm) as the anode. Both electrodes were immersed 3 cm into the solutions. The interelectrode distance was 6 cm.

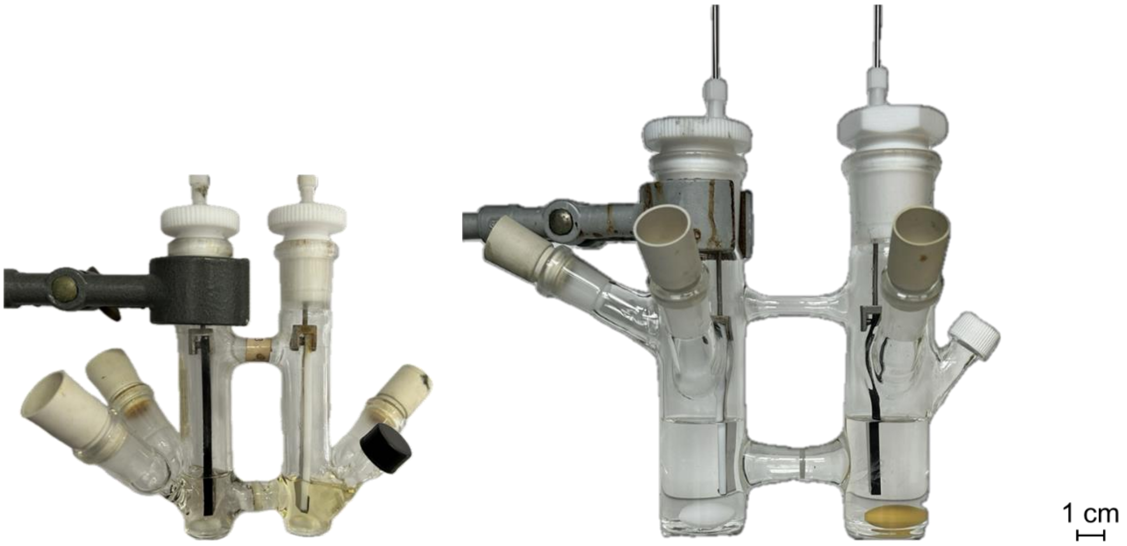

Figure S2: H-type divided cell used for the electrosynthesis of diaryliodonium salts **3** and **5** (anode: Pt sheet folded around a Teflon plate, cathode: glassy carbon plate). Left: cell used for 1 mmol scale experiments. Right: cell used for upscaling experiment.

3.3 Aryl transfer reactions

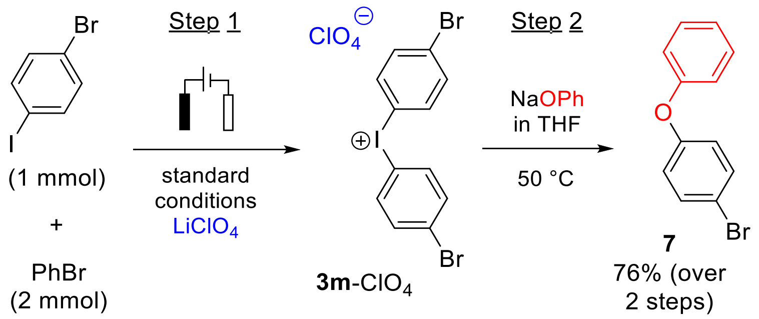

Step 1: The diaryliodonium species **3m**-ClO_4 was prepared by electrolysis of 4-bromo-1-iodobenzene under standard conditions according to section 3.2 in the presence of 2 equiv. PhBr. After completed electrolysis, the anolyte was transferred to a volumetric flask and the volume adjusted to 10 mL by adding CH_3CN. An aliquot (1 mL) was taken from the solution and mixed with internal standard. The aliquot was then subjected to ^1H NMR spectroscopy for quantification of the yield of **3m**-ClO_4. The arylation reaction was carried out using 8 mL of the diluted anolyte solution, which was added to a sodium phenolate solution, followed by stirring at the respective temperature (*cf.* Table S1) for 6 h. After cooling to room temperature, silica gel was added, and the solvent(s) removed under reduced pressure. The crude product (adsorbed on silica gel) was subjected to flash column chromatography using pure *n*-pentane as the eluent.

During optimization, different methods for preparation of the sodium phenolate solution (A, B, and C) were tested. The outcome is summarized in Table S1.

Method A: Commercially available sodium phenolate trihydrate (0.51, 1.02, or 1.70 g; corresponds to 3, 6, or 10 mmol) was dissolved in acetonitrile (10 mL).

Method B: A solution of sodium phenolate was prepared by adding an equimolar amount of sodium hydride to a solution of phenol (0.56 g, 6 mmol) in acetonitrile (10 mL).

Method C: A solution of sodium phenolate was prepared by adding an equimolar amount of sodium hydride to a solution of phenol (0.56 g, 6 mmol) in tetrahydrofurane (10 mL).

Table S1. Optimization of the two-step one-pot synthesis of diaryl ether **7**.

Entry	NMR yield **3m**-ClO_4 [%]	*n*(**3m**-ClO_4) [mmol]	*n*(PhO^-) [mmol]	$T_{arylation}$ [°C]	Method	Yield **7** one step [%] [a]	Yield **7** two steps [%] [b]
1	96	0.76	3	30 °C	A	31	30
2	93	0.74	6	70 °C	A	61	57
3	95	0.76	10	70 °C	A	37	35
4	94	0.75	6	70 °C	B	33	31
5	100	0.80	6	r.t.	B	49	49
6	99	0.79	6	50 °C	C	77	76

[a] Isolated yield calculated with respect to electro-generated **3m**-ClO_4. [b] Isolated yield calculated with respect to the iodoarene precursor.

3.4 Characterization of diaryl iodonium salts

Diphenyliodonium perchlorate (3a-ClO_4)

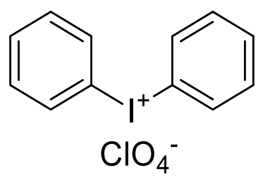

Brown powder (281 mg, 74% yield after passing 4 F per mole iodoarene). Eluent mixture: CH_2Cl_2/MeOH (97:3). ^1H NMR (400 MHz, CD_3CN): δ 8.15 – 8.03 (m, 4H), 7.76 – 7.66 (m, 2H), 7.60 – 7.49 (m, 4H). $^{13}C\{^1H\}$ NMR (101 MHz, CD_3CN): δ 136.4, 134.0, 133.4, 114.4 ppm. HRMS (ESI-TOF) m/z: calcd for $C_{12}H_{10}I^+$ 280.9822; found: 280.9825. Calcd for ClO_4^- 98.9490; found 98.9489.

(4-Iodophenyl)(phenyl)iodonium perchlorate (3b-ClO_4)

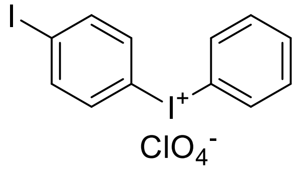

Synthesized using 2 mmol of iodobenzene with a transferred charge of 2 F per mole starting material. Off-white powder (375 mg, 74%). Eluent mixture: CH_2Cl_2/MeOH (97:3). ^1H NMR (400 MHz, CD_3CN) δ 8.10 – 8.06 (m, 2H), 7.89 (d, J = 8.7 Hz, 2H), 7.80 (d, J = 8.7 Hz, 2H), 7.75 – 7.69 (m, 1H), 7.58 – 7.52 (m, 2H). $^{13}C\{^1H\}$ NMR (101 MHz, CD_3CN): δ 142.4, 137.7, 136.5, 134.1, 133.5, 114.4, 113.5, 100.9. HRMS (ESI-TOF) m/z: calcd for $C_{12}H_9I_2^+$ 406.8789; found 406.8785. Calcd for ClO_4^- 98.9490; found 98.9488.

(4-Bromophenyl)(phenyl)iodonium perchlorate (3c-ClO_4)

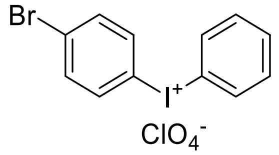

Brown powder (446 mg, 97% after passing 4 F per mole iodoarene; 174 mg, 38% after passing 2 F per mole iodoarene; 96% in scale-up experiment with 7 mmol of *p*-bromoiodobenzene). Eluent mixture: CH_2Cl_2/MeOH (97:3). ^1H NMR (400 MHz, CD_3CN) δ 8.10 – 8.05 (m, 2H), 7.95 (d, J = 8.8 Hz, 2H), 7.76 – 7.72 (m, 1H), 7.70 (d, J = 8.8 Hz, 2H), 7.59 – 7.53 (m, 2H). $^{13}C\{^1H\}$ NMR (101 MHz, CD_3CN) δ 138.0, 136.5, 136.4, 134.2, 133.5, 128.7, 114.6, 112.4. HRMS (ESI-TOF) m/z: calcd for $C_{12}H_9BrI^+$ 358.8927; found 358.8930. Calcd for ClO_4^- 98.9490; found 98.9488.

(4-Bromophenyl)(phenyl)iodonium tetrafluoroborate (3c-BF_4)

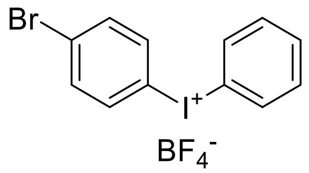

Brown powder (259 mg, 58% after passing 2 F per mole iodoarene; 348 mg, 78% after passing 4 F per mole iodoarene). Eluent mixture: CH_2Cl_2/MeOH (97:3). ^1H NMR (300 MHz, CD_3CN) δ 8.10 – 8.04 (m, 2H), 7.95 (d, J = 8.8 Hz, 2H), 7.77 – 7.72 (m, 1H), 7.70 (d, J = 8.9 Hz, 2H), 7.59 – 7.51 (m, 2H). ^{11}B NMR (128 MHz, CD_3CN): δ -1.16 (s). $^{13}C\{^1H\}$ NMR (101 MHz, CD_3CN): δ 138.0, 136.5, 136.4, 134.2, 133.5, 128.7, 114.4, 112.2. ^{19}F NMR (376 MHz, CD_3CN): δ -150.9. HRMS (ESI-TOF) m/z: calcd for $C_{12}H_9BrI^+$ 358.8927; found 358.8932. Calcd for $^{11}BF_4^-$ 87.0034; found 87.0031.

(4-Bromophenyl)(phenyl)iodonium triflate (3c-TfO)

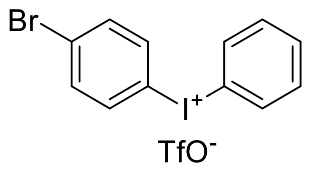

Brown powder (504 mg. 99% after passing 2 F per mole iodoarene; 473 mg, 93% after applying 4 F per mole). Eluent mixture: CH_2Cl_2/MeOH (97:3). ^1H NMR (400 MHz, CD_3CN) δ 8.11 – 8.06 (m, 2H), 7.96 (d, J = 8.9 Hz, 2H), 7.74 – 7.70 (m, 1H), 7.69 (d, J = 8.9 Hz, 2H), 7.57 – 7.51 (m, 2H). $^{13}C\{^1H\}$ NMR (101 MHz, CD_3CN): δ 138.0, 136.4, 136.3, 134.0, 133.4, 128.5, 121.7 (q, J_{CF} = 319.8 Hz), 114.80, 112.65. ^{19}F NMR (376 MHz, CD_3CN): δ -79.3. HRMS (ESI-TOF) m/z: calcd for $C_{12}H_9BrI^+$ 358.8927; found 358.8930. Calcd for TfO^- 148.9525; found 148.9523.

(4-Bromophenyl)(phenyl)iodonium hexafluorophosphate (3c-PF_6)

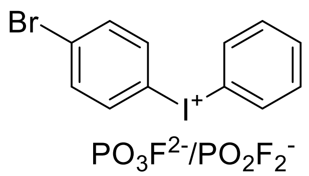

Electrolysis was carried out using $LiPF_6$ as supporting electrolyte and anion source, respectively. However, different anions – mainly monofluorophosphate and difluorophosphate – could be identified as counter ions in the product resulting from hydrolysis of hexafluorophosphate (for details, see section 6). Colorless resinous material (449 mg, 89% after application of 2 F per mole iodoarene precursor, column chromatography was performed with gradient elution using CH_2Cl_2/MeOH 32:1 – 12:1). ^1H NMR (400 MHz, DMSO-d_6) δ 8.23 (d, J = 7.7 Hz, 2H), 8.16 (d, J = 8.1 Hz, 2H), 7.73 (d, J = 7.8 Hz, 2H), 7.66 (t, J = 7.4 Hz, 1H), 7.52 (t, J = 7.6 Hz, 2H). $^{13}C\{^1H\}$ NMR (101 MHz, DMSO): δ 137.1, 135.2, 134.6, 132.1, 131.8, 126.2, 116.9, 115.2. ^{19}F NMR (376 MHz, DMSO): δ -71.39 (d, J = 906.0 Hz), -78.42 (d, J = 950.8 Hz). ^{31}P NMR (162 MHz, DMSO): δ -0.93, -7.06 (d, J = 906.0 Hz), -15.41 (t, J = 950.9 Hz).

(4-Bromophenyl)(phenyl)iodonium bis((trifluoromethyl)sulfonyl)amide (3c-Tf_2N)

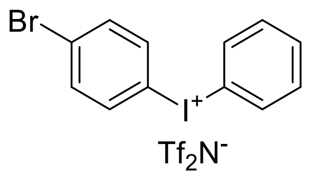

Brown oil (569 mg, 86% after applying 2 F per mole iodoarene precursor). Eluent mixture: CH_2Cl_2/MeOH (97:3). ^1H NMR (400 MHz, CD_3CN) δ 8.10 – 8.06 (m, 2H), 7.96 (d, J = 8.9 Hz, 2H), 7.76 – 7.71 (m, 1H), 7.70 (d, J = 8.9 Hz, 2H), 7.58 – 7.53 (m, 2H). $^{13}C\{^1H\}$ NMR: (101 MHz, CD_3CN) δ 138.0, 136.5, 134.2, 133.5, 128.7, 120.91 (q, J_{CF} = 320.6 Hz), 114.4, 112.2. ^{19}F NMR (376 MHz, CD_3CN): δ -80.11. HRMS (ESI-TOF) m/z: calcd for $C_{12}H_9BrI^+$ 358.8927; found 358.8932. Calcd for Tf_2N^- 279.9178; found 279.9180.

(4-Chlorophenyl)(phenyl)iodonium perchlorate (3d-ClO_4)

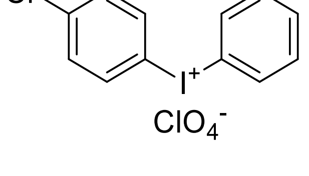

Beige powder (348 mg, 84% after applying 4 F per mole iodoarene precursor). Eluent mixture: CH_2Cl_2/MeOH (97:3). ^1H NMR (400 MHz, CD_3CN) δ 8.12 – 8.08 (m, 2H), 8.05 (d, J = 8.9 Hz, 2H), 7.75 – 7.69 (m, 1H), 7.58 – 7.55 (m, 1H), 7.54 (d, J = 8.9 Hz, 2H). $^{13}C\{^1H\}$ NMR (101 MHz, CD_3CN): δ 140.3, 138.0, 136.4, 134.1, 133.5, 133.4, 114.6, 111.5. HRMS (ESI-TOF) m/z: calcd for $C_{12}H_9ClI^+$ 314.9433; found 314.9437. Calcd for ClO_4^- 98.9490; found 98.9489.

(4-Fluorophenyl)(phenyl)iodonium perchlorate (3e-ClO_4)

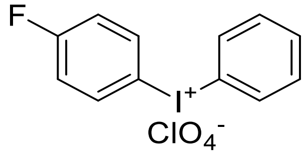

Red-brown powder (314 mg, 79% after applying 4 F per mole iodoarene precursor). Eluent mixture: CH_2Cl_2/MeOH (97:3). ^1H NMR (400 MHz, CD_3CN): δ 8.17 – 8.07 (m, 4H), 7.74 – 7.69 (m, 1H), 7.58 – 7.51 (m, 2H), 7.33 – 7.25 (m, 2H). $^{13}C\{^1H\}$ NMR (101 MHz, CD_3CN): δ 166.1 (d, $^1J_{CF}$ = 254.0 Hz), 139.3 (d, $^3J_{CF}$ = 9.4 Hz), 136.3, 134.0, 133.4, 120.8 (d, $^2J_{CF}$ = 23.7 Hz), 114.7, 107.7 (d, $^4J_{CF}$ = 3.3 Hz). ^{19}F NMR (376 MHz, CD_3CN): δ -106.11. HRMS (ESI-TOF) m/z: calcd for $C_{12}H_9FI^+$ 298.9728; found 298.9726. Calcd for ClO_4^- 98.9490; found 98.9488.

Phenyl(*p*-tolyl)iodonium perchlorate (3f-ClO_4)

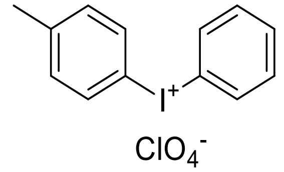

Off-white powder (264 mg, 67% after applying 4 F per mole iodoarene precursor). Eluent mixture: CH_2Cl_2/MeOH (97:3). ^1H NMR (400 MHz, CD_3CN): δ 8.12 – 8.05 (m, 2H), 8.00 – 7.93 (m, 2H), 7.74 – 7.67 (m, 1H), 7.57 – 7.50 (m, 2H), 7.38 – 7.31 (m, 2H), 2.39 (s, 3H). $^{13}C\{^1H\}$ NMR (101 MHz, CD_3CN): δ 145.5, 136.4, 136.2, 134.1, 133.9, 133.3, 114.6, 110.6, 21.4. HRMS (ESI-TOF) m/z: calcd for $C_{13}H_{12}I^+$ 294.9979; found 294.9983. Calcd for ClO_4^- 98.9490; found 98.9489.

(4-(*tert*-butyl)phenyl)(phenyl)iodonium perchlorate (3g-ClO_4)

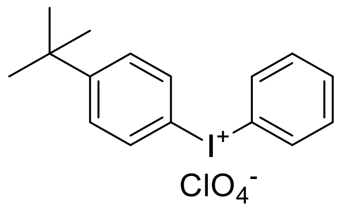

Off-white powder (287 mg, 66% after applying 4 F per mole iodoarene precursor). Eluent mixture: CH_2Cl_2/MeOH (97:3). ^1H NMR (400 MHz, CD_3CN) δ 8.11 – 8.06 (m, 2H), 8.01 (d, *J* = 8.9 Hz, 2H), 7.75 – 7.69 (m, 1H), 7.59 (d, *J* = 8.9 Hz, 2H), 7.57 – 7.51 (m, 2H), 1.30 (s, 9H). $^{13}C\{^1H\}$ NMR: (101 MHz, CD_3CN) δ 158.0, 136.3, 136.2, 133.9, 133.4, 130.8, 114.5, 110.8, 35.9, 31.0. HRMS (ESI-TOF) m/z: Calcd for $C_{16}H_{18}I^+$ 337.0448; found 337.0445. Calcd for ClO_4^- 98.9490; found 98.9492.

Phenyl(4-(trifluoromethyl)phenyl)iodonium perchlorate (3h-ClO_4)

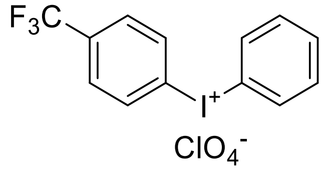

Off-white solid (206 mg, 46% after applying 4 F per mole iodoarene precursor and using 2 equiv. benzene as coupling partner). Eluent mixture: CH_2Cl_2/MeOH (97:3). ^1H NMR (400 MHz, CD_3CN) δ 8.22 (d, *J* = 8.2 Hz, 2H), 8.13 – 8.03 (m, 2H), 7.81 (d, *J* = 8.2 Hz, 2H), 7.76 – 7.71 (m, 1H), 7.59 – 7.53 (m, 2H). $^{13}C\{^1H\}$ NMR (101 MHz, CD_3CN): δ 136.93, 136.70, 134.50 (q, *J* = 33.2 Hz), 134.12, 133.51, 129.84 (q, *J* = 3.7 Hz), 124.34 (q, *J* = 272.2 Hz), 119.24, 115.60. ^{19}F NMR (376 MHz, CD_3CN) δ -63.84. HRMS (ESI-TOF) m/z: calcd for $C_{13}H_9F_3I^+$ 348.9696; found 348.9697. Calcd for ClO_4^- 98.9490; found 98.9489.

(4-Cyanophenyl)(phenyl)iodonium perchlorate (3i-ClO_4)

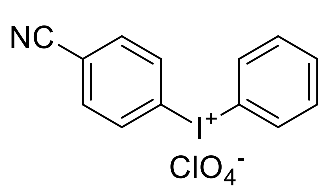

Brown solid (211 mg, 52% after applying 4 F per mole iodoarene precursor and using 2 equiv. benzene as coupling partner). Eluent mixture: CH_2Cl_2/MeOH (97:3). ^1H NMR (400 MHz, MeOD) δ 8.34 (d, *J* = 8.8 Hz, 2H), 8.26 – 8.20 (m, 2H), 7.87 (d, *J* = 8.8 Hz, 2H), 7.76 – 7.70 (m, 1H), 7.60 – 7.54 (m, 2H). $^{13}C\{^1H\}$ NMR (101 MHz, MeOD): δ 137.1, 136.8, 136.2, 134.0, 133.4, 120.3,

118.0, 117.4, 116.2. HRMS (ESI-TOF) m/z: calcd for $C_{13}H_9NI^+$ 305.9775; found 305.9769. Calcd for ClO_4^- 98.9490; found 98.9492.

(4-Bromophenyl)(*p*-tolyl)iodonium perchlorate (3j-ClO$_4$)

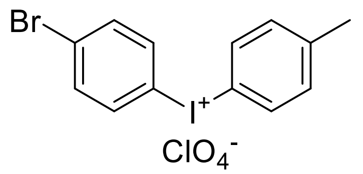

Light-brown powder (340 mg, 80% after applying 4 F per mole iodoarene precursor and using 2 equiv. toluene as coupling partner; isolated from an aliquot of 90% of the anolyte); formation of *ortho*-tolyl isomer could be observed in ca. 0.08 mmol quantity (molar ratio ortho/para = 1:9) whereas the yield is referred to the total isolated mass of iodonium salt containing both isomers. Eluent mixture: CH_2Cl_2/MeOH (97:3). ^{1}H NMR (400 MHz, CD_3CN) δ 7.98 – 7.91 (m, 4H), 7.69 (d, *J* = 8.8 Hz, 2H), 7.40 – 7.34 (m, 2H), 2.41 (s, 3H). ^{13}C{^{1}H} NMR (101 MHz, CD_3CN): δ 145.6, 137.8, 136.4, 136.3, 134.2, 128.5, 113.1, 111.2, 21.4. Detected signals of presumed *ortho*-tolyl isomer: ^{1}H NMR (400 MHz, CD_3CN): δ 8.16 (dd, *J* = 8.1, 1.2 Hz, 1H), 7.90 – 7.86 (m, 2H), 7.63 (dd, *J* = 7.4, 1.2 Hz, 1H), 7.60 – 7.56 (m, 1H), 2.60 (s, 3H), ^{13}C{^{1}H} NMR (101 MHz, CD_3CN) 138.4, 137.6, 134.9, 133.2, 130.9, 25.8. HRMS (ESI-TOF) m/z: Calcd for $C_{13}H_{11}BrI^+$ 372.9084; found 372.9080. Calcd for ClO_4^- 98.9490; found 98.9491.

(4-Bromophenyl)(mesityl)iodonium perchlorate (3k-ClO$_4$)

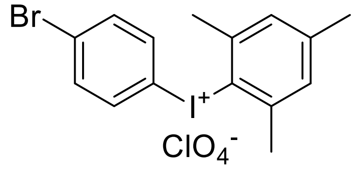

Brown powder (396 mg, 79% after applying 4 F per mole iodoarene precursor). Eluent mixture: CH_2Cl_2/MeOH (97:3). ^{1}H NMR (400 MHz, CD_3CN) δ 7.71 (d, *J* = 8.9 Hz, 2H), 7.64 (d, *J* = 8.9 Hz, 2H), 7.23 (s, 2H), 2.59 (s, 6H), 2.35 (s, 3H). ^{13}C{^{1}H} NMR (101 MHz, CD_3CN): δ 146.2, 143.6, 136.8, 136.3, 131.4, 128.0, 121.6, 110.9, 27.2, 21.0. HRMS (ESI-TOF) m/z: calcd for $C_{15}H_{15}BrI^+$ 400.9397; found 400.9406. Calcd for ClO_4^- 98.9490; found 98.9491.

(4-Bromophenyl)(2,5-dimethylphenyl)iodonium perchlorate (3l-ClO$_4$)

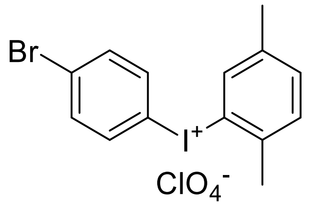

Brown powder (221 mg, 45% after applying 4 F per mole iodoarene precursor and using 2 equiv. *p*-xylene as coupling partner). Eluent mixture: CH_2Cl_2/MeOH (97:3). ^{1}H NMR (400 MHz, DMSO-d$_6$) δ 8.24 (s, 1H), 8.11 (d, *J* = 8.6 Hz, 2H), 7.73 (d, *J* = 8.6 Hz, 2H), 7.46 – 7.43 (m, 1H), 7.42 – 7.39 (m, 1H), 2.54 (s, 3H), 2.31 (s, 3H). ^{13}C{^{1}H} NMR (101 MHz, DMSO-*d*$_6$): δ 139.1, 137.3, 137.0, 136.8, 134.6, 133.5, 131.0, 126.0, 121.3, 114.3, 24.4, 20.0. HRMS (ESI-TOF) m/z: calcd for $C_{14}H_{13}BrI^+$ 386.9240; found 386.9243. Calcd for ClO_4^- 98.9490; found 98.9488.

(4-Bromophenyl)(4-(*tert*-butyl)phenyl)iodonium perchlorate (3m-ClO$_4$)

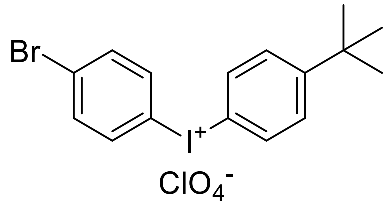

Off-white solid (357 mg, 77% after applying 4 F per mole iodoarene precursor and using 2 equiv. of *tert*-butylbenzene as coupling partner; isolated from an aliquot of 90% of the anolyte). Eluent: CH_2Cl_2/MeOH (97:3). ^{1}H NMR (400 MHz, MeOD) δ 8.09 (d, *J* = 8.9 Hz, 2H), 8.06 (d, *J* = 8.7 Hz, 2H), 7.69 (d, *J* = 8.7 Hz, 2H), 7.59 (d, *J* = 8.8 Hz, 2H), 1.32 (s, 9H). ^{13}C{^{1}H} NMR (101 MHz, MeOD) δ 158.0, 138.0, 136.3, 136.2, 130.6, 128.5, 114.3, 112.8,

36.1, 31.3. HRMS (ESI-TOF) m/z: calcd for $C_{16}H_{17}BrI^+$ 414.9553; found 414.9550. Calcd for ClO_4^- 98.9490; found 98.9487.

(4-Bromophenyl)(4-chlorophenyl)iodonium perchlorate (3n-ClO₄)

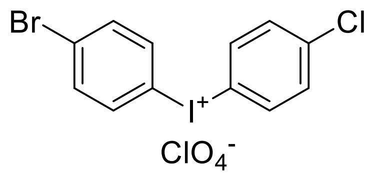

Off-white solid (409 mg, 92% after applying 4 F per mole iodoarene precursor and using 2 equiv. of chlorobenzene as coupling partner; isolated from an aliquot of 90% of the anolyte). Eluent: CH_2Cl_2/MeOH (97:3). ^1H NMR (400 MHz, DMSO-d_6) δ 8.25 (d, J = 8.8 Hz, 2H), 8.17 (d, J = 8.7 Hz, 2H), 7.76 (d, J = 8.7 Hz, 2H), 7.63 (d, J = 8.8 Hz, 2H). $^{13}C\{^1H\}$ NMR (101 MHz, DMSO-d_6) δ 137.5, 137.1, 137.0, 134.7, 131.8, 126.4, 115.5, 114.7. HRMS (ESI-TOF) m/z: calcd for $C_{12}H_8BrClI^+$ 392.8537; found 392.8542. Calcd for ClO_4^- 98.9490; found 98.9492.

Bis(4-bromophenyl)iodonium perchlorate (3o-ClO₄)

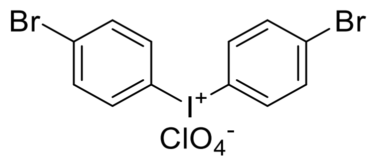

Off-white powder (522 mg, 97% after applying 4 F per mole iodoarene precursor using 2 equiv. bromobenzene as coupling partner). Eluent mixture: CH_2Cl_2/MeOH (97:3). ^1H NMR (400 MHz, CD_3CN) δ 7.96 (d, J = 8.8 Hz, 4H), 7.70 (d, J = 8.8 Hz, 4H). $^{13}C\{^1H\}$ NMR (101 MHz, CD_3CN): δ 138.1, 136.4, 128.8, 112.6. HRMS (ESI-TOF) m/z: $[M-ClO_4]^+$ calcd for $C_{12}H_8Br_2I^+$ 436.8032; found 436.8035. $[ClO_4]^-$ calcd for ClO_4^- 98.9490; found 98.9488.

(4-(*tert*-Butyl)phenyl)(mesityl)iodonium perchlorate (3p-ClO₄)

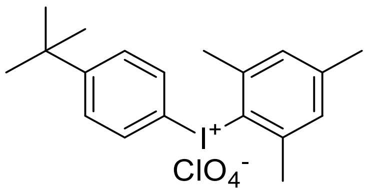

Off-white powder (474 mg, 99% after applying 4 F per mole iodoarene using 2 equiv. mesitylene as reactant). Eluent mixture: CH_2Cl_2/MeOH (97:3). ^1H NMR (400 MHz, CD_3CN) δ 7.79 (d, J = 8.9 Hz, 2H), 7.54 (d, J = 8.9 Hz, 2H), 7.22 (s, 2H), 2.61 (s, 6H), 2.34 (s, 3H), 1.29 (s, 9H). $^{13}C\{^1H\}$ NMR (101 MHz, CD_3CN): δ 157.6, 145.9, 143.5, 135.2, 131.3, 130.8, 121.5, 109.3, 35.8, 31.0, 27.2, 21.0. HRMS (ESI-TOF) m/z: calcd for $C_{19}H_{24}I^+$ 379.0918; found 379.0915. Calcd for ClO_4^- 98.9490; found 98.9488.

Dibenzo[*b*,*d*]iodol-5-ium perchlorate (5a-ClO₄)

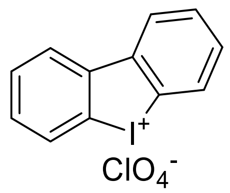

Pale yellow powder (264 mg, 70% after applying 2 F per mole iodoarene precursor). Eluent mixture: CH_2Cl_2/MeOH (97:3). ^1H NMR (400 MHz, CD_3CN) δ 8.31 (dd, J = 8.0, 1.5 Hz, 2H), 8.14 (d, J = 7.3 Hz, 2H), 7.87 (dd, J = 7.6, 7.6 Hz, 2H), 7.74 – 7.68 (m, 2H). $^{13}C\{^1H\}$ NMR (101 MHz, CD_3CN): δ 143.0, 132.6, 132.3, 131.5, 128.4, 122.0. HRMS (ESI-TOF) m/z: calcd for $C_{12}H_8I^+$ 278.9666; found 278.9672. Calcd for ClO_4^- 98.9490; found 98.9488.

1-Methyldibenzo[*b,d*]iodol-5-ium perchlorate (5b-ClO_4)

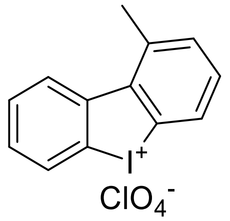

Colorless powder (172 mg, 44% after applying 2 F per mole iodoarene precursor). Eluent mixture: CH_2Cl_2/MeOH (97:3). ^1H NMR (300 MHz, DMSO-d$_6$) δ 8.53 (d, *J* = 8.2 Hz, 1H), 8.34 (dd, *J* = 8.2, 1.3 Hz, 1H), 8.19 (d, *J* = 8.1 Hz, 1H), 7.89 (ddd, *J* = 8.3 Hz, 7.2 Hz, 1.3 Hz, 1H), 7.75 – 7.70 (m, 2H), 7.58 (dd, *J* = 7.8, 7.8 Hz, 1H), 2.90 (s, 3H). $^{13}C\{^1H\}$ NMR (101 MHz, DMSO-*d*$_6$) δ 142.6, 139.8, 139.3, 134.4, 130.7, 130.6, 130.4, 130.1, 129.7, 128.5, 122.2, 120.7, 23.2. HRMS (ESI-TOF) m/z: calcd for $C_{13}H_{10}I^+$ 292.9822; found 278.9817. Calcd for ClO_4^- 98.9490; found 98.9490.

7-Chloro-2-methyldibenzo[*b,d*]iodol-5-ium perchlorate (5c-ClO_4)

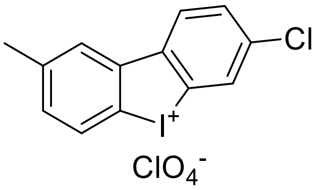

Colorless powder (175 mg, 41% after applying 2 F per mole iodoarene precursor). Eluent mixture: CH_2Cl_2/MeOH (97:3). ^1H NMR (400 MHz, DMSO-d$_6$): δ 8.44 (d, *J* = 8.6 Hz, 1H), 8.34 (s, 1H), 8.19 (d, *J* = 2.1 Hz, 1H), 8.05 (d, *J* = 8.4 Hz, 1H), 7.94 (dd, *J* = 8.4, 2.1 Hz, 1H), 7.56 (dd, *J* = 8.6, 1.8 Hz, 1H), 2.51 (s, 3H). $^{13}C\{^1H\}$ NMR (101 MHz, DMSO-*d*$_6$): δ 141.1, 140.7, 140.5, 134.3, 132.3, 131.0, 130.0, 129.8, 127.8, 127.6, 122.3, 118.4, 20.76. HRMS (ESI-TOF) m/z: calcd for $C_{13}H_9ClI^+$ 326.9432; found 326.9433. Calcd for ClO_4^- 98.9490; found 98.9488.

3-chlorodibenzo[*b,d*]iodol-5-ium perchlorate (5d-ClO_4)

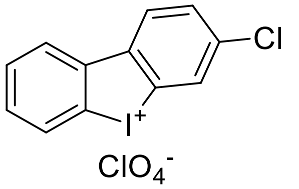

Colorless powder (148 mg, 62% after applying 2 F per mole iodoarene, synthesized using 0.58 mmol starting material). Eluent mixture: CH_2Cl_2/MeOH (97:3). ^1H NMR (400 MHz, CD_3CN): δ 8.24 (dd, *J* = 7.9, 1.6 Hz, 1H), 8.21 (d, *J* = 8.6 Hz, 1H), 8.15 (dd, *J* = 8.4, 1.0 Hz, 1H), 8.12 (d, *J* = 2.0 Hz, 1H), 7.88 – 7.82 (m, 2H), 7.73 – 7.68 (m, 1H). $^{13}C\{^1H\}$ NMR (101 MHz, CD_3CN) δ 142.0, 141.9, 136.8, 132.9, 132.9, 132.5, 131.5, 130.9, 129.1, 128.7, 122.3, 121.8. HRMS (ESI-TOF) m/z: calcd for $C_{12}H_7ClI^+$ 312.9276; found 312.9282. Calcd for ClO_4^- 98.9490; found 98.9492.

1-Bromo-4-phenoxybenzene (7)

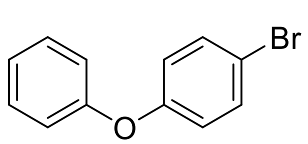

Colorless oil (152 mg, 76% yield over two steps, calculated with respect to the iodoarene precursor; purification by flash column chromatography using *n*-pentane as eluent). ^1H NMR (300 MHz, CD_2Cl_2) δ 7.46 (d, *J* = 9.0 Hz, 2H), 7.42 – 7.34 (m, 2H), 7.20 – 7.12 (m, 1H), 7.06 – 7.00 (m, 2H), 6.91 (d, *J* = 9.0 Hz, 2H). $^{13}C\{^1H\}$ NMR (75 MHz, CD_2Cl_2) δ 157.1, 157.1, 133.0, 130.3, 124.2, 120.8, 119.4, 115.9. NMR spectra are in agreement with the literature.[10]

[10] Kikushima, K.; Miyamoto, N.; Watanabe, K.; Koseki, D.; Kita, Y.; Dohi, T. Ligand- and Counterion-Assisted Phenol *O*-Arylation with TMP-Iodonium(III) Acetates. *Org. Lett.* **2022**, *24*, 1924-1928.

4. NMR spectra

Diphenyliodonium perchlorate (**3a**-ClO_4), ^{1}H NMR spectrum (400 MHz, CD_3CN).

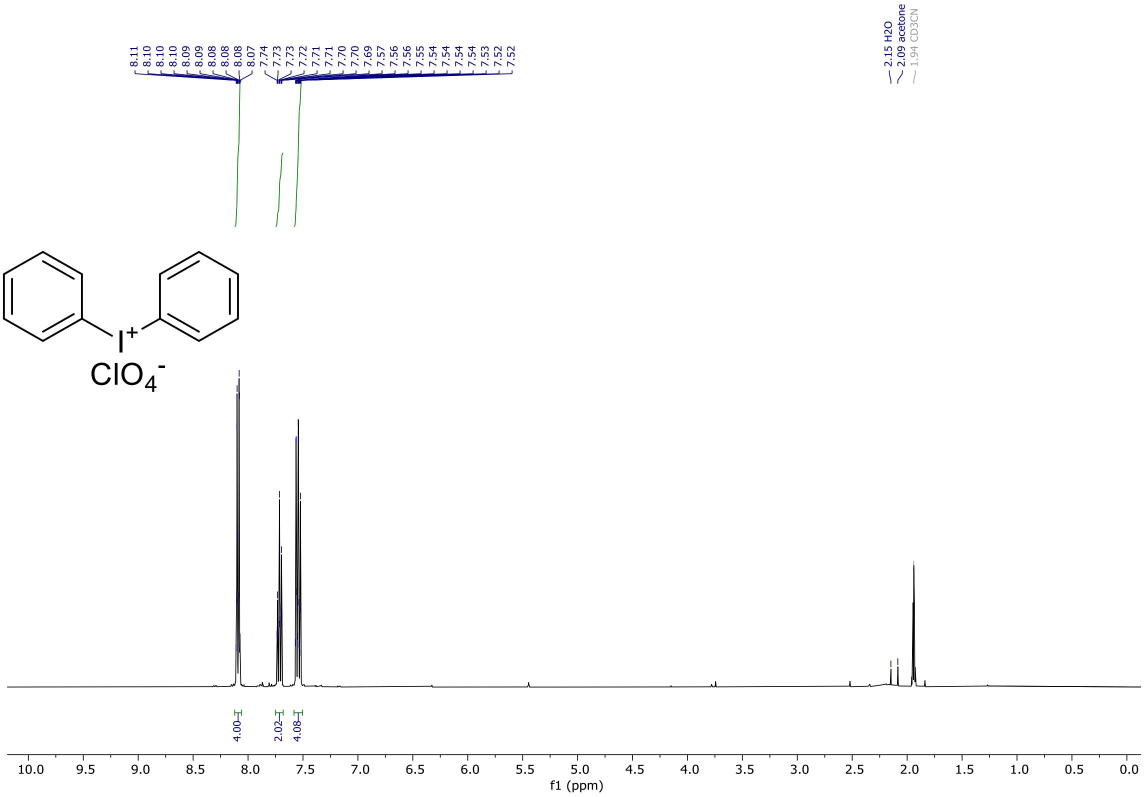

Diphenyliodonium perchlorate (**3a**-ClO_4), $^{13}C\{^{1}H\}$ NMR spectrum (101 MHz, CD_3CN)

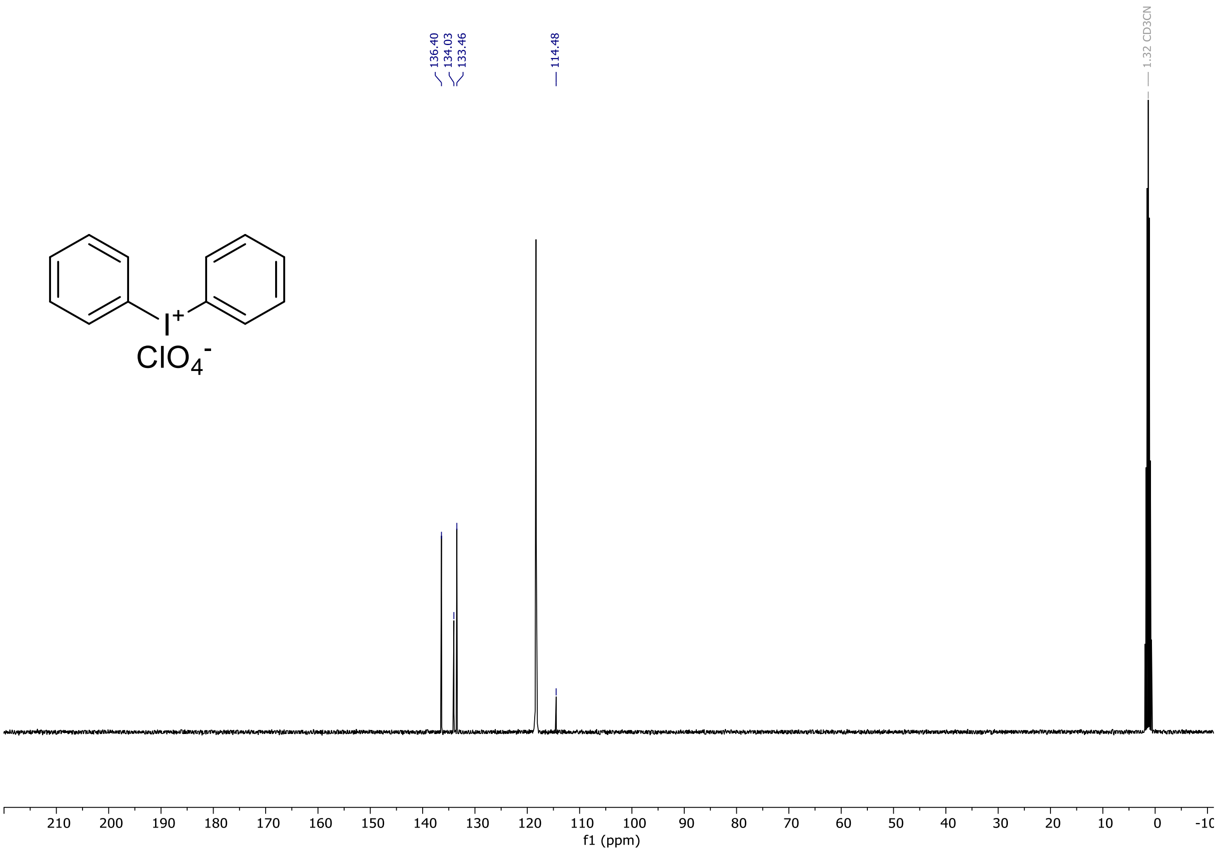

(4-Iodophenyl)(phenyl)iodonium perchlorate (**3b**-ClO_4), ^{1}H NMR spectrum (400 MHz, CD_3CN)

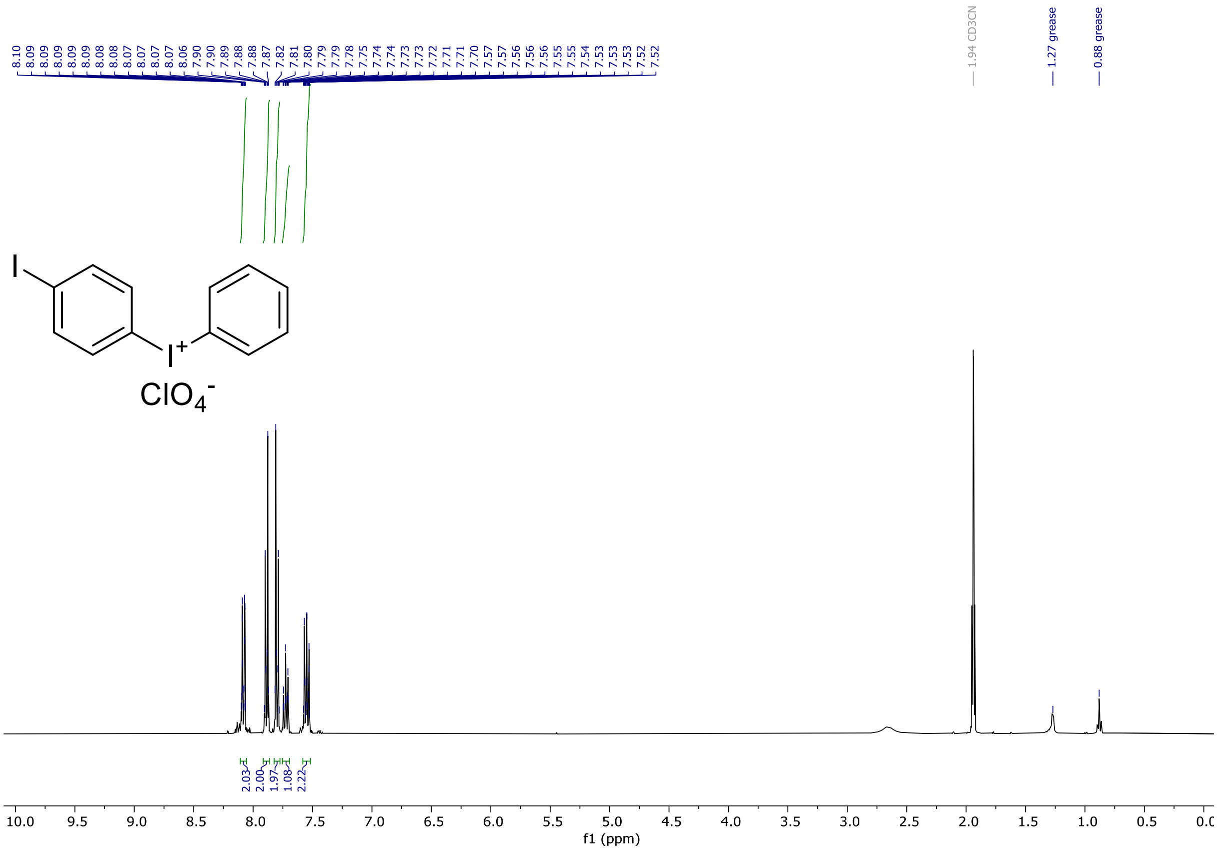

(4-Iodophenyl)(phenyl)iodonium perchlorate (**3b**-ClO_4), $^{13}C\{^{1}H\}$ NMR spectrum (400 MHz, CD_3CN)

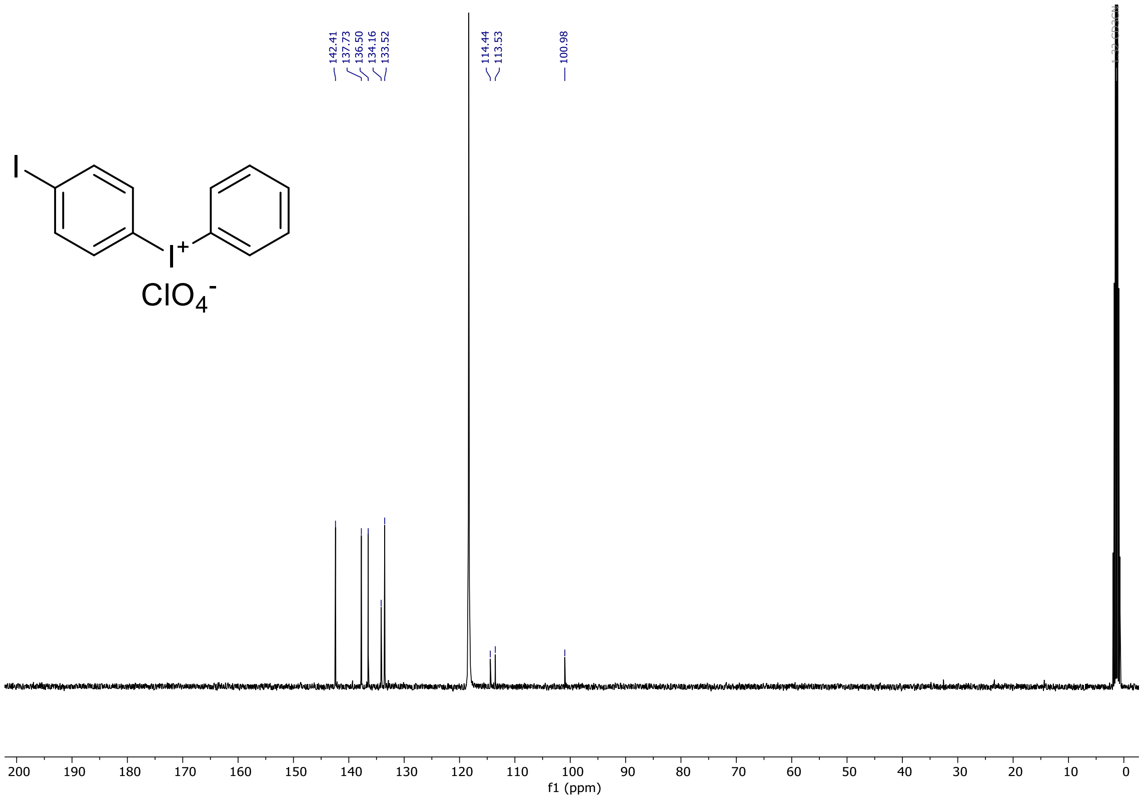

(4-Bromophenyl)(phenyl)iodonium perchlorate (**3c**-ClO_4), ^1H NMR spectrum (400 MHz, CD_3CN)

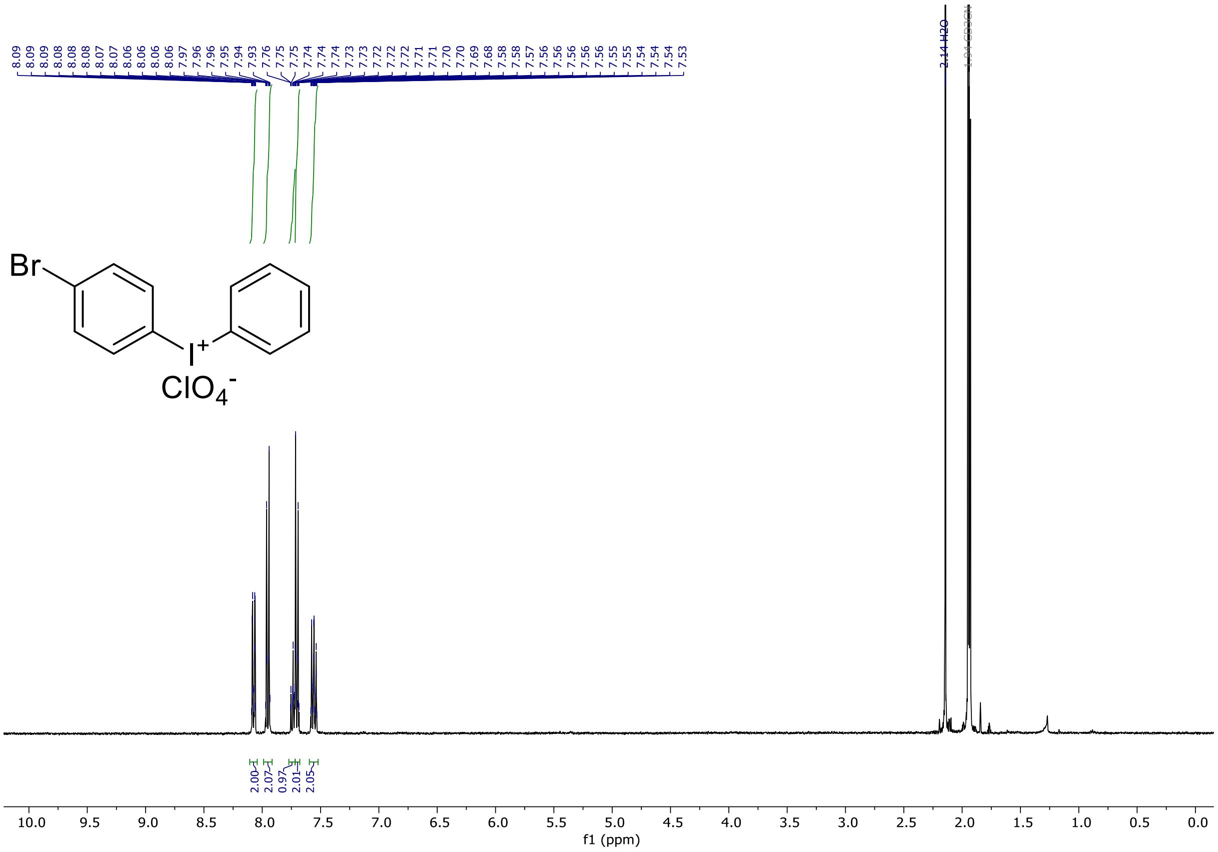

(4-Bromophenyl)(phenyl)iodonium perchlorate (**3c**-ClO_4), $^{13}C\{^1H\}$ NMR spectrum (101 MHz, CD_3CN)

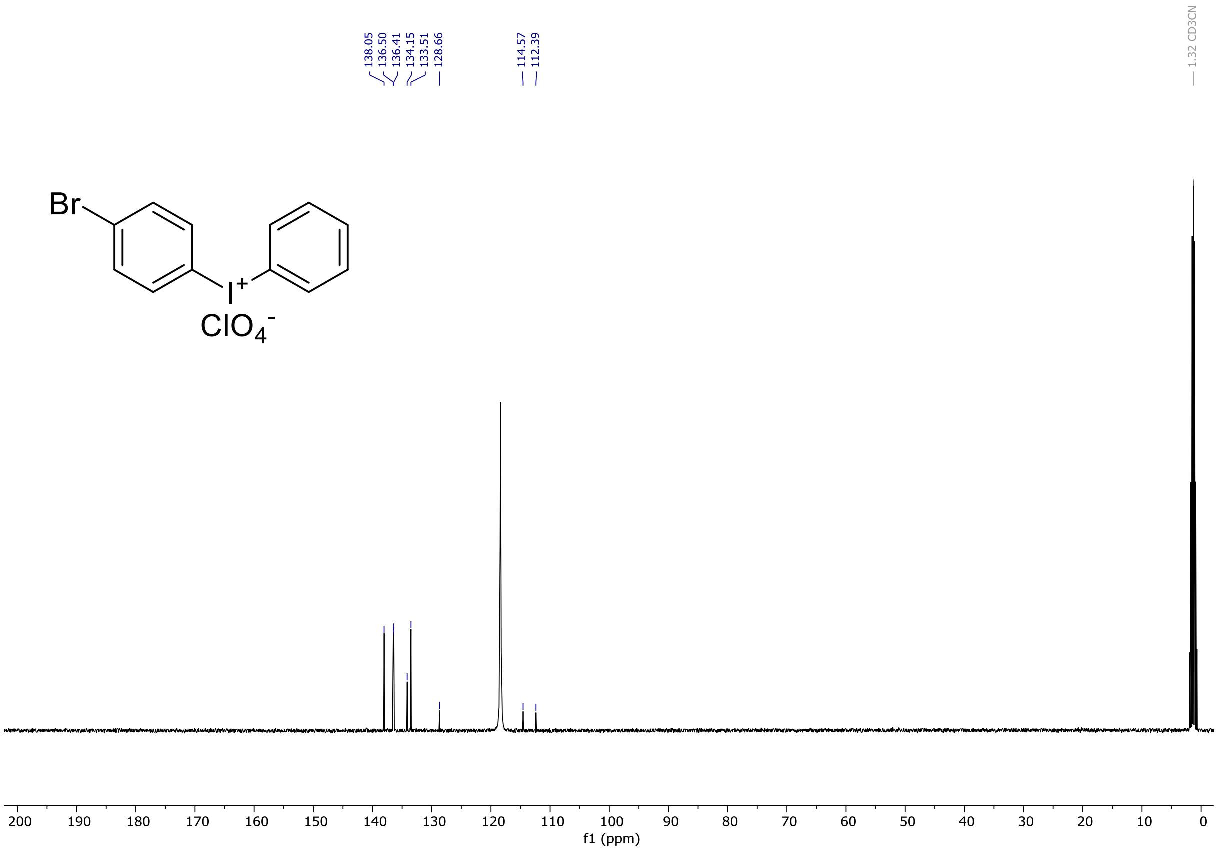

(4-Bromophenyl)(phenyl)iodonium tetrafluoroborate (**3c**-BF_4), ^1H NMR spectrum (300 MHz, CD_3CN)

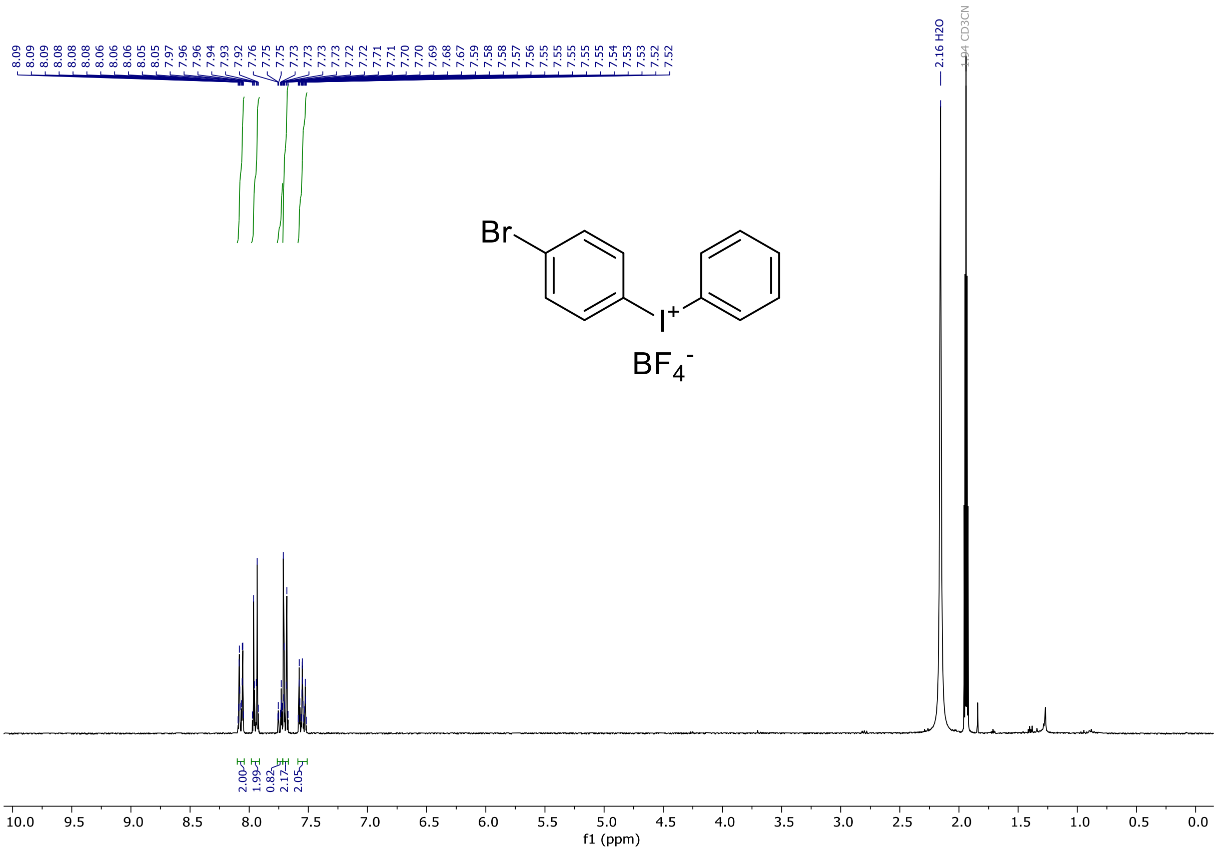

(4-Bromophenyl)(phenyl)iodonium tetrafluoroborate (**3c**-BF_4), ^{11}B NMR spectrum (128 MHz, CD_3CN)

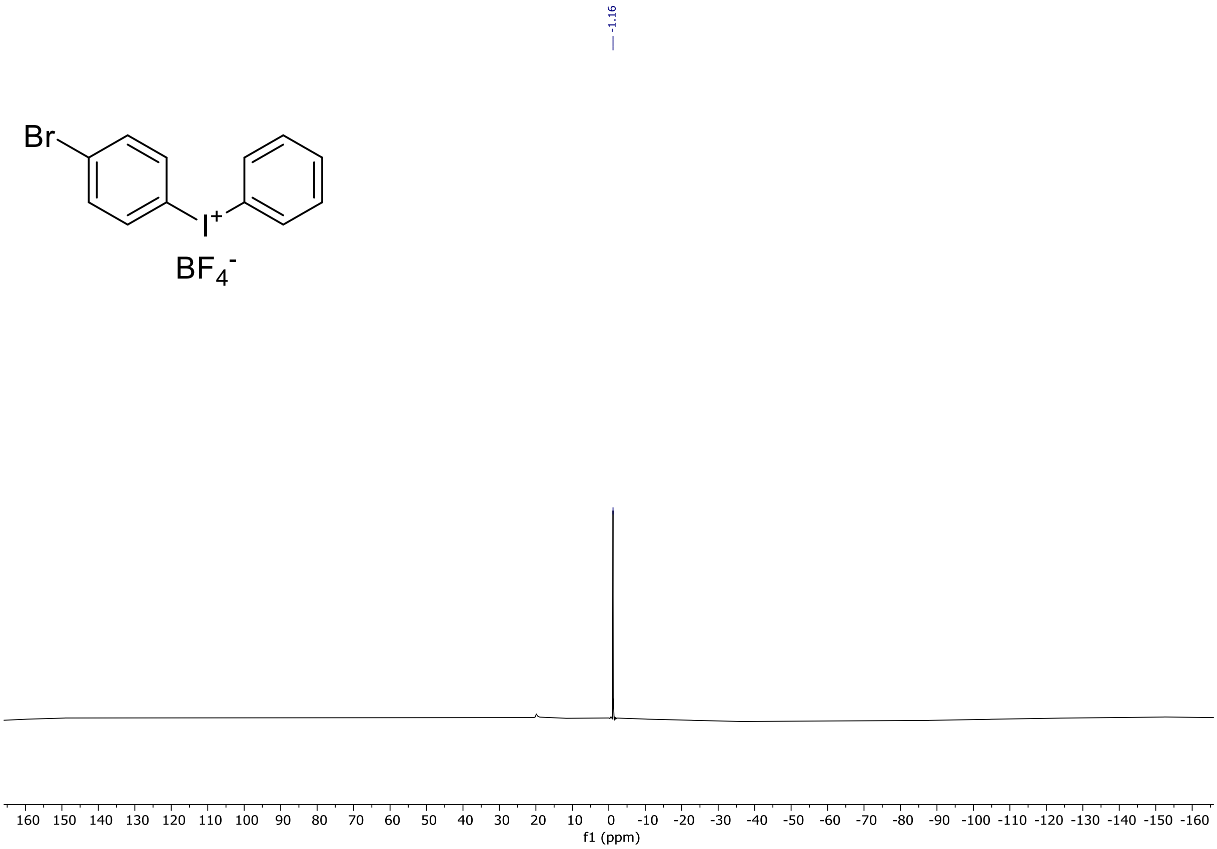

(4-Bromophenyl)(phenyl)iodonium tetrafluoroborate (**3c**-BF_4), $^{13}C\{^1H\}$ NMR spectrum (101 MHz, CD_3CN)

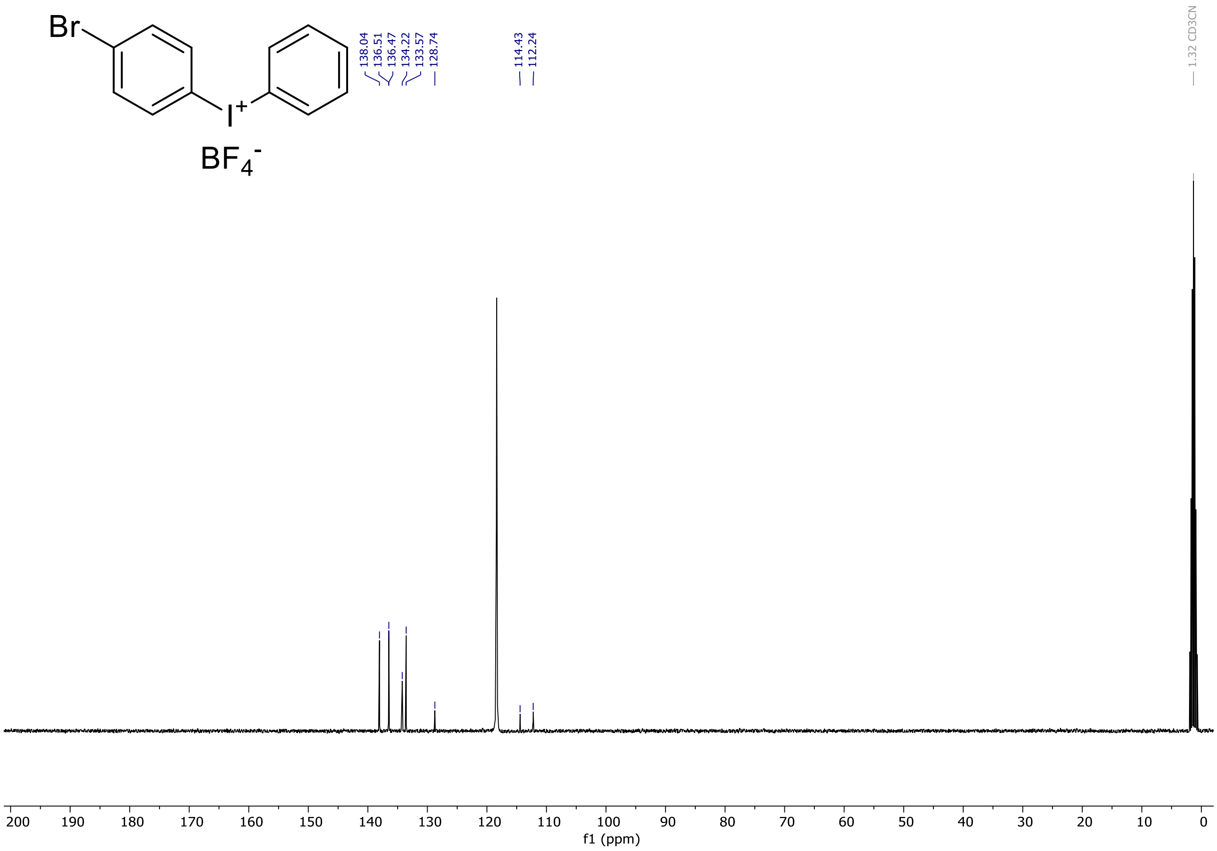

(4-Bromophenyl)(phenyl)iodonium tetrafluoroborate (**3c**-BF_4), ^{19}F NMR spectrum (376 MHz, CD_3CN)

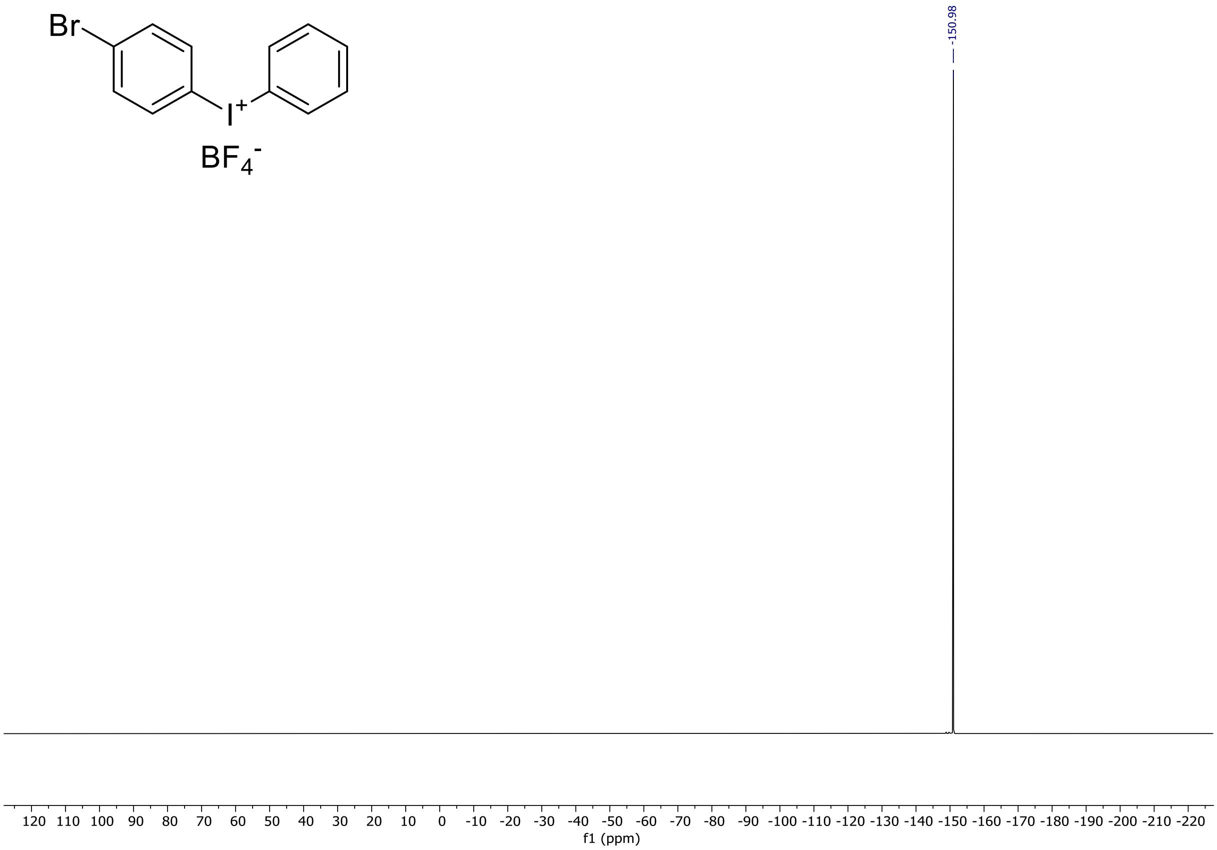

(4-Bromophenyl)(phenyl)iodonium triflate (**3c**-TfO), ^{1}H NMR spectrum (400 MHz, CD_3CN)

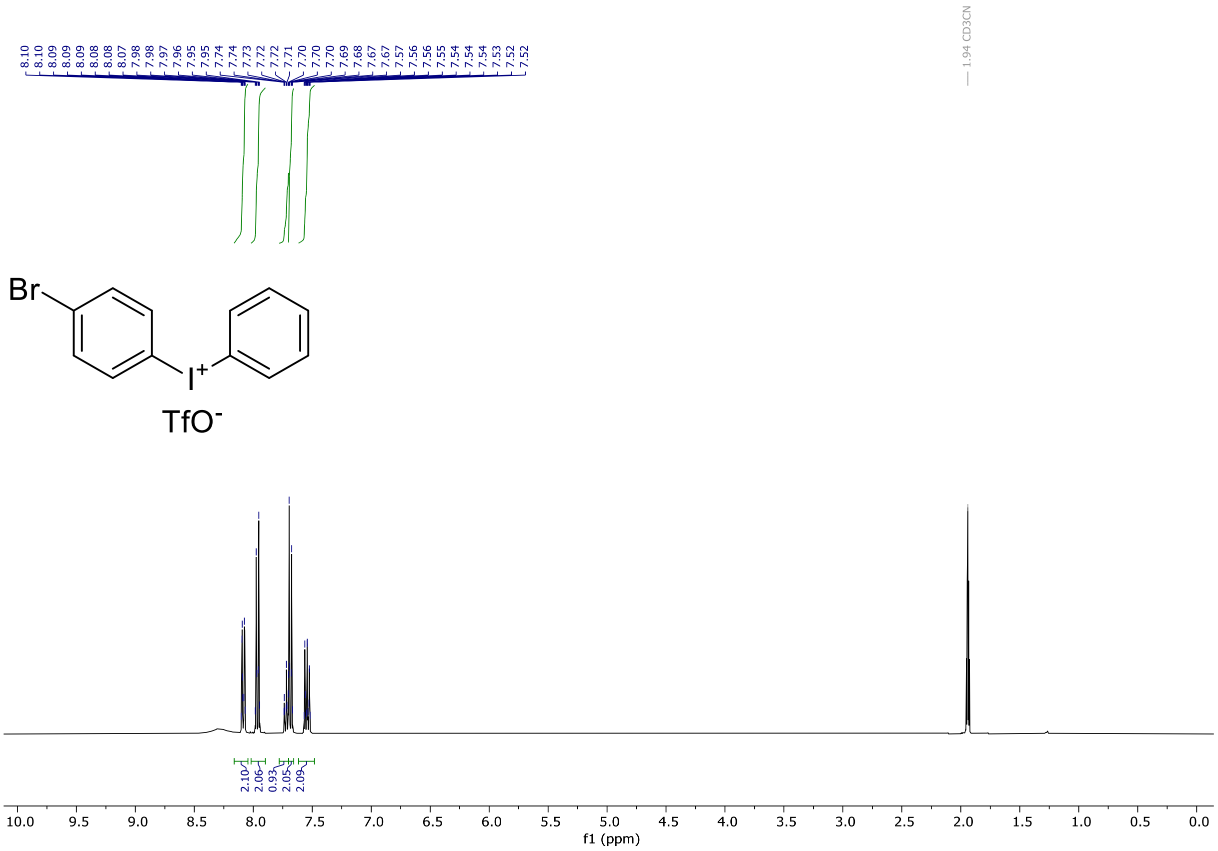

(4-Bromophenyl)(phenyl)iodonium triflate (**3c**-TfO), $^{13}C\{^{1}H\}$ NMR spectrum (101 MHz, CD_3CN)

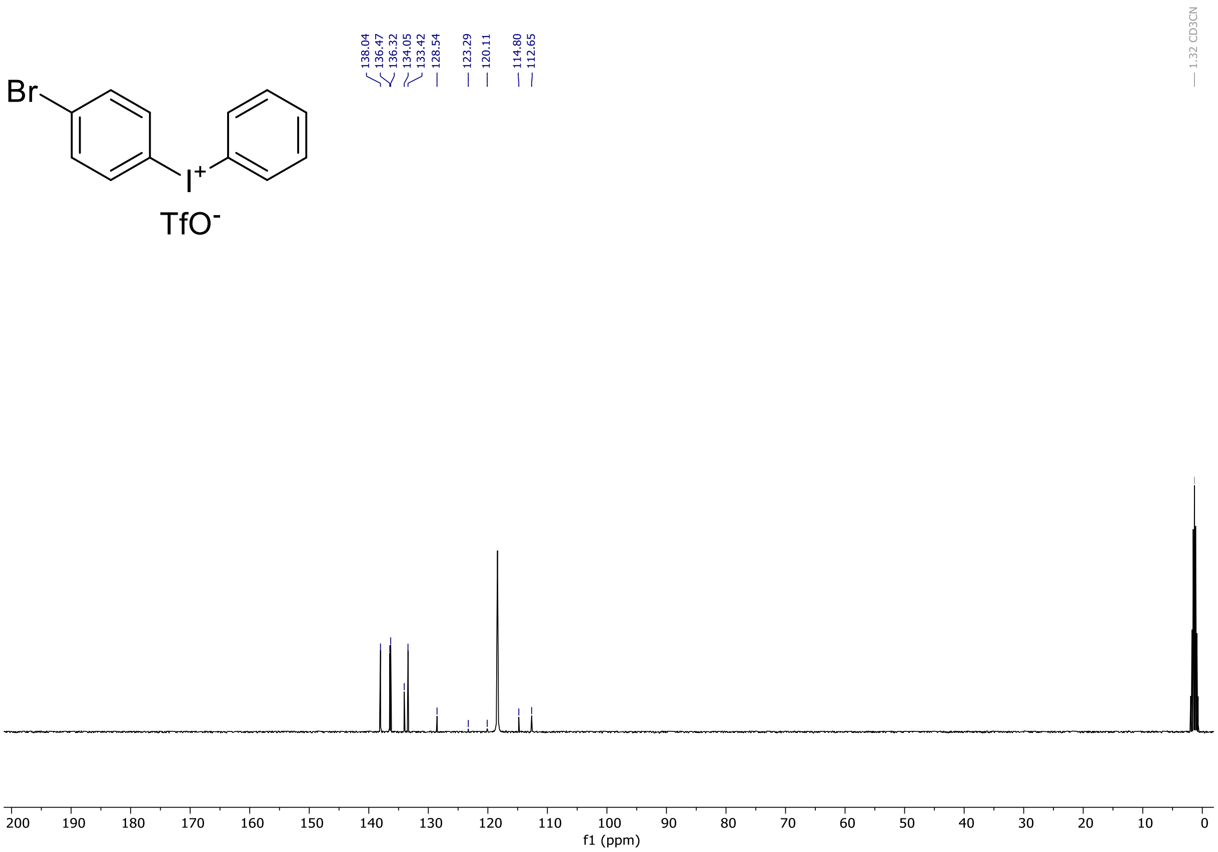

(4-Bromophenyl)(phenyl)iodonium triflate (**3c**-TfO), ^{19}F NMR spectrum (376 MHz, CD_3CN)

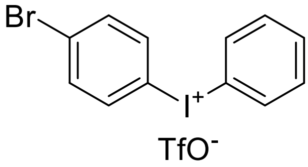

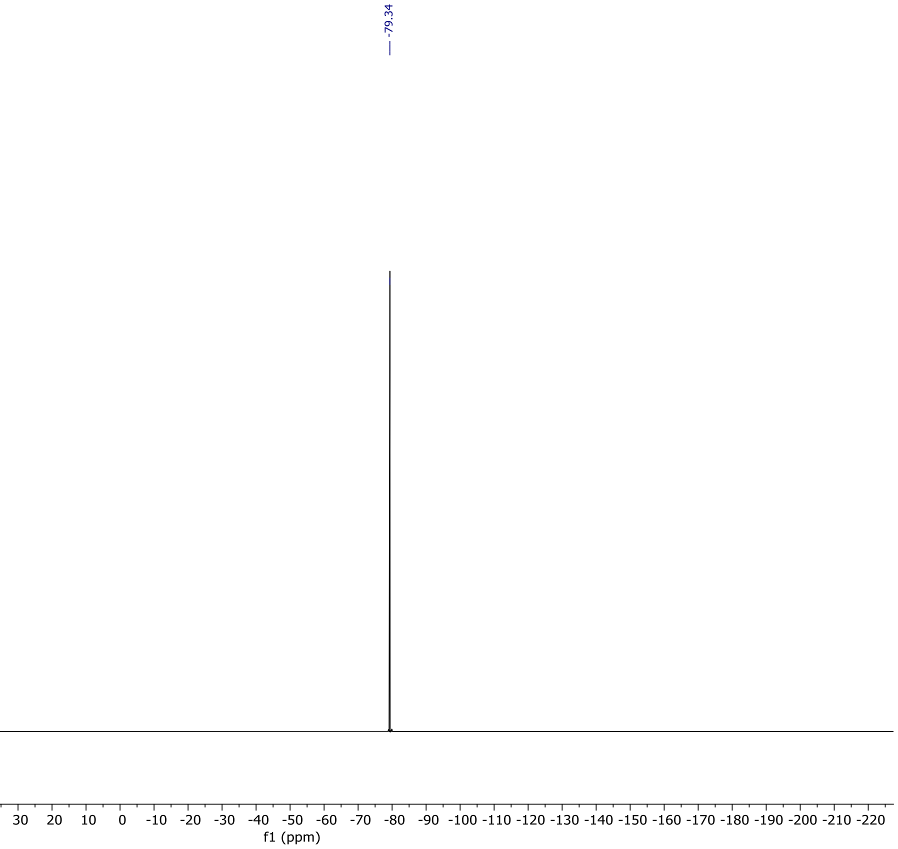

(4-Bromophenyl)(phenyl)iodonium hexafluorophosphate (**3c**-PF_6), ^{1}H NMR spectrum (400 MHz, DMSO-d_6)

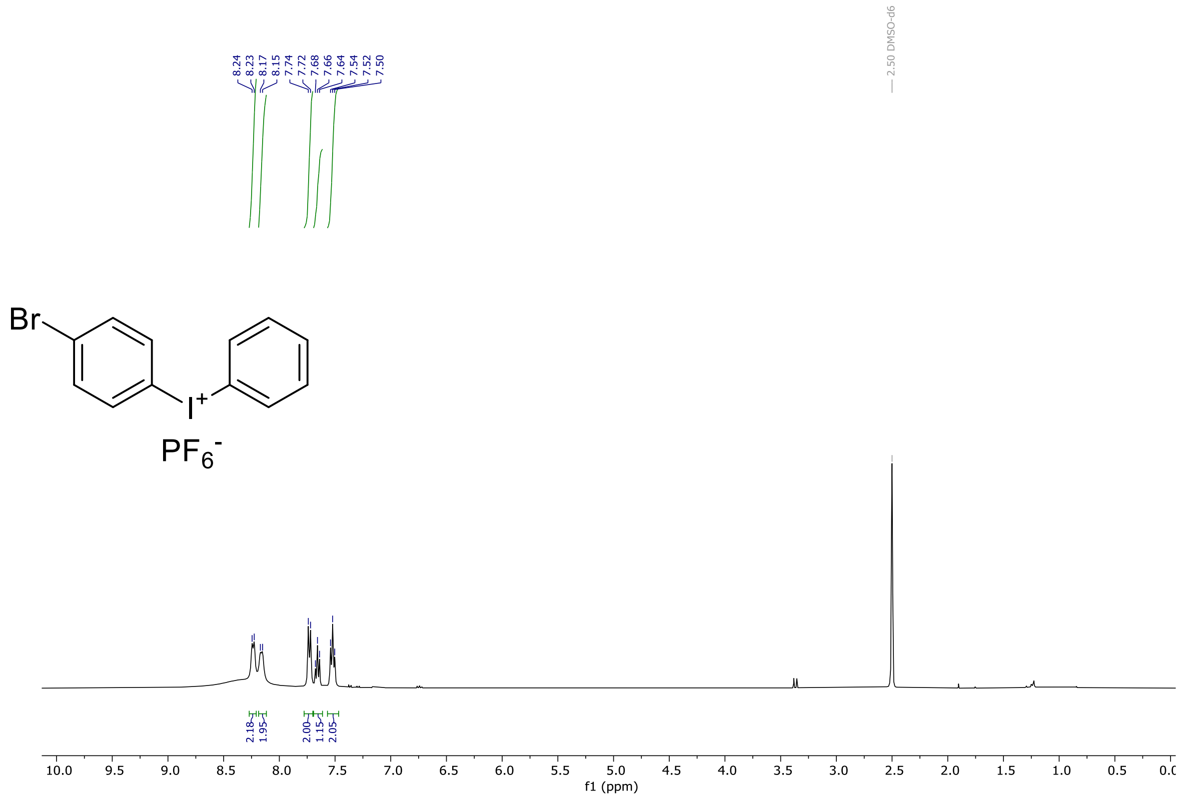

(4-Bromophenyl)(phenyl)iodonium hexafluorophosphate (**3c**-PF_6), $^{13}C\{^{1}H\}$ NMR spectrum (101 MHz, DMSO-d_6)

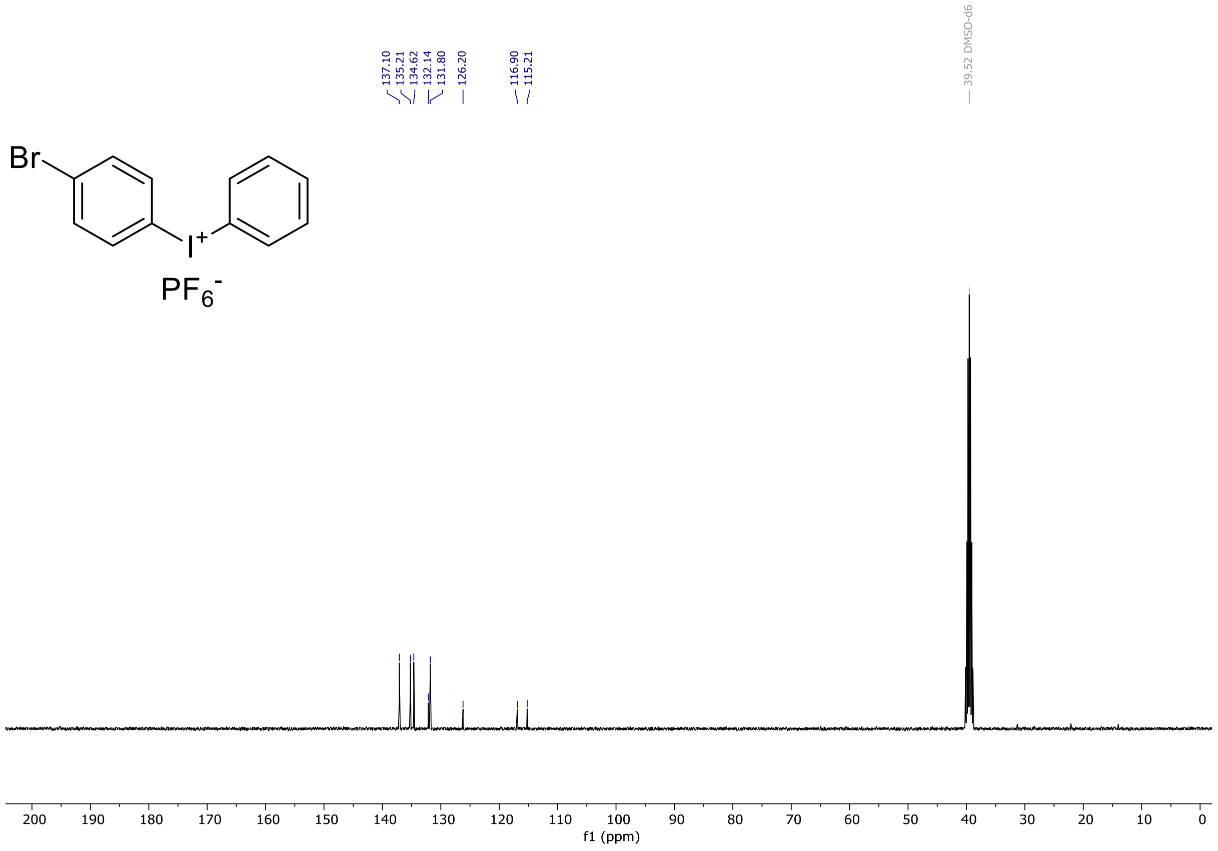

(4-Bromophenyl)(phenyl)iodonium hexafluorophosphate (**3c**-PF_6), ^{19}F NMR spectrum (376 MHz, DMSO-d_6)

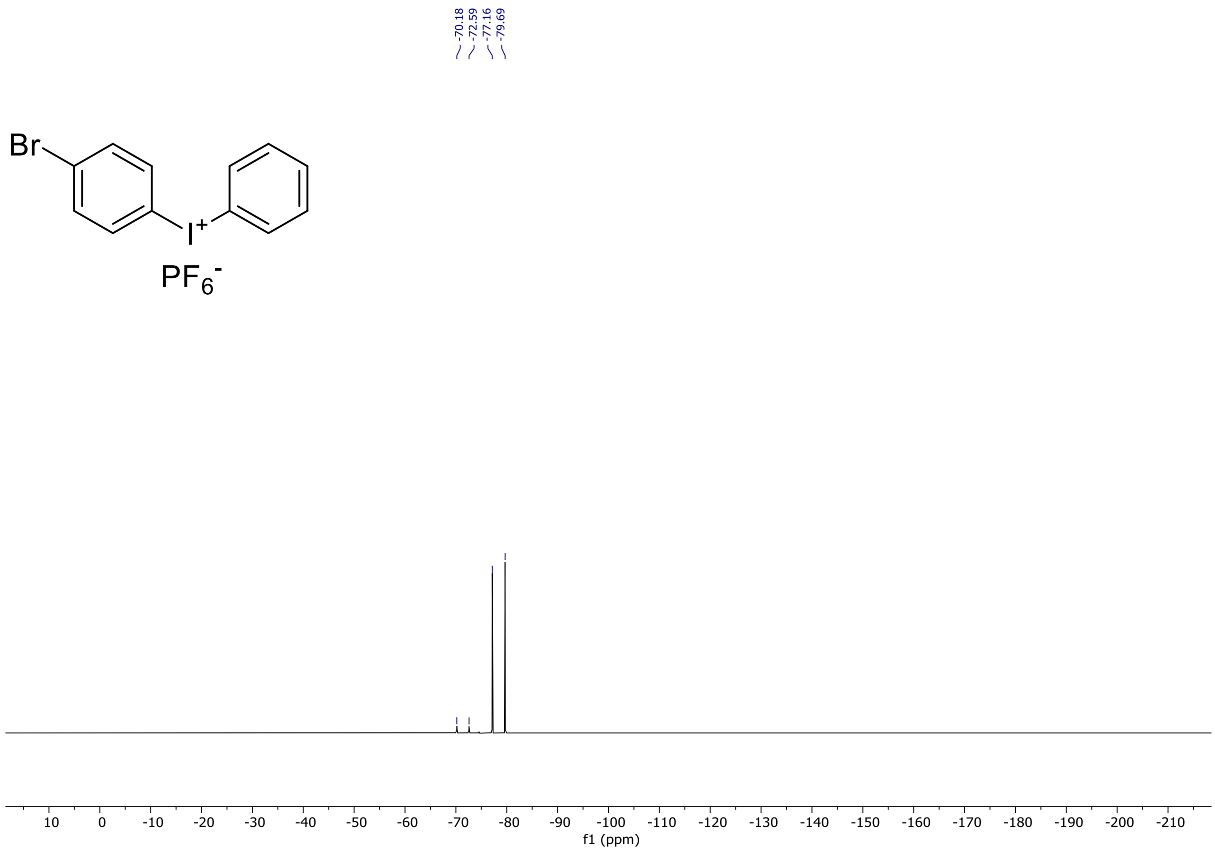

(4-Bromophenyl)(phenyl)iodonium hexafluorophosphate (**3c**-PF_6), ^{31}P NMR spectrum (162 MHz, DMSO-d_6)

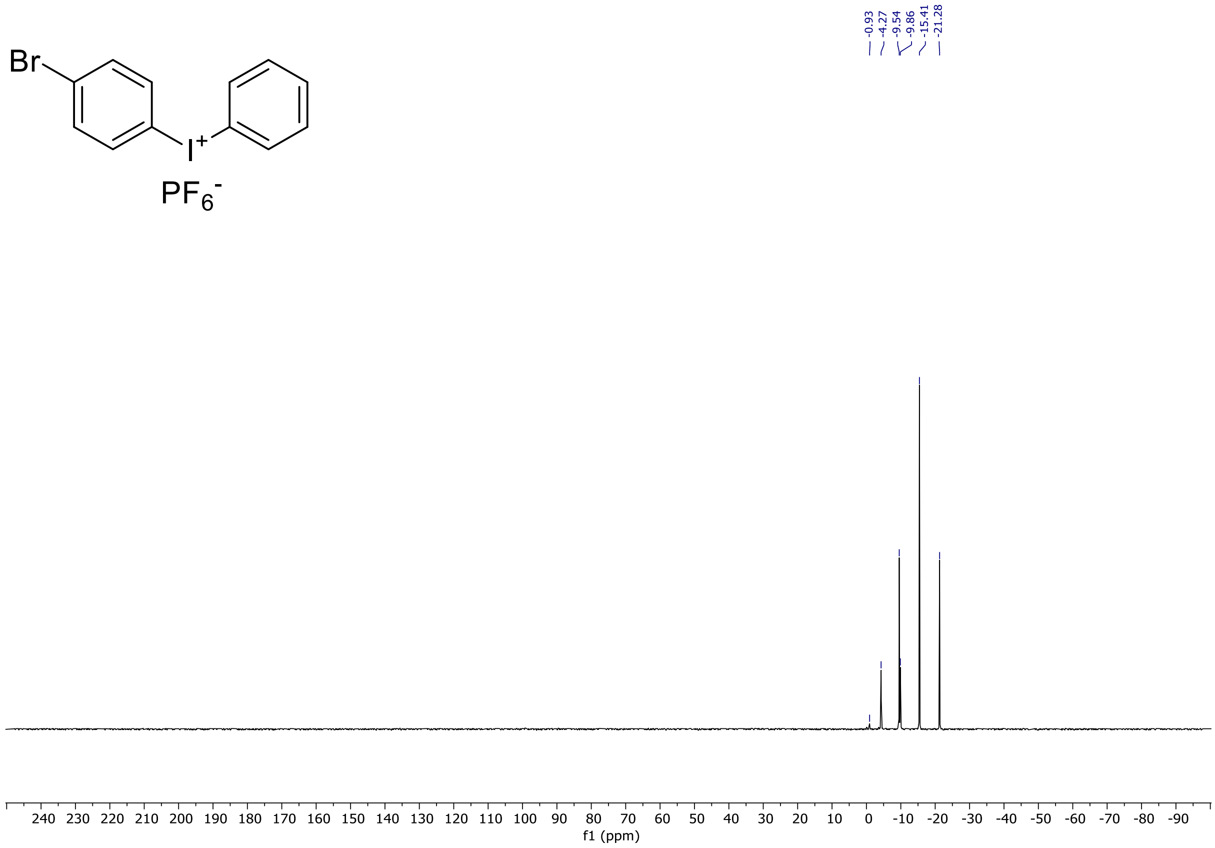

(4-Bromophenyl)(phenyl)iodonium bis((trifluoromethyl)sulfonyl)amide (**3c**-Tf_2N), ^{1}H NMR spectrum (400 MHz, CD_3CN)

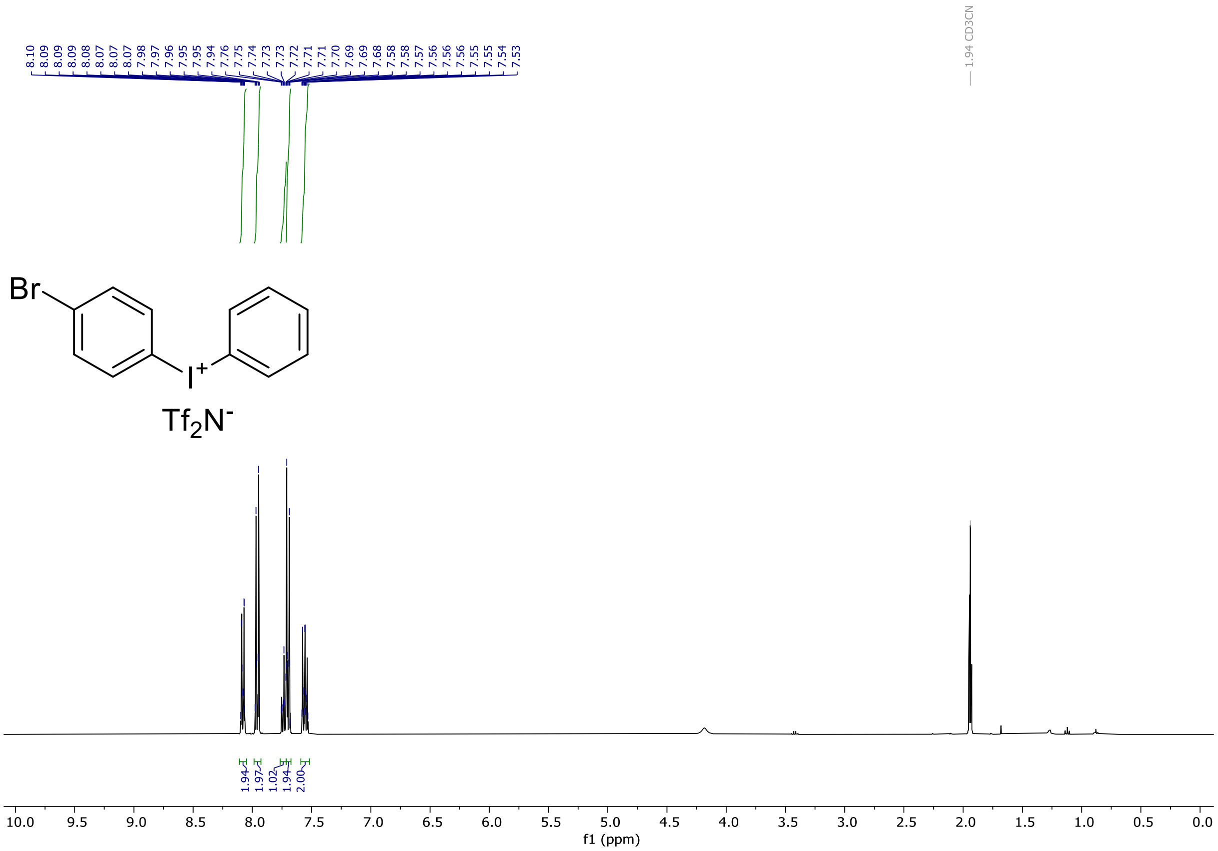

(4-Bromophenyl)(phenyl)iodonium bis((trifluoromethyl)sulfonyl)amide (**3c**-Tf_2N), $^{13}C\{^{1}H\}$ NMR spectrum (101 MHz, CD_3CN)

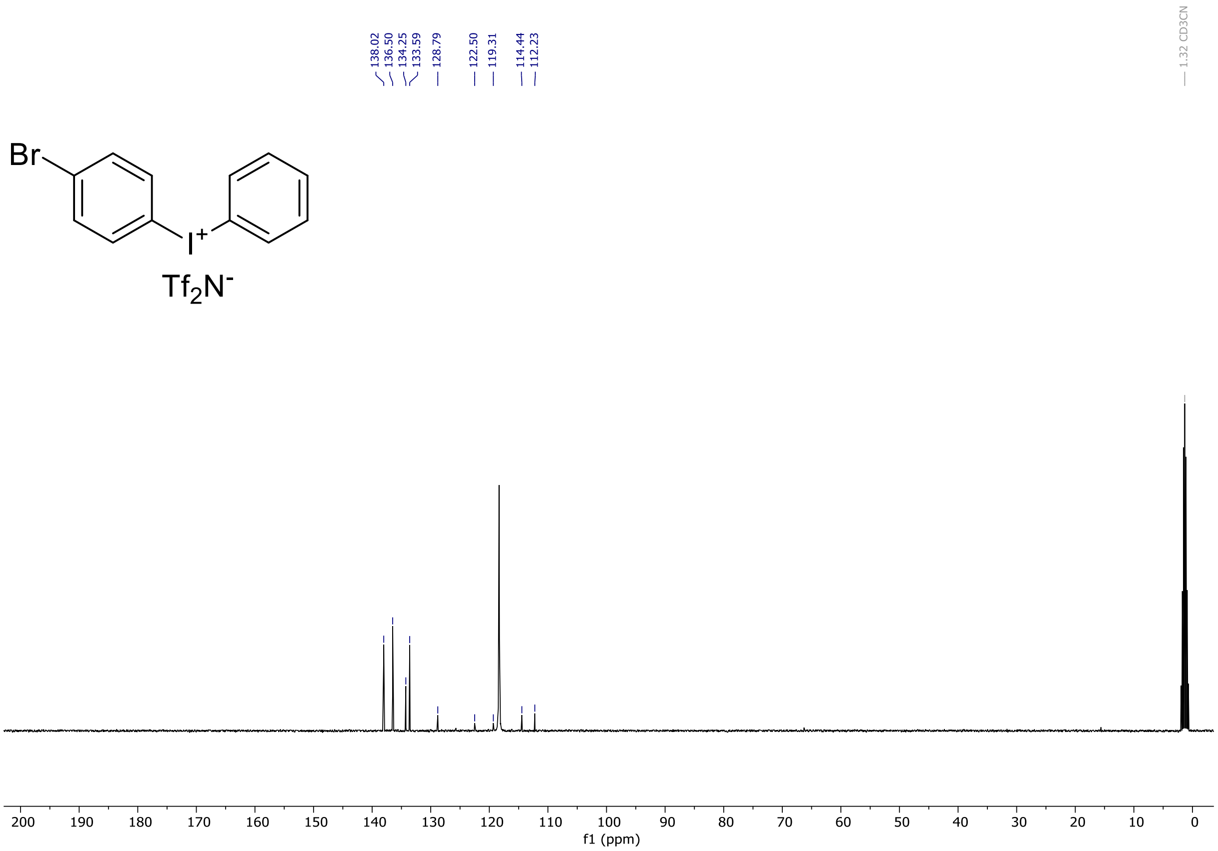

(4-Bromophenyl)(phenyl)iodonium bis((trifluoromethyl)sulfonyl)amide (**3c**-Tf_2N), ^{19}F NMR spectrum (376 MHz, CD_3CN)

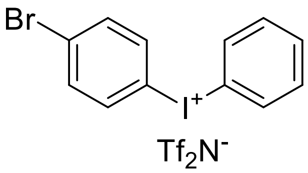

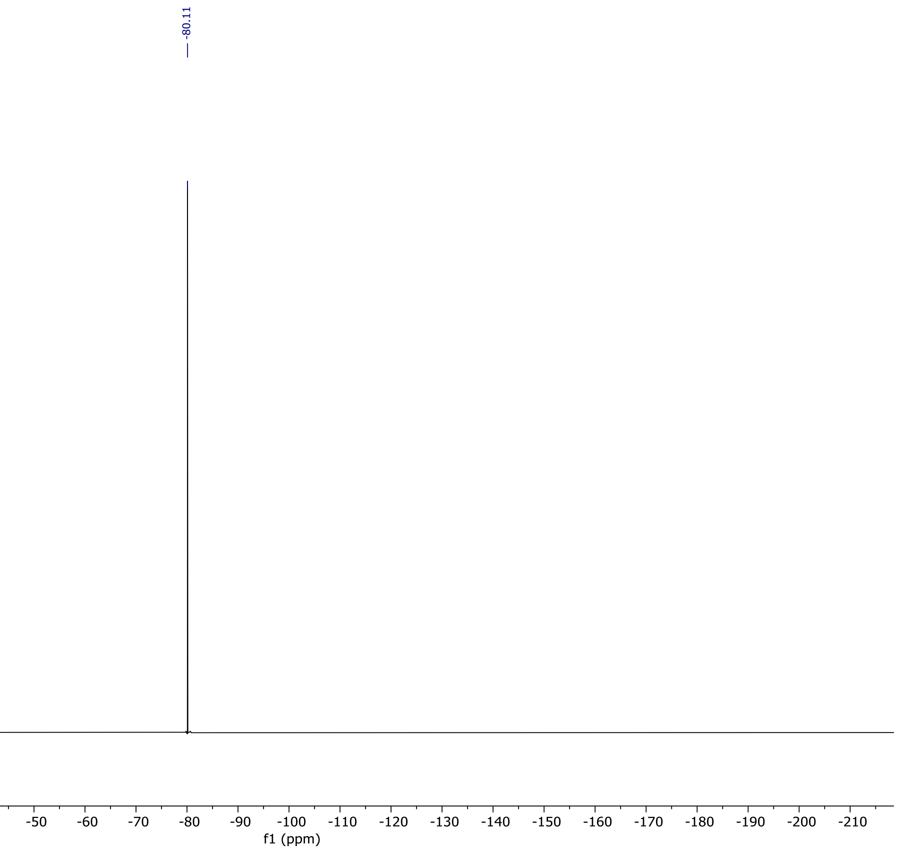

(4-Chlorophenyl)(phenyl)iodonium perchlorate (**3d**-ClO_4), ^{1}H NMR spectrum (400 MHz, CD_3CN)

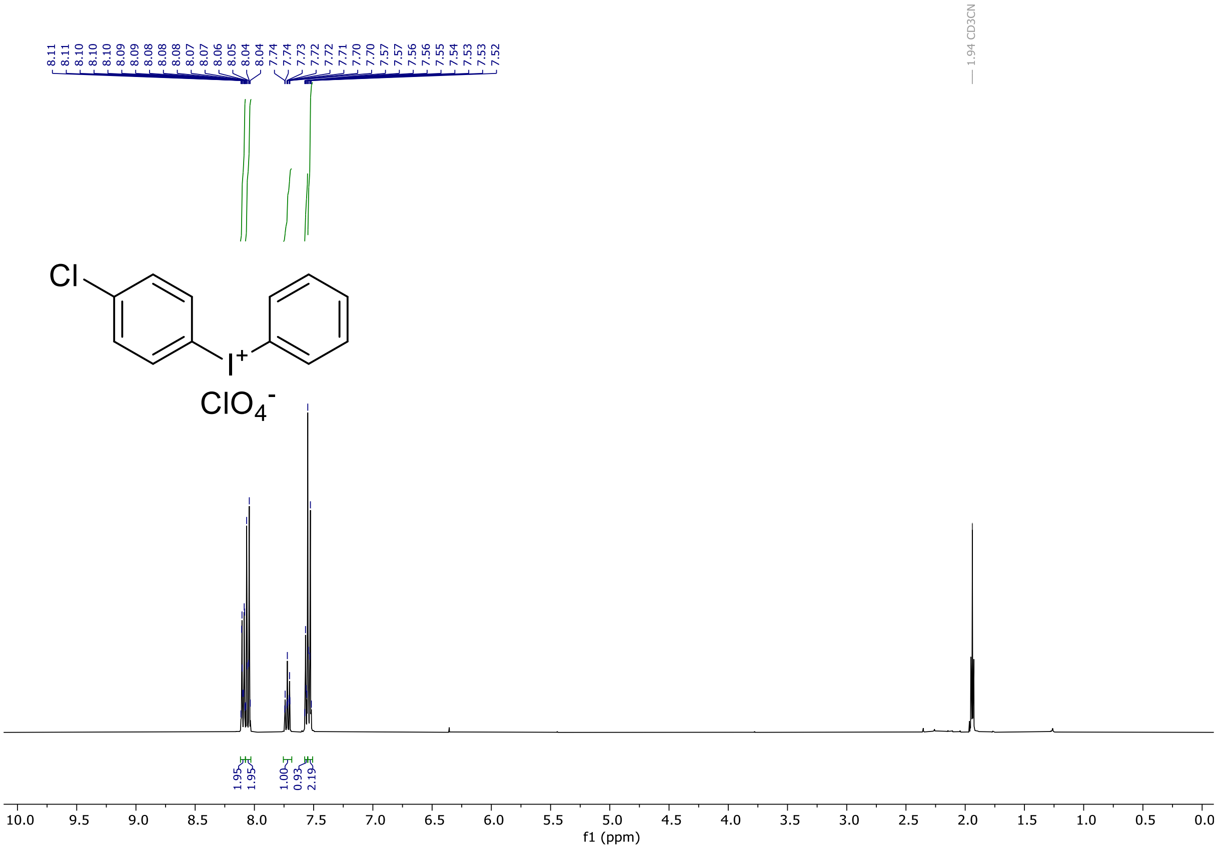

(4-Chlorophenyl)(phenyl)iodonium perchlorate (**3d**-ClO_4), $^{13}C\{^{1}H\}$ NMR spectrum (101 MHz, CD_3CN)

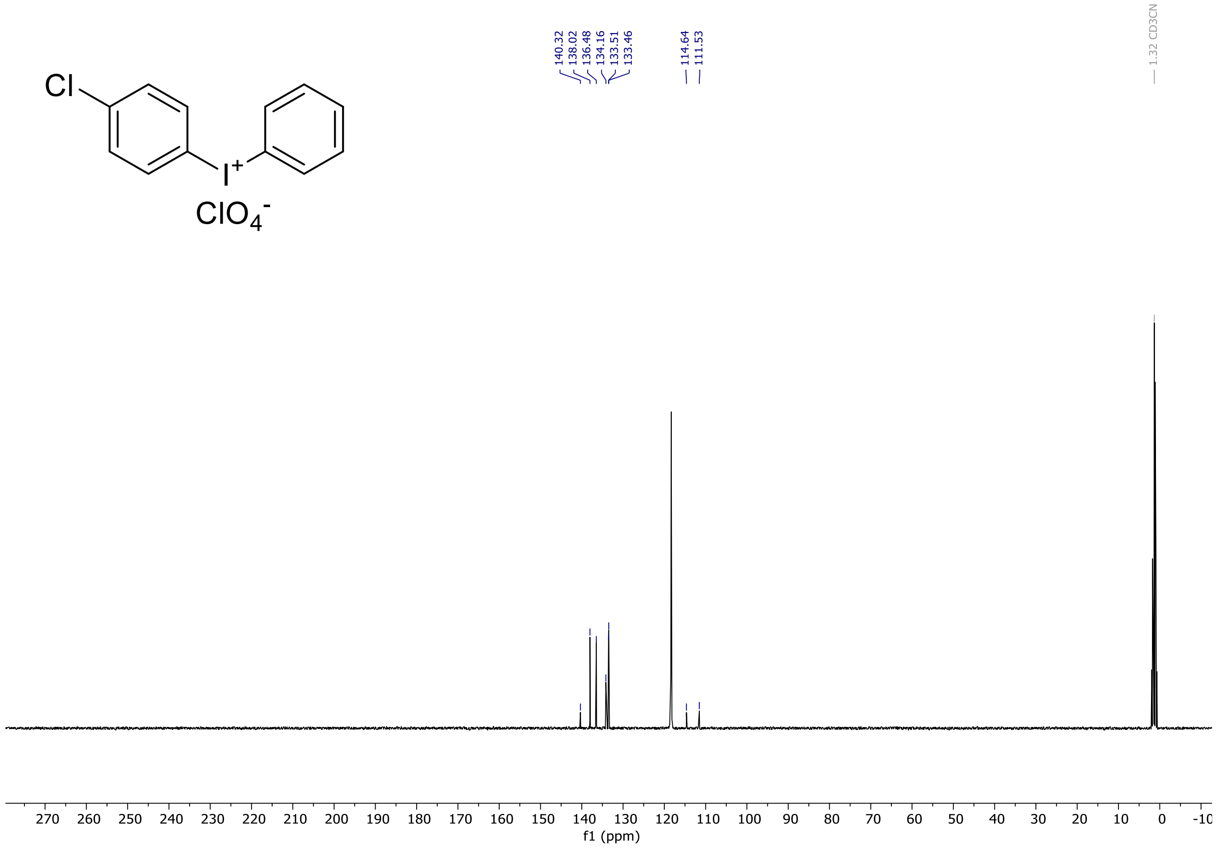

(4-Fluorophenyl)(phenyl)iodonium perchlorate (**3e**-ClO_4), ^{1}H NMR spectrum (400 MHz, CD_3CN)

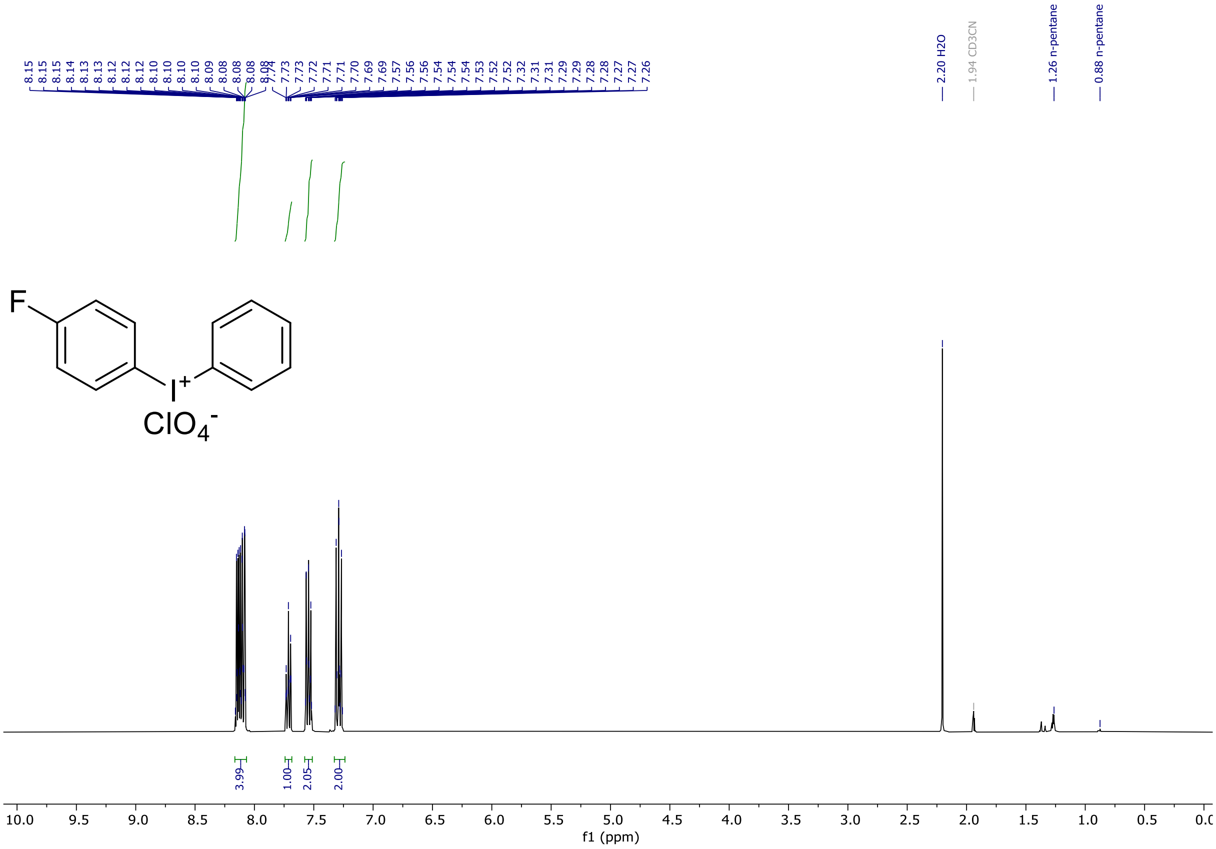

(4-Fluorophenyl)(phenyl)iodonium perchlorate (**3e**-ClO_4), ^{13}C{^{1}H} NMR spectrum (101 MHz, CD_3CN)

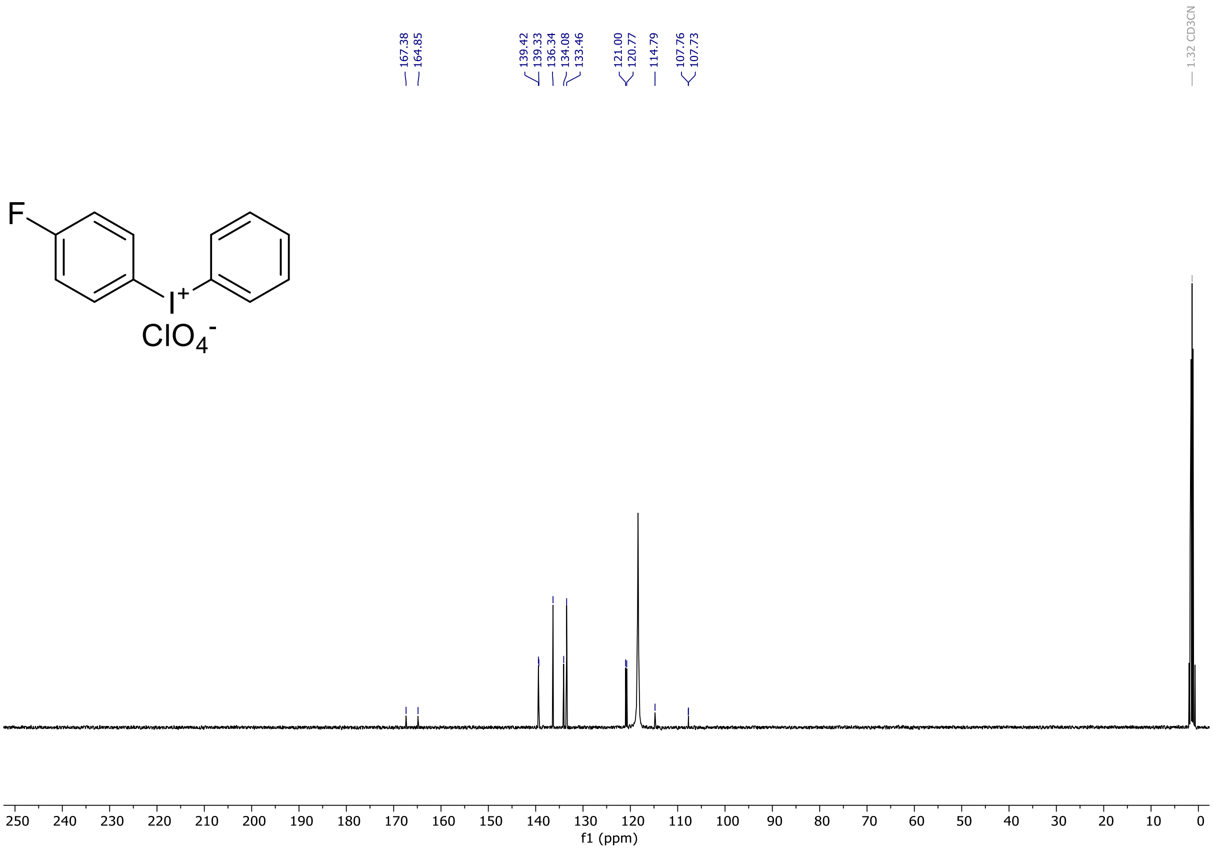

(4-Fluorophenyl)(phenyl)iodonium perchlorate (**3e**-ClO_4), ^{19}F NMR spectrum (376 MHz, CD_3CN)

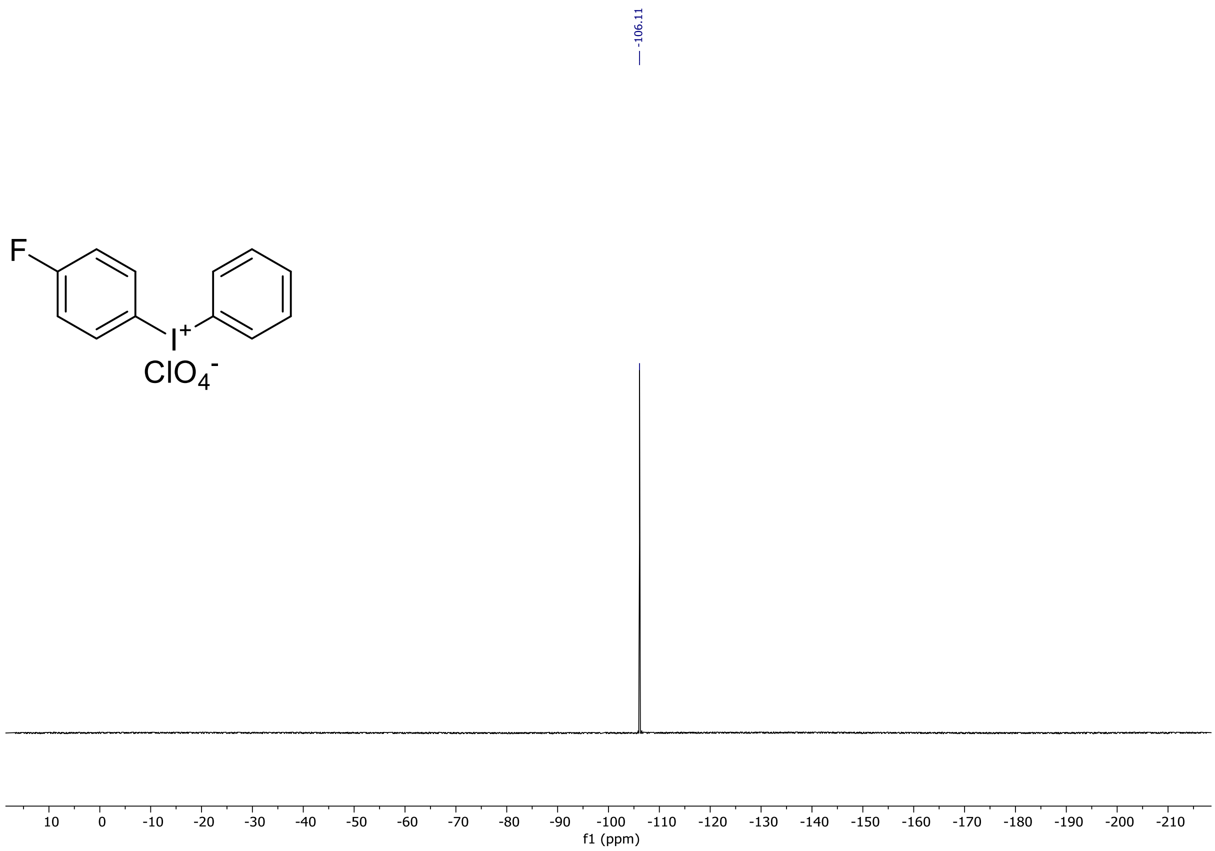

Phenyl(*p*-tolyl)iodonium perchlorate (**3f**-ClO_4), ^1H NMR spectrum (400 MHz, CD_3CN)

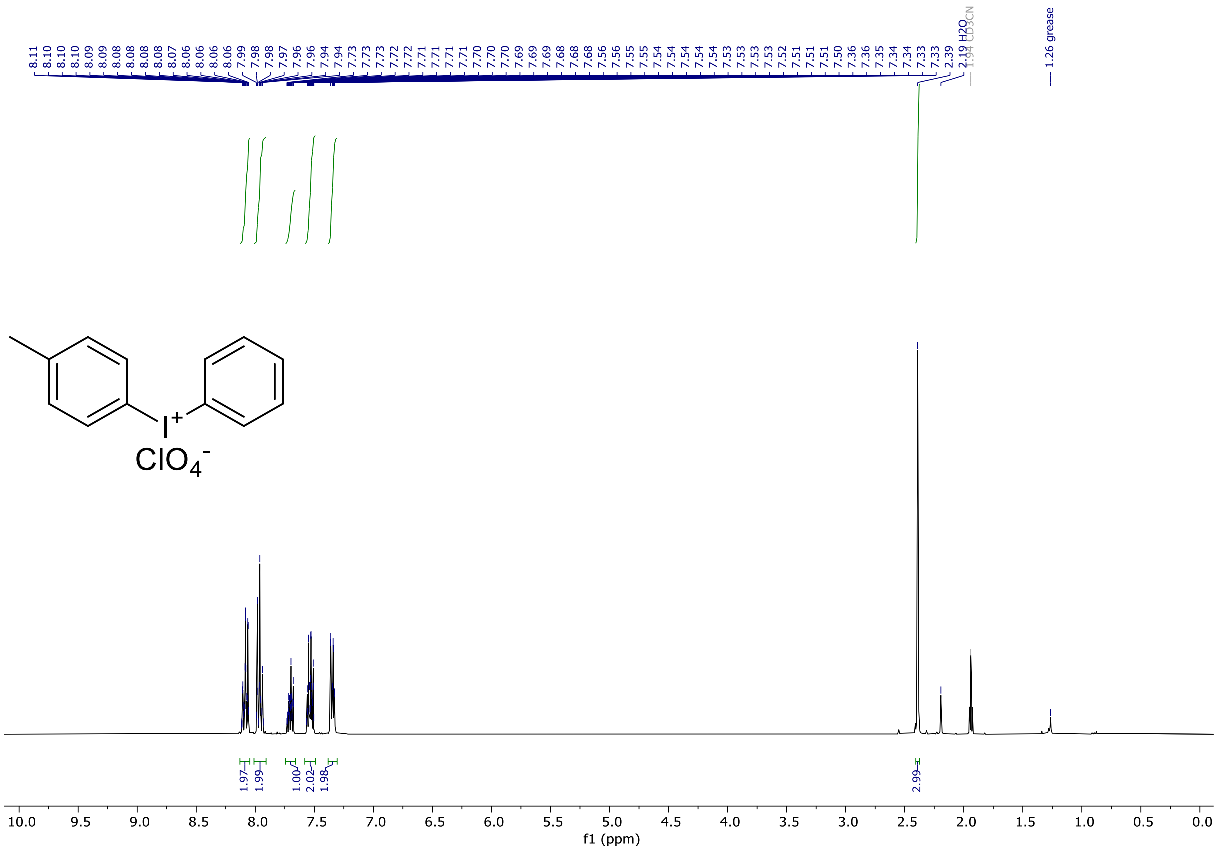

Phenyl(*p*-tolyl)iodonium perchlorate (**3f**-ClO_4), $^{13}C\{^1H\}$ NMR spectrum (101 MHz, CD_3CN)

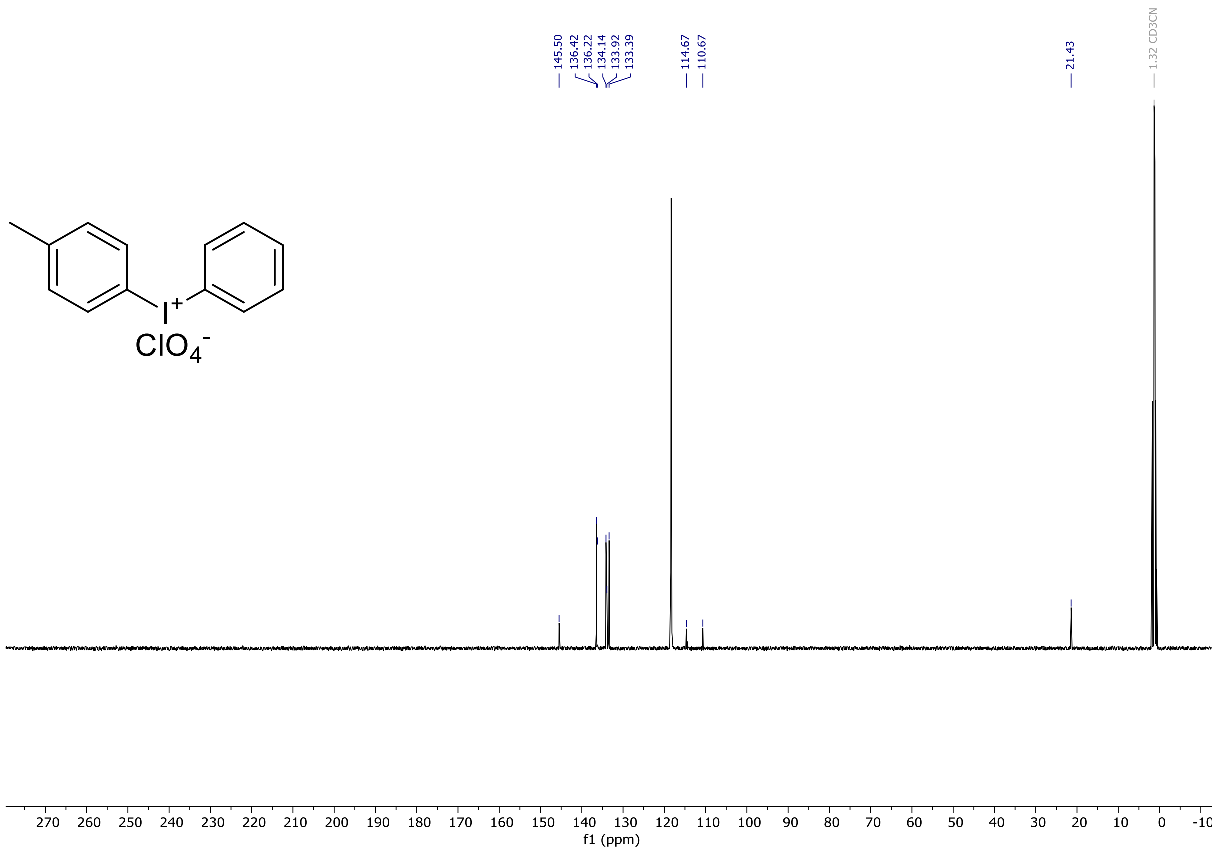

(4-(*tert*-Butyl)phenyl)(phenyl)iodonium perchlorate (**3g**-ClO_4), ^1H NMR spectrum (400 MHz, CD_3CN)

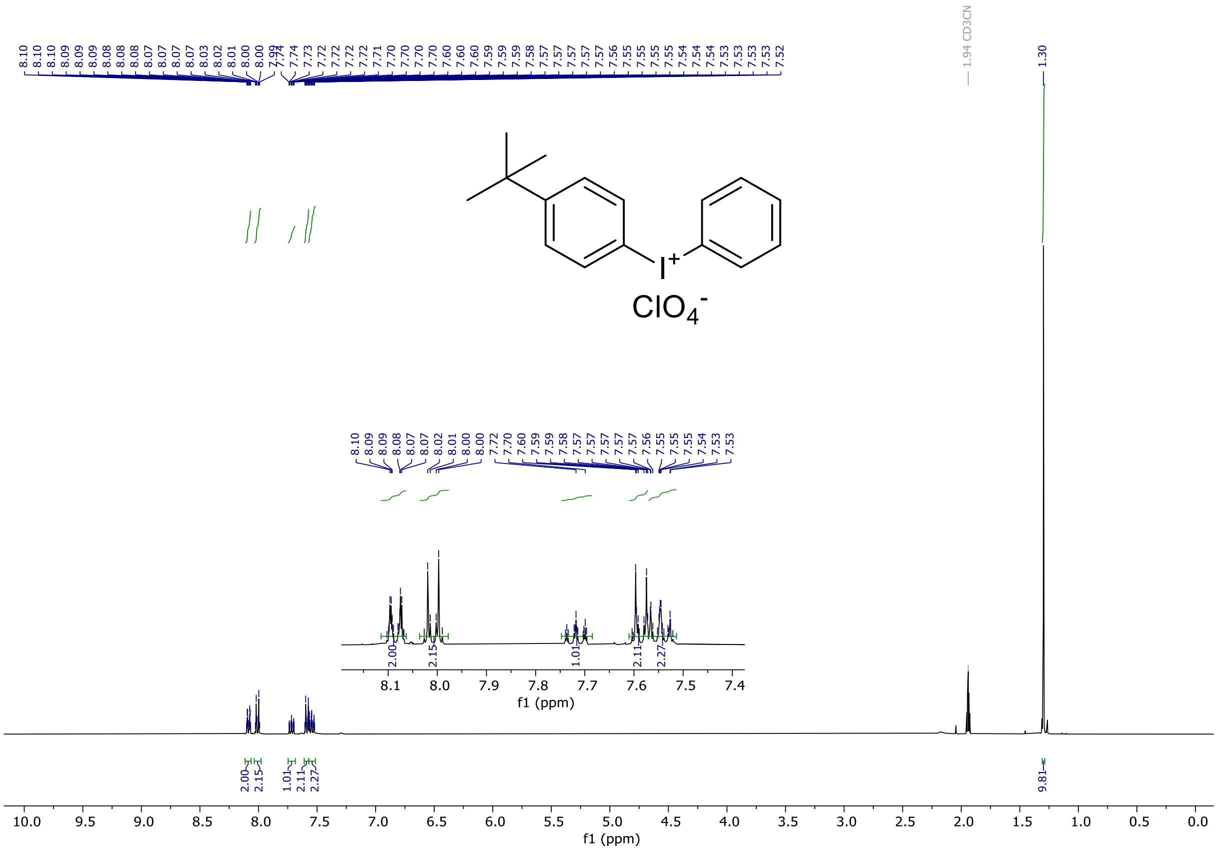

(4-(*tert*-Butyl)phenyl)(phenyl)iodonium perchlorate (**3g**-ClO_4), $^{13}C\{^1H\}$ NMR spectrum (101 MHz, CD_3CN)

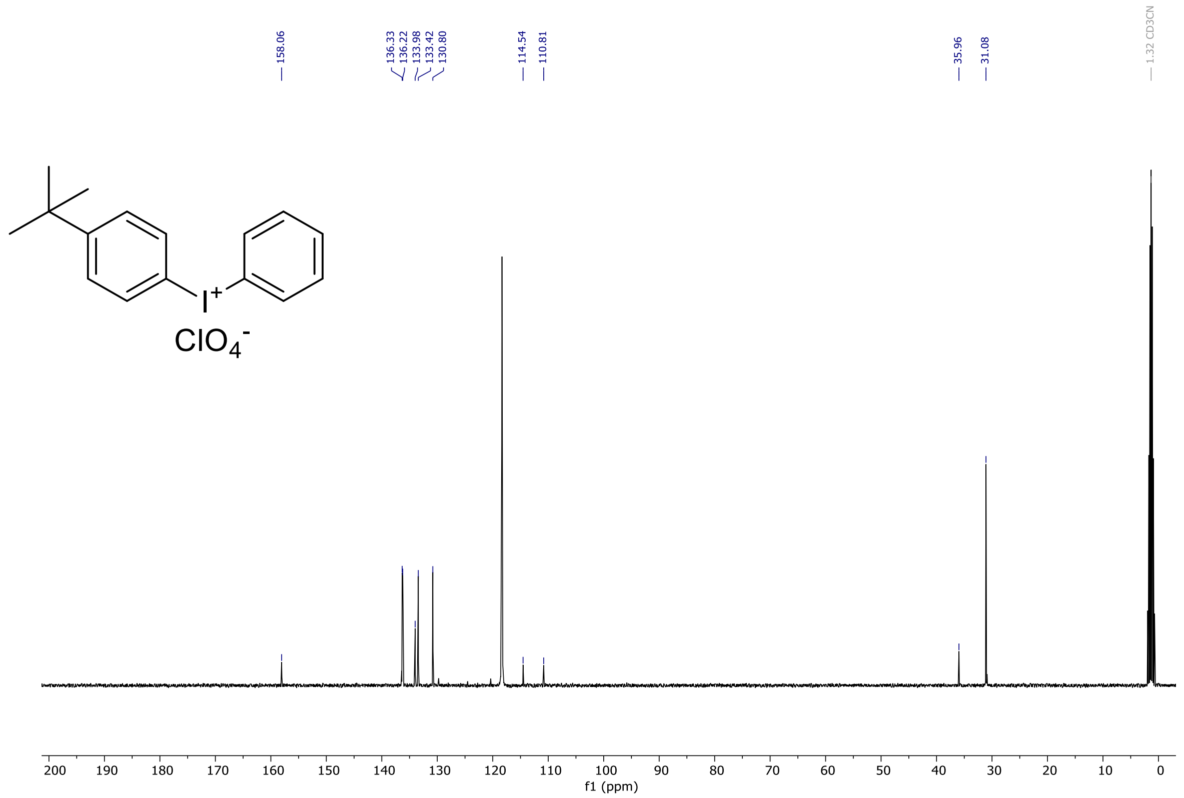

Phenyl(4-(trifluoromethyl)phenyl)iodonium perchlorate (**3h**-ClO_4), ^{1}H NMR spectrum (400 MHz, CD_3CN)

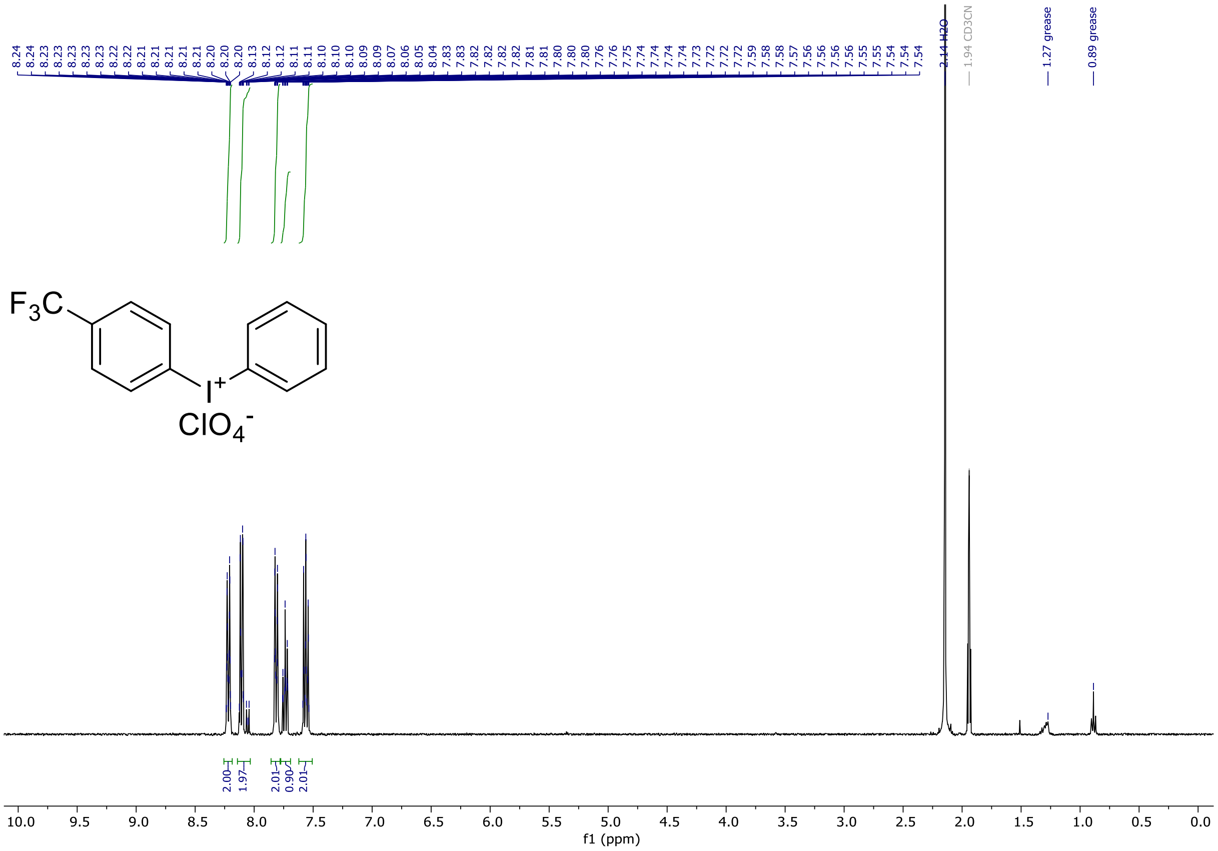

Phenyl(4-(trifluoromethyl)phenyl)iodonium perchlorate (**3h**-ClO_4), $^{13}C\{^{1}H\}$ NMR spectrum (101 MHz, CD_3CN)

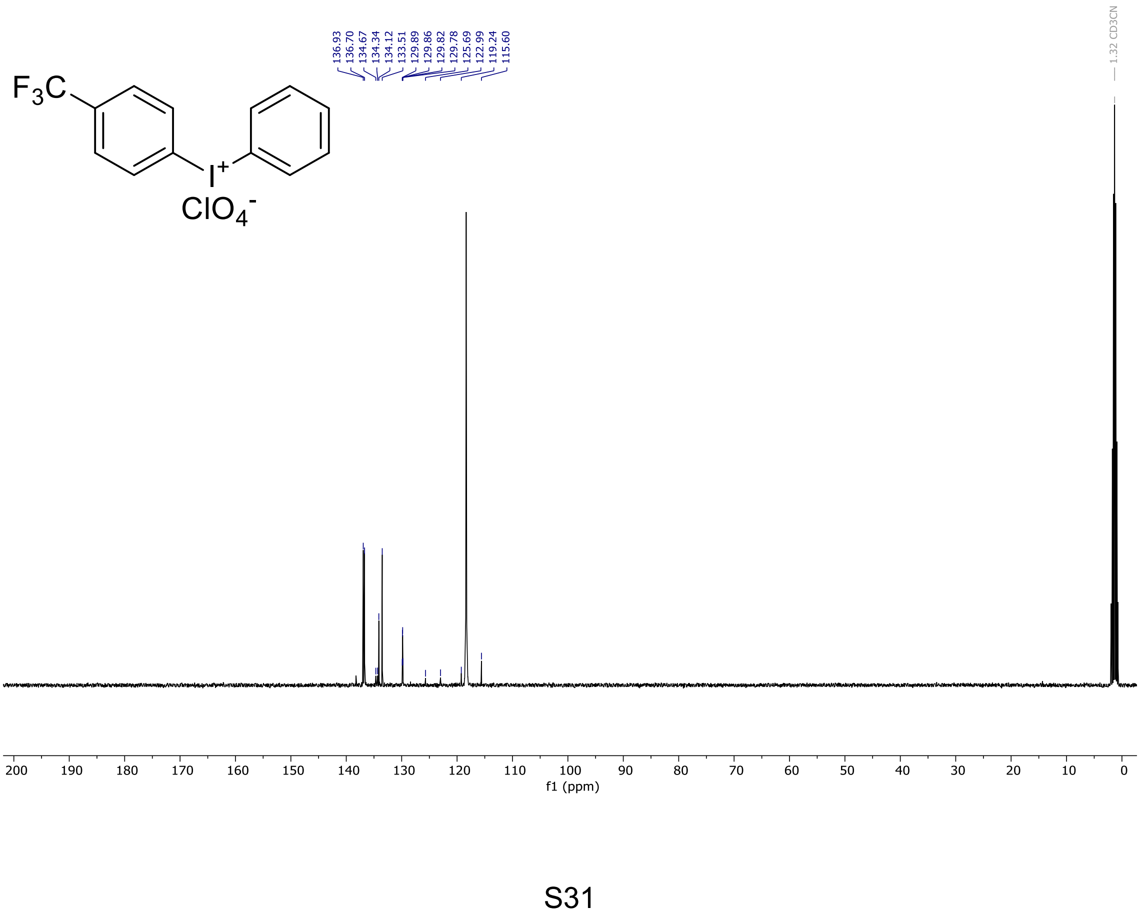

Phenyl(4-(trifluoromethyl)phenyl)iodonium perchlorate (**3h**-ClO_4), ^{19}F NMR spectrum (376 MHz, CD_3CN)

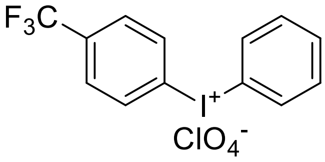

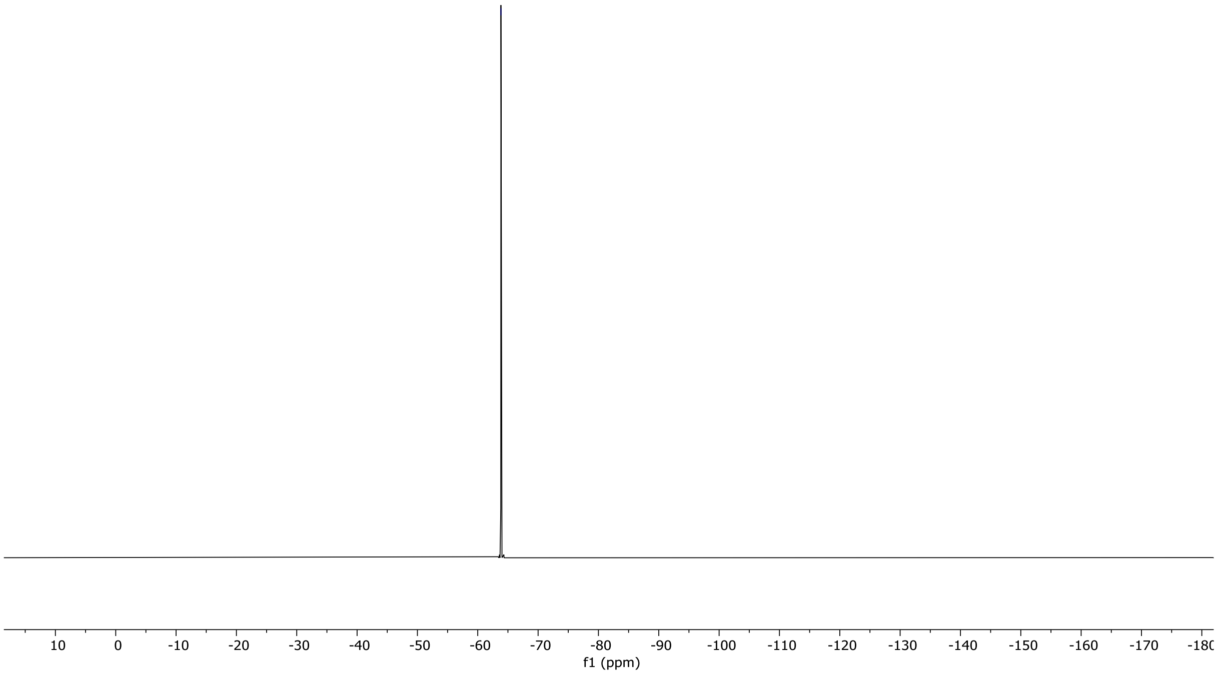

(4-Cyanophenyl)(phenyl)iodonium perchlorate (**3i**-ClO_4), ^1H NMR spectrum (400 MHz, CD_3OD)

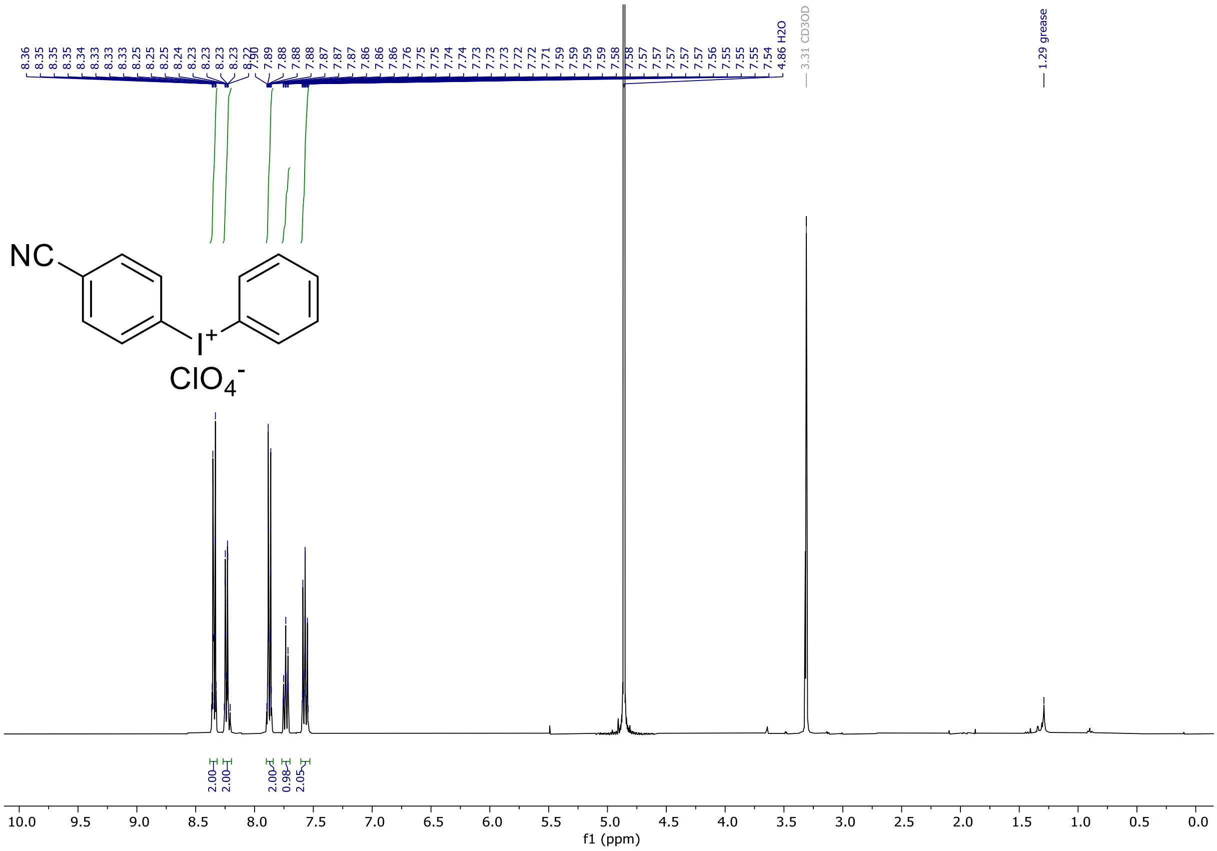

(4-Cyanophenyl)(phenyl)iodonium perchlorate (**3i**-ClO_4), $^{13}C\{^1H\}$ NMR spectrum (101 MHz, CD_3OD)

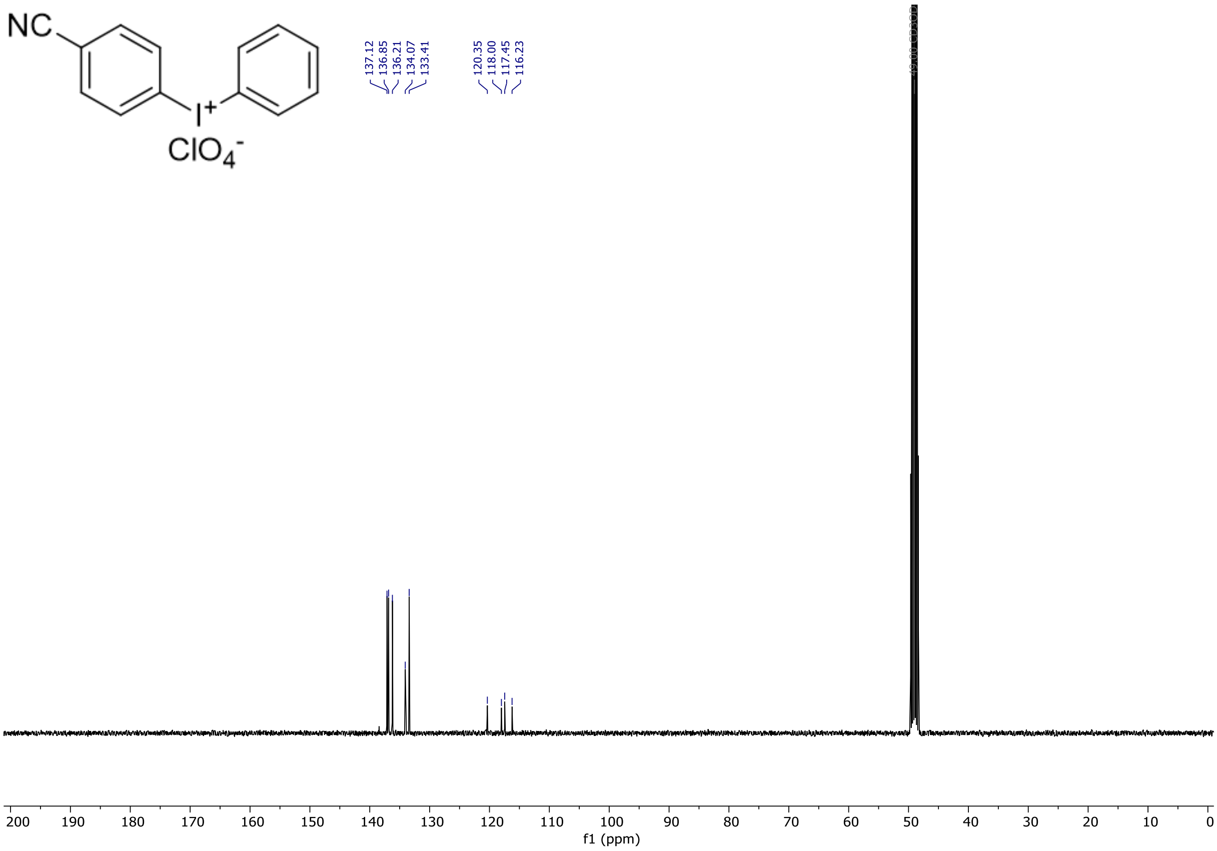

(4-Bromophenyl)(*p*-tolyl)iodonium perchlorate (**3j**-ClO_4), ^{1}H NMR spectrum (400 MHz, CD_3CN)

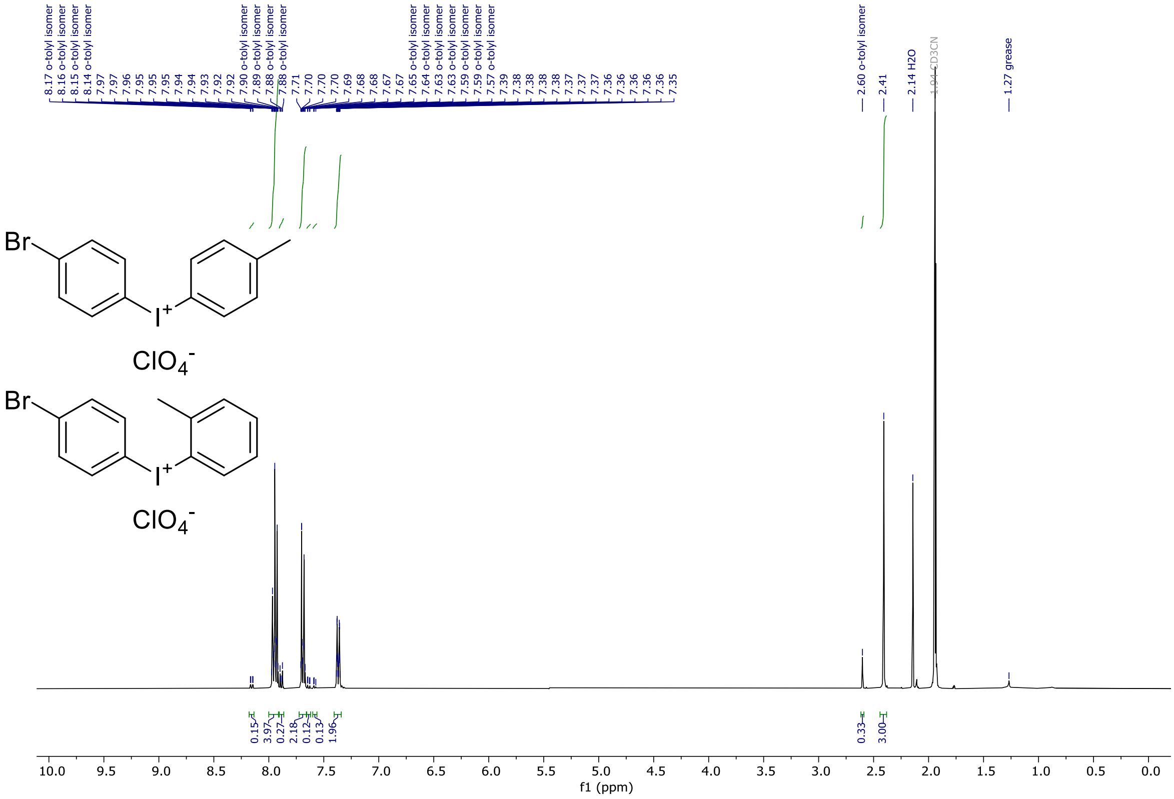

(4-Bromophenyl)(*p*-tolyl)iodonium perchlorate (**3j**-ClO_4), $^{13}C\{^{1}H\}$ NMR spectrum (101 MHz, CD_3CN)

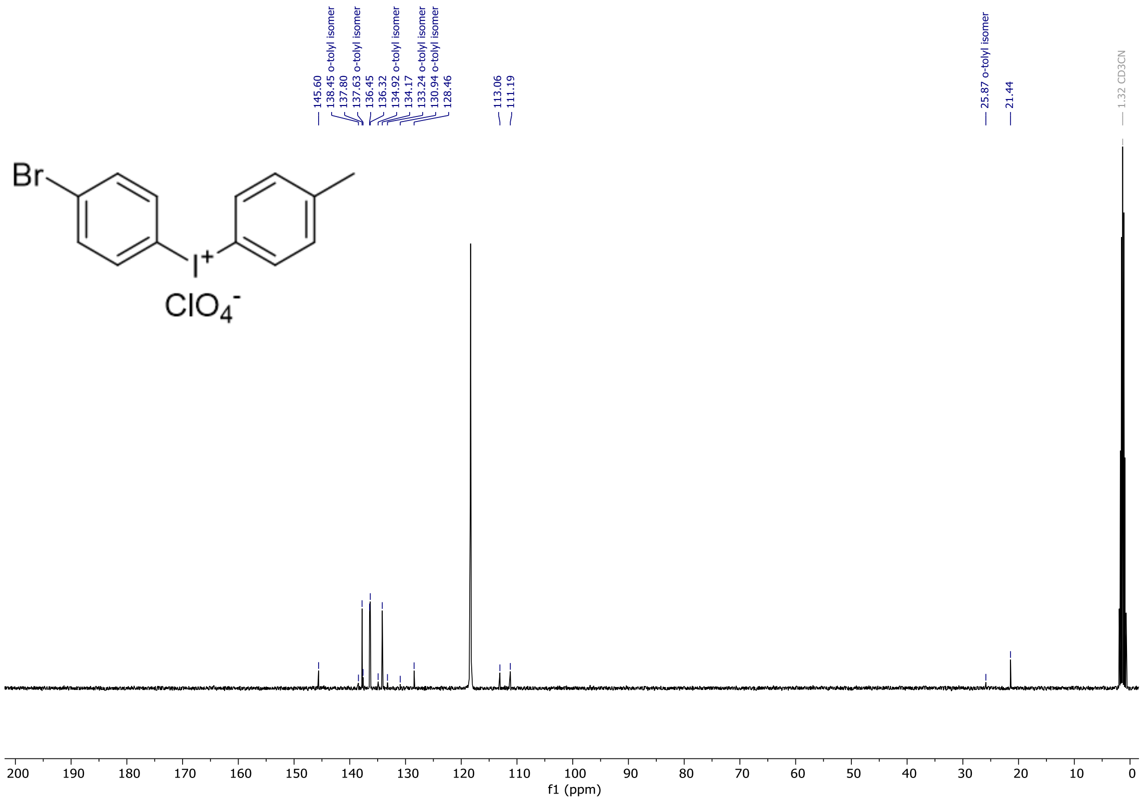

(4-Bromophenyl)(mesityl)iodonium perchlorate (**3k**-ClO_4), ^{1}H NMR spectrum (400 MHz, CD_3CN)

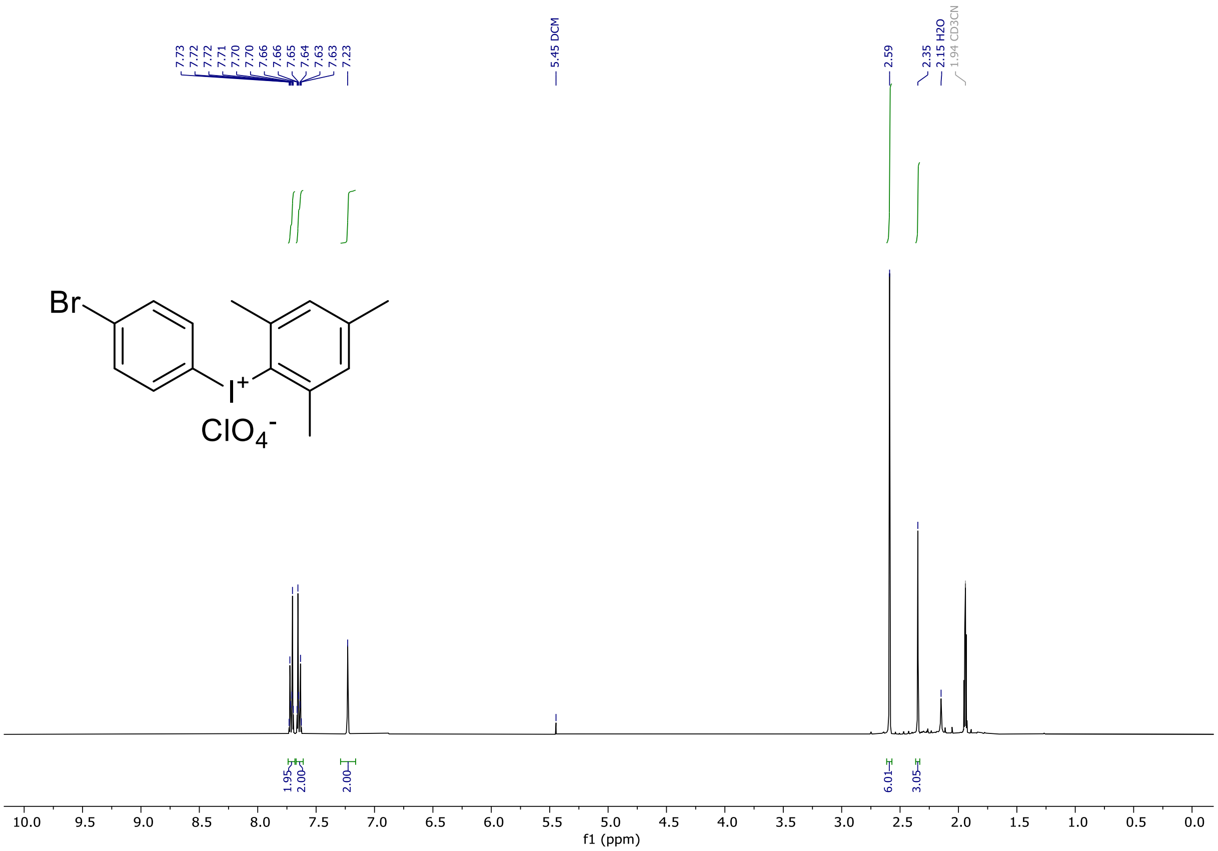

(4-Bromophenyl)(mesityl)iodonium perchlorate (**3k**-ClO_4), $^{13}C\{^{1}H\}$ NMR spectrum (101 MHz, CD_3CN)

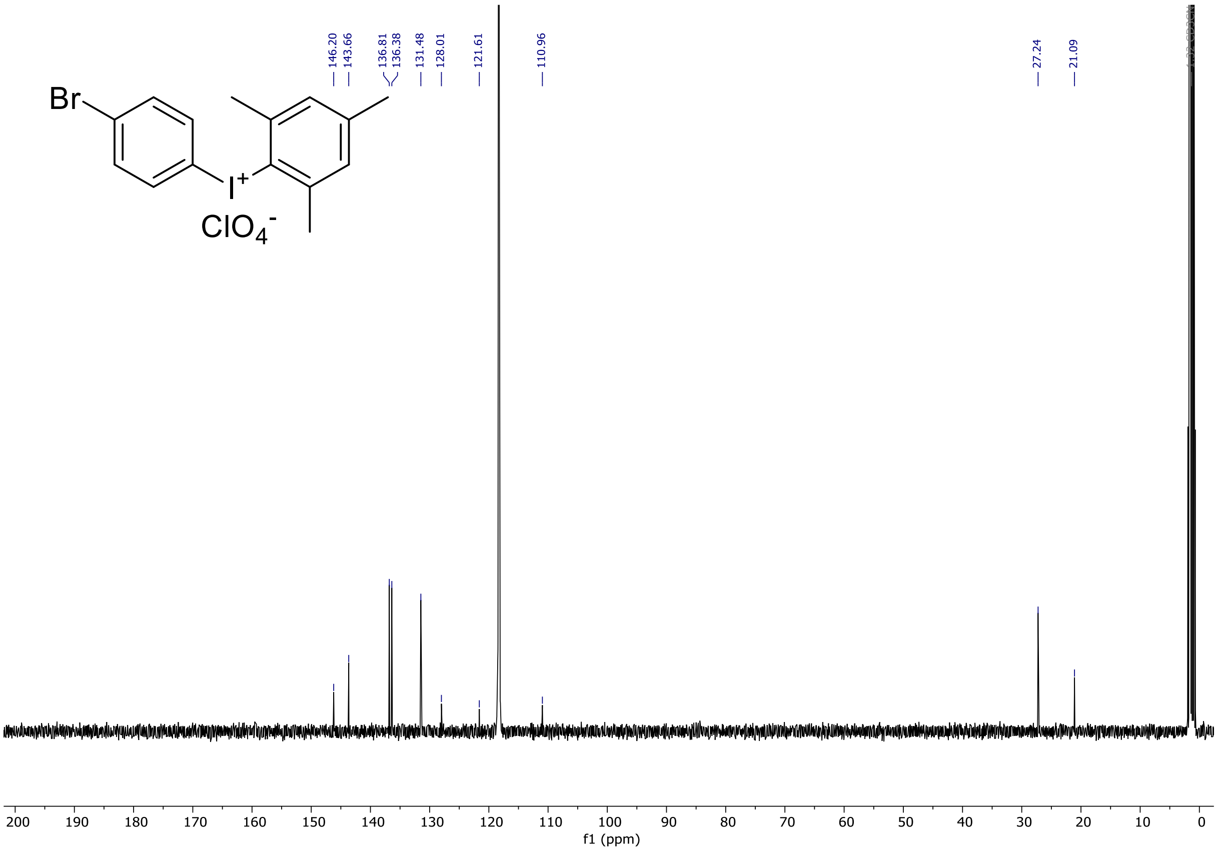

(4-Bromophenyl)(2,5-dimethylphenyl)iodonium perchlorate (**3l**-ClO_4), ^1H NMR spectrum (400 MHz, DMSO-d_6)

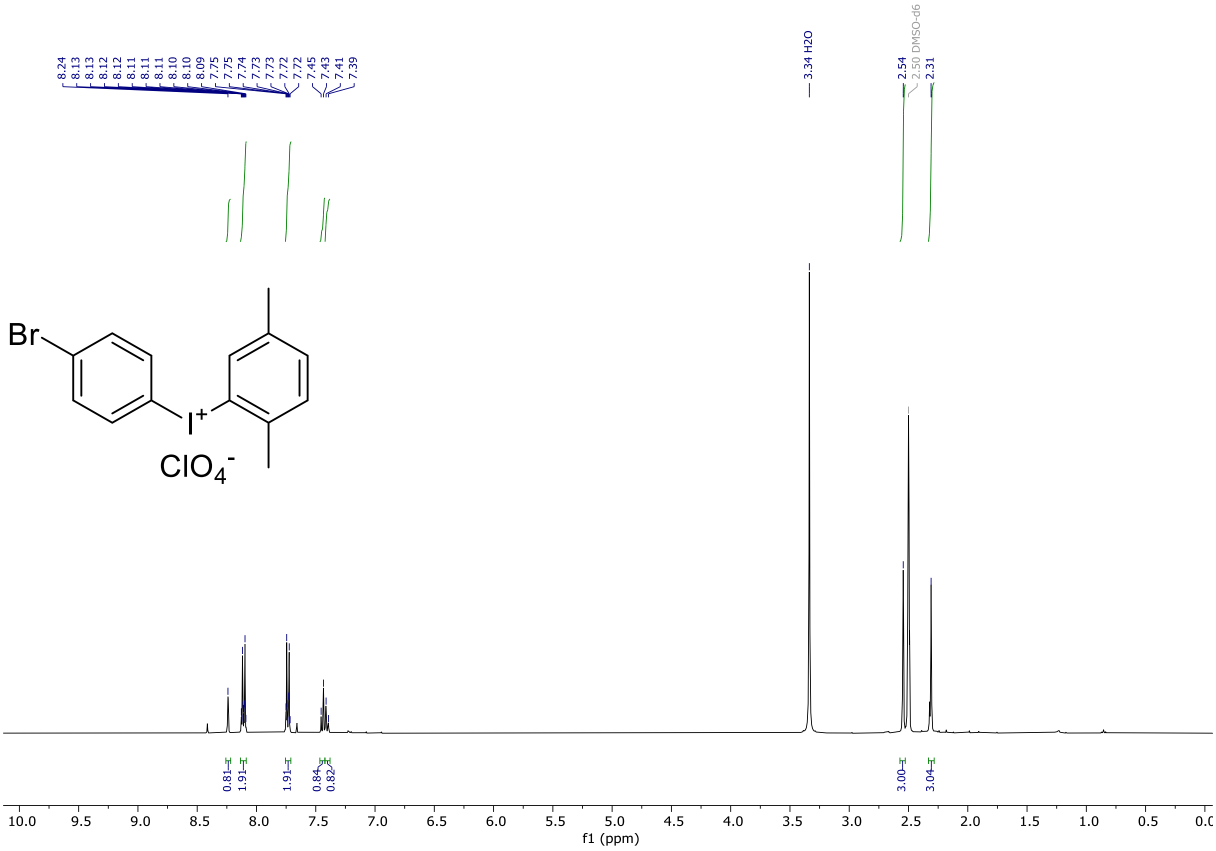

(4-Bromophenyl)(2,5-dimethylphenyl)iodonium perchlorate (**3l**-ClO_4), $^{13}C\{^1H\}$ NMR spectrum (101 MHz, DMSO-d_6)

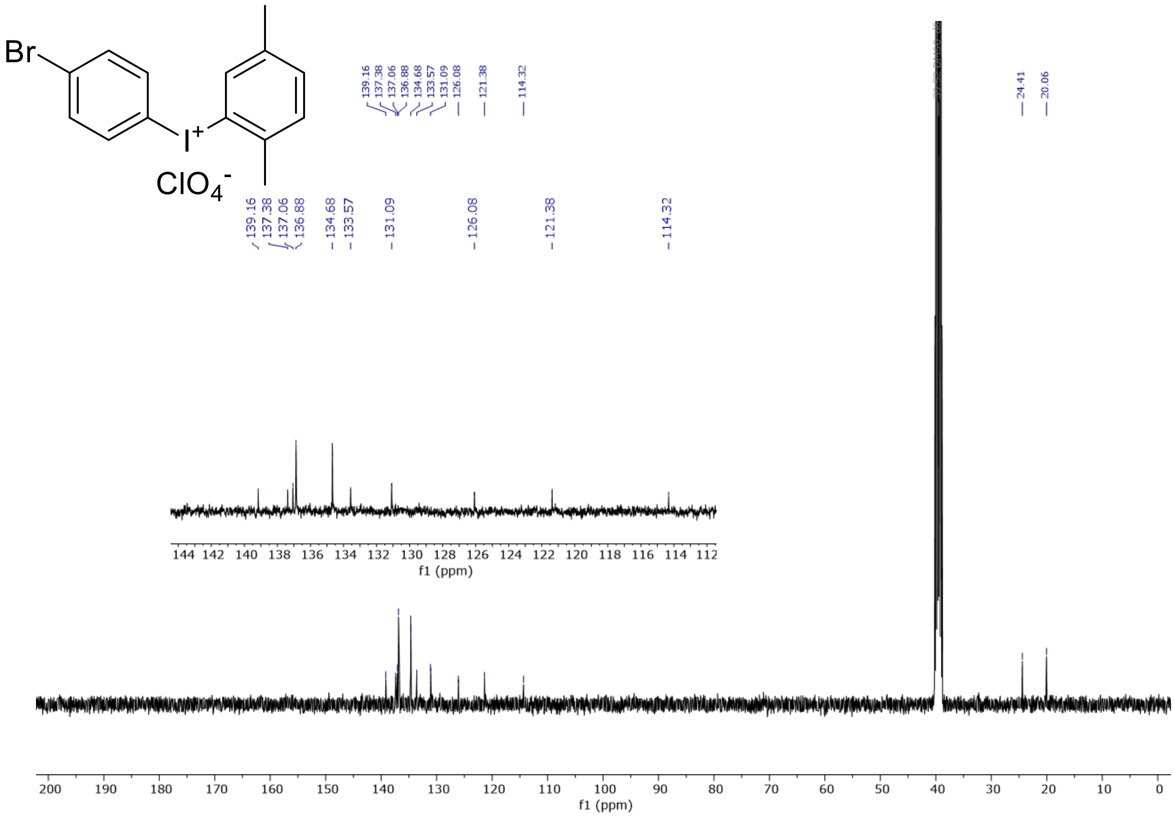

(4-Bromophenyl)(4-(*tert*-butyl)phenyl)iodonium perchlorate (**3m**-ClO_4), ^{1}H NMR spectrum (400 MHz, CD_3OD)

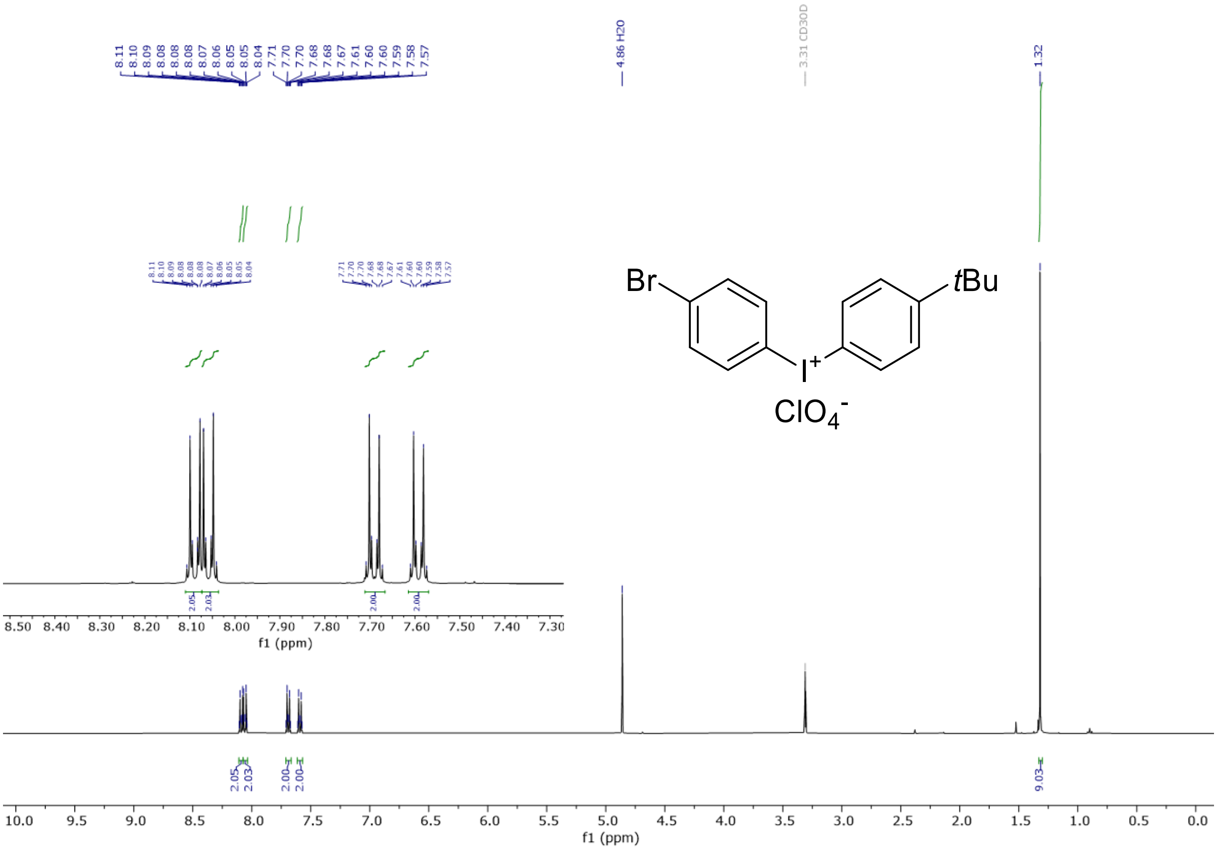

(4-Bromophenyl)(4-(*tert*-butyl)phenyl)iodonium perchlorate (**3m**-ClO_4), $^{13}C\{^{1}H\}$ NMR spectrum (101 MHz, CD_3OD)

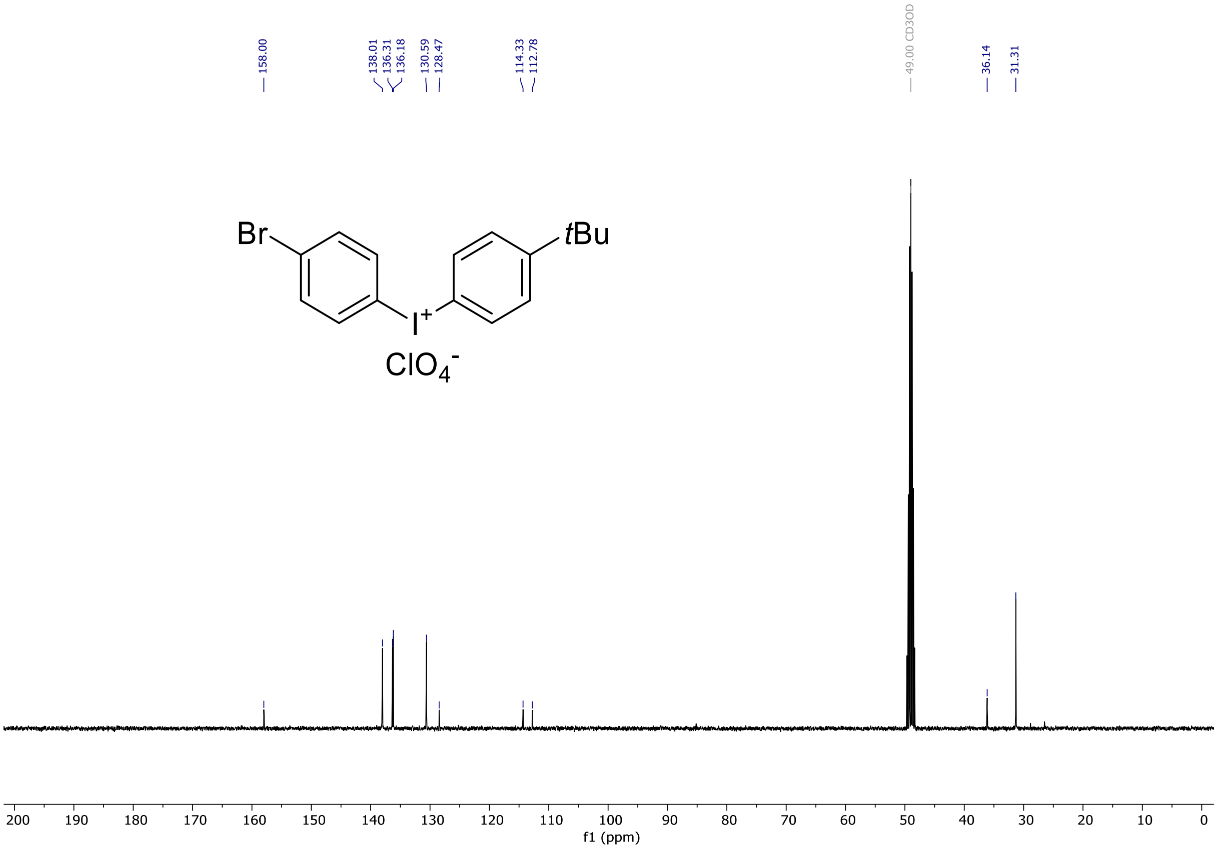

(4-Bromophenyl)(4-chlorophenyl)iodonium perchlorate (**3n**-ClO_4), ^1H NMR spectrum (400 MHz, DMSO-d_6)

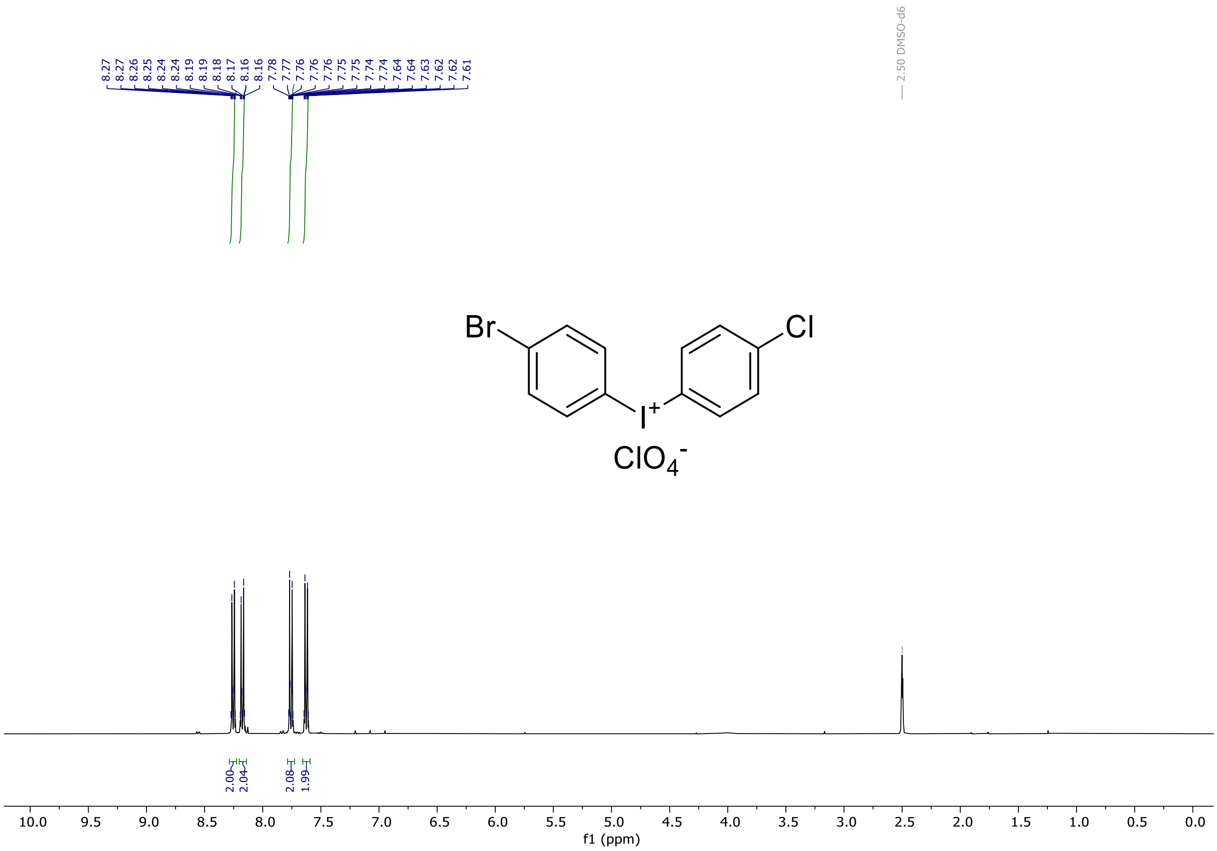

(4-Bromophenyl)(4-chlorophenyl)iodonium perchlorate (**3n**-ClO_4), $^{13}C\{^1H\}$ NMR spectrum (101 MHz, DMSO-d_6)

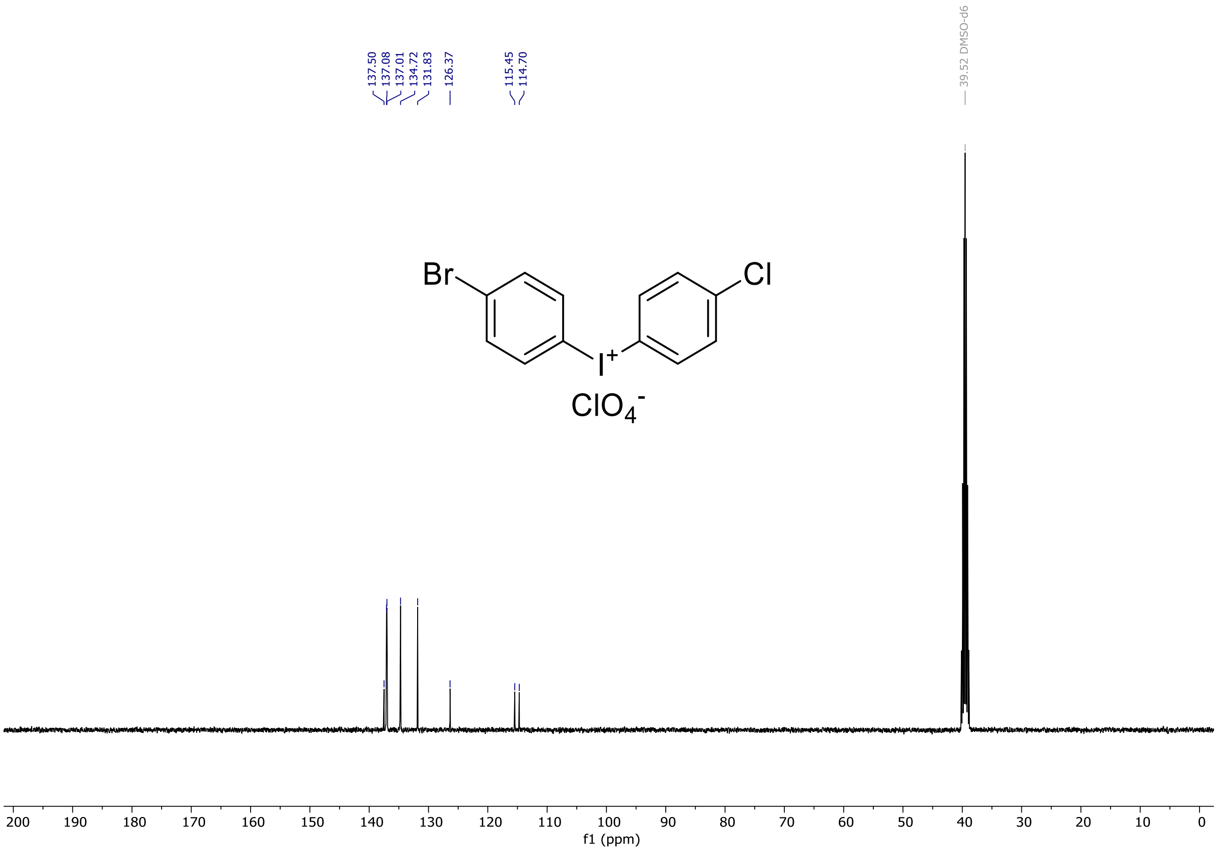

Bis(4-bromophenyl)iodonium perchlorate (**3o**-ClO_4), ^1H NMR spectrum (400 MHz, CD_3CN)

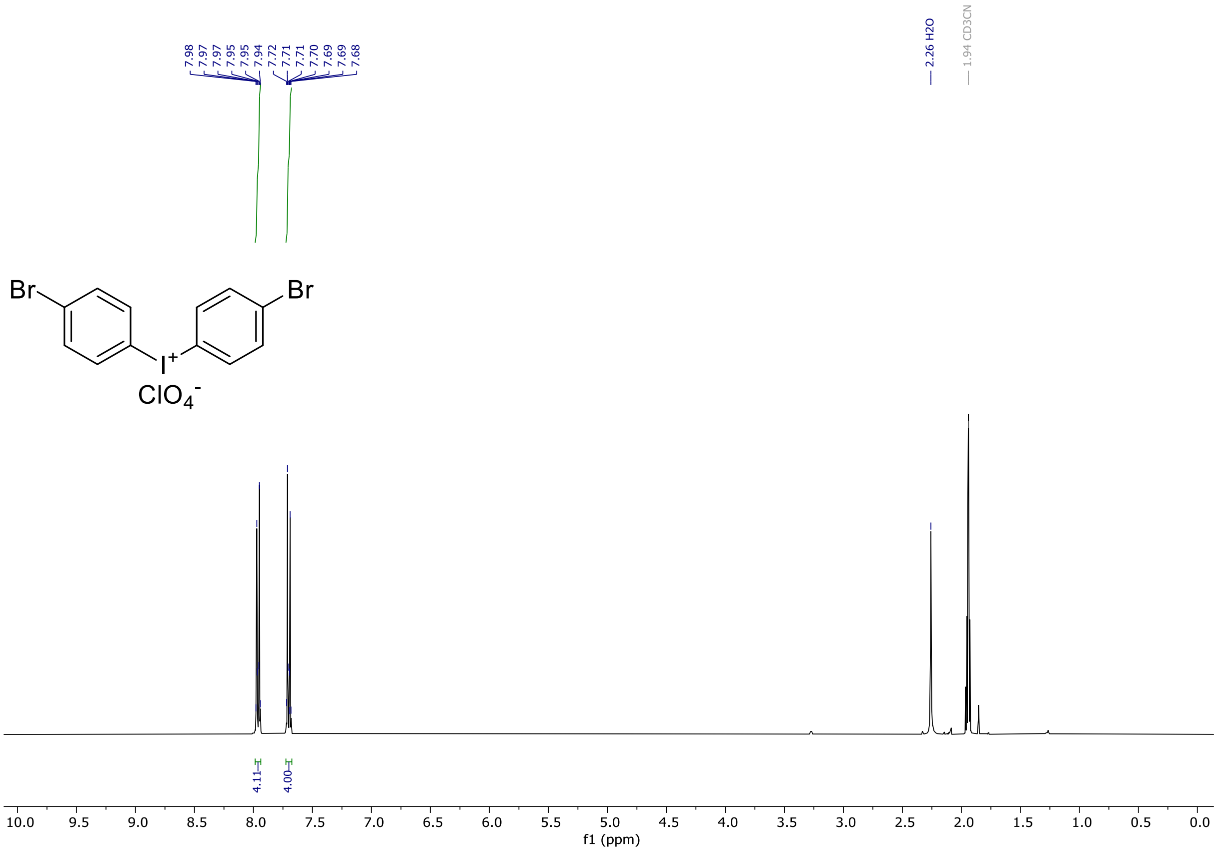

Bis(4-bromophenyl)iodonium perchlorate (**3o**-ClO_4), $^{13}C\{^1H\}$ NMR spectrum (101 MHz, CD_3CN)

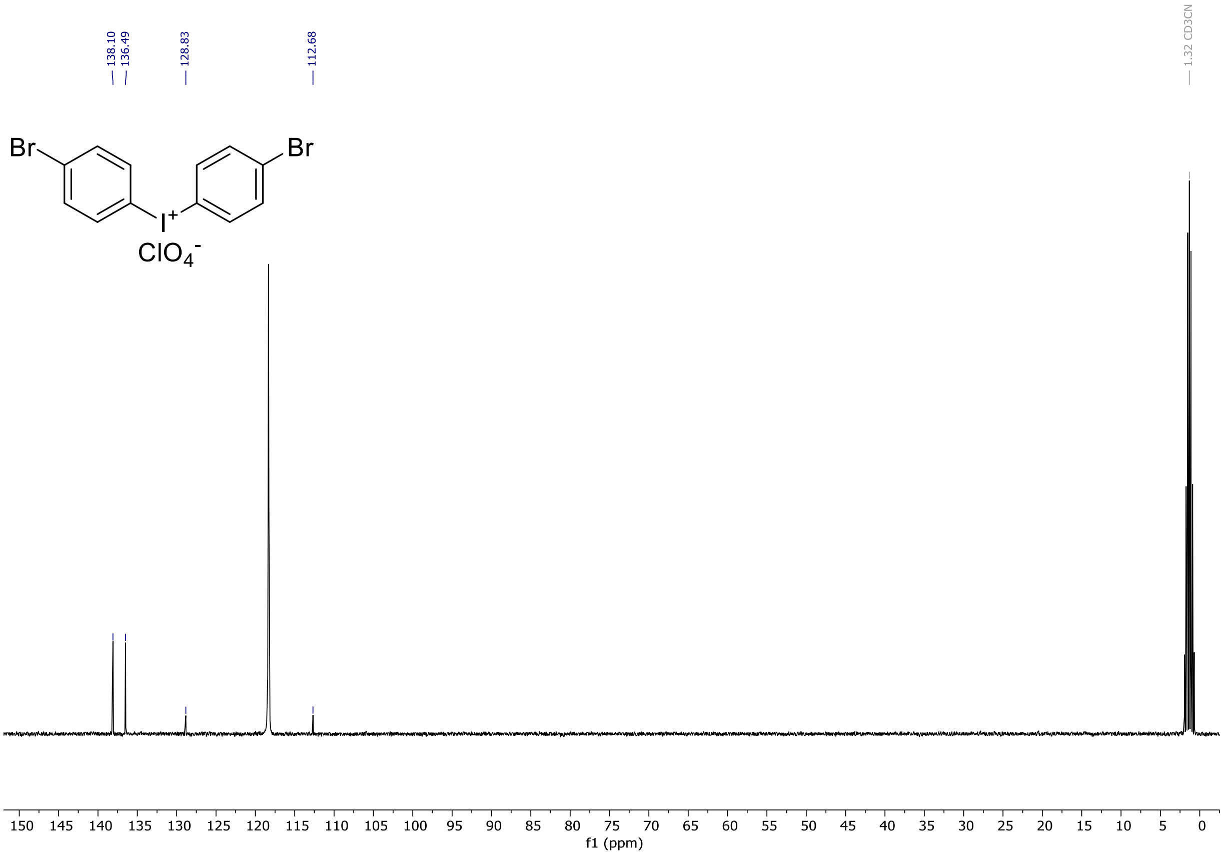

(4-(*tert*-Butyl)phenyl)(mesityl)iodonium perchlorate (**3p**-ClO_4), ^{1}H NMR spectrum (400 MHz, CD_3CN)

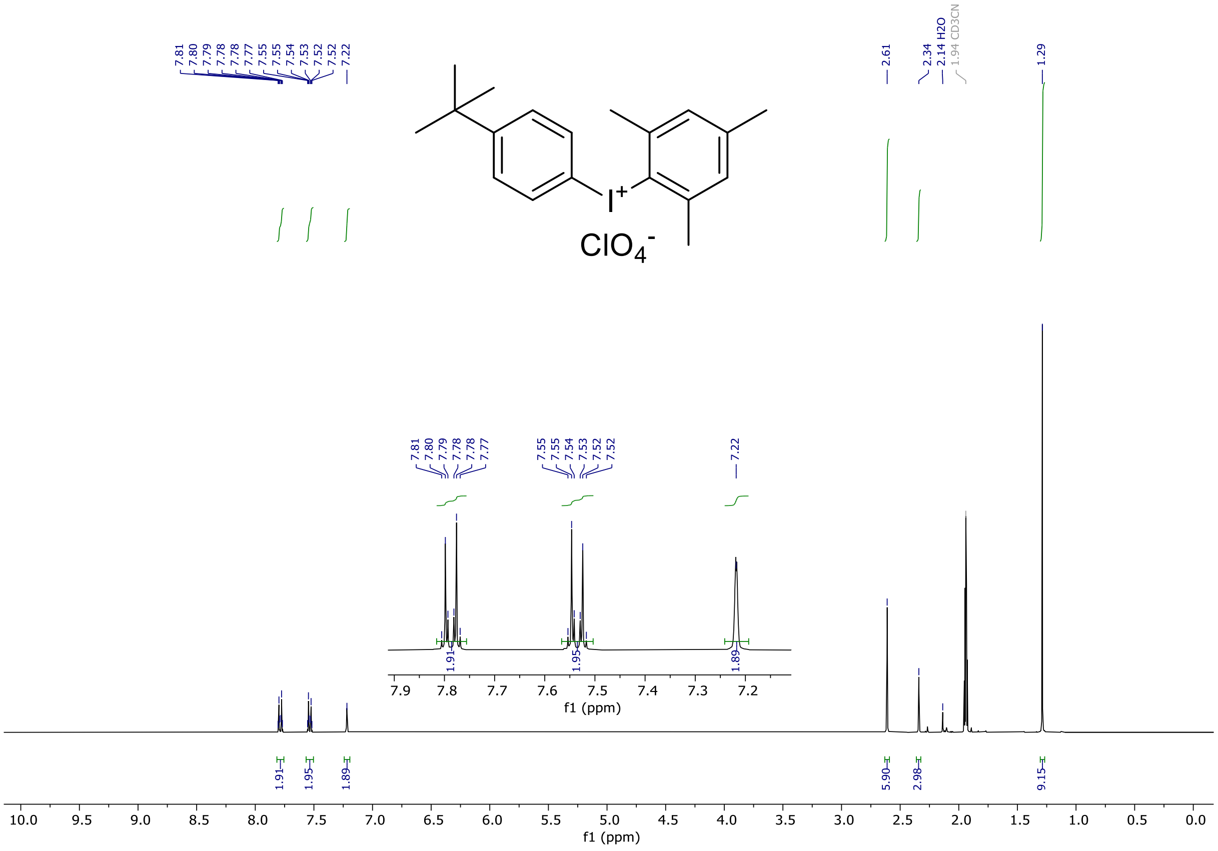

(4-(*tert*-Butyl)phenyl)(mesityl)iodonium perchlorate (**3p**-ClO_4), ^{13}C{^{1}H} NMR (101 MHz, CD_3CN)

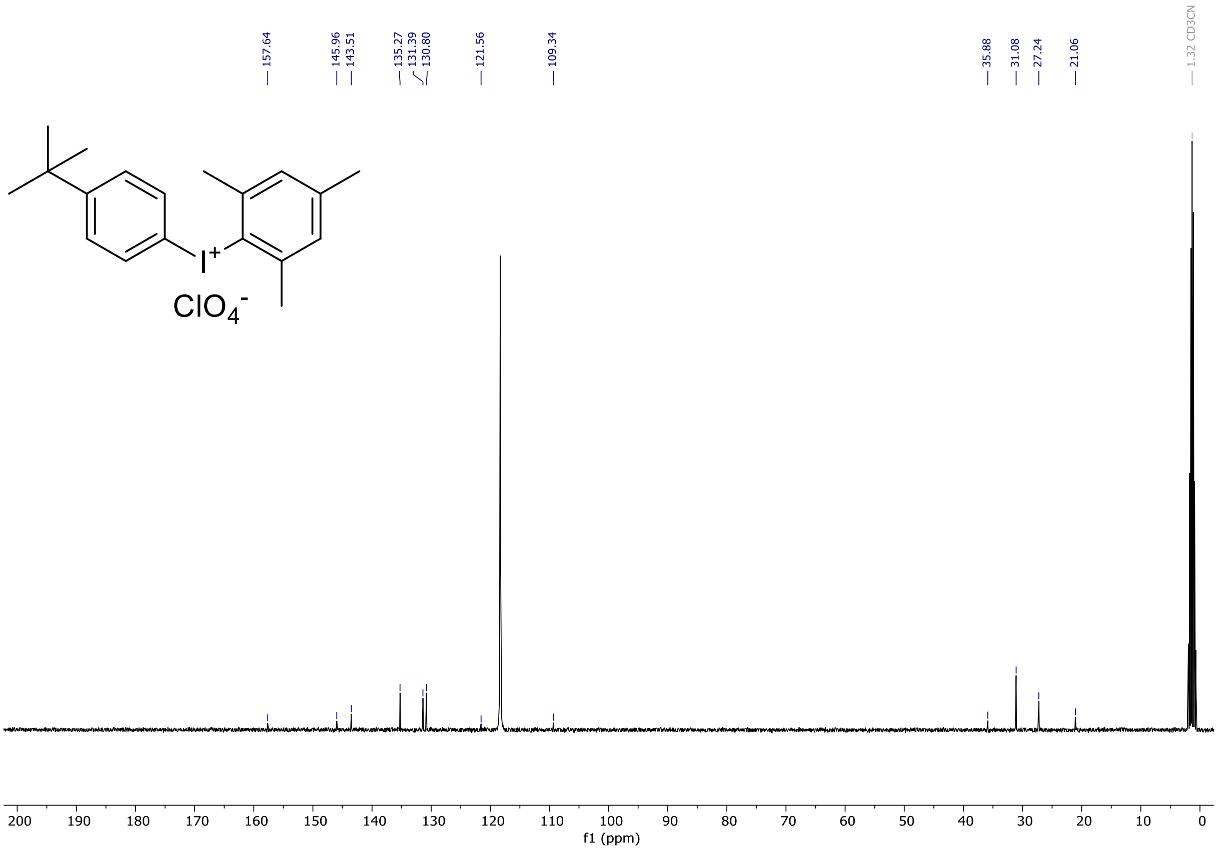

Dibenzo[*b*,*d*]iodol-5-ium perchlorate (**5a**-ClO_4), ^{1}H NMR spectrum (400 MHz, CD_3CN)

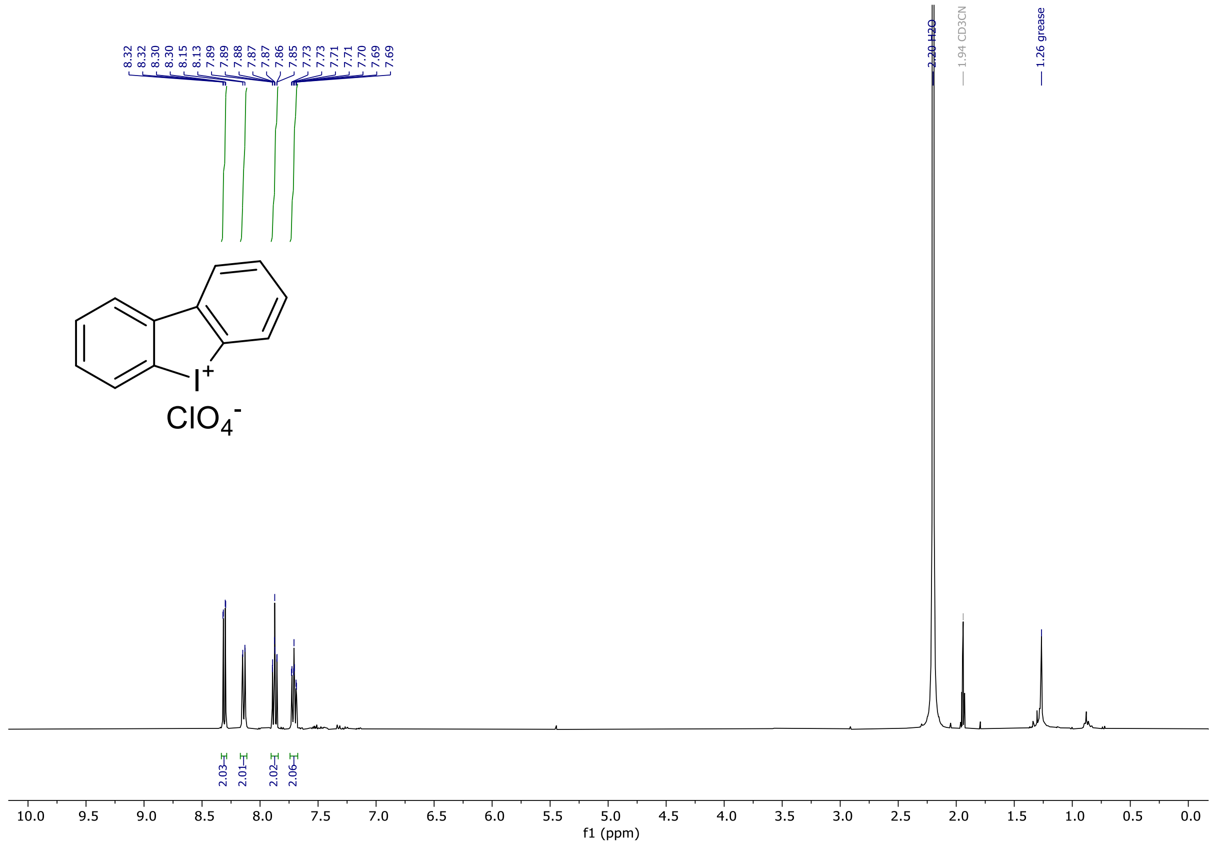

Dibenzo[*b*,*d*]iodol-5-ium perchlorate (**5a**-ClO_4), $^{13}C\{^1H\}$ NMR spectrum (101 MHz, CD_3CN)

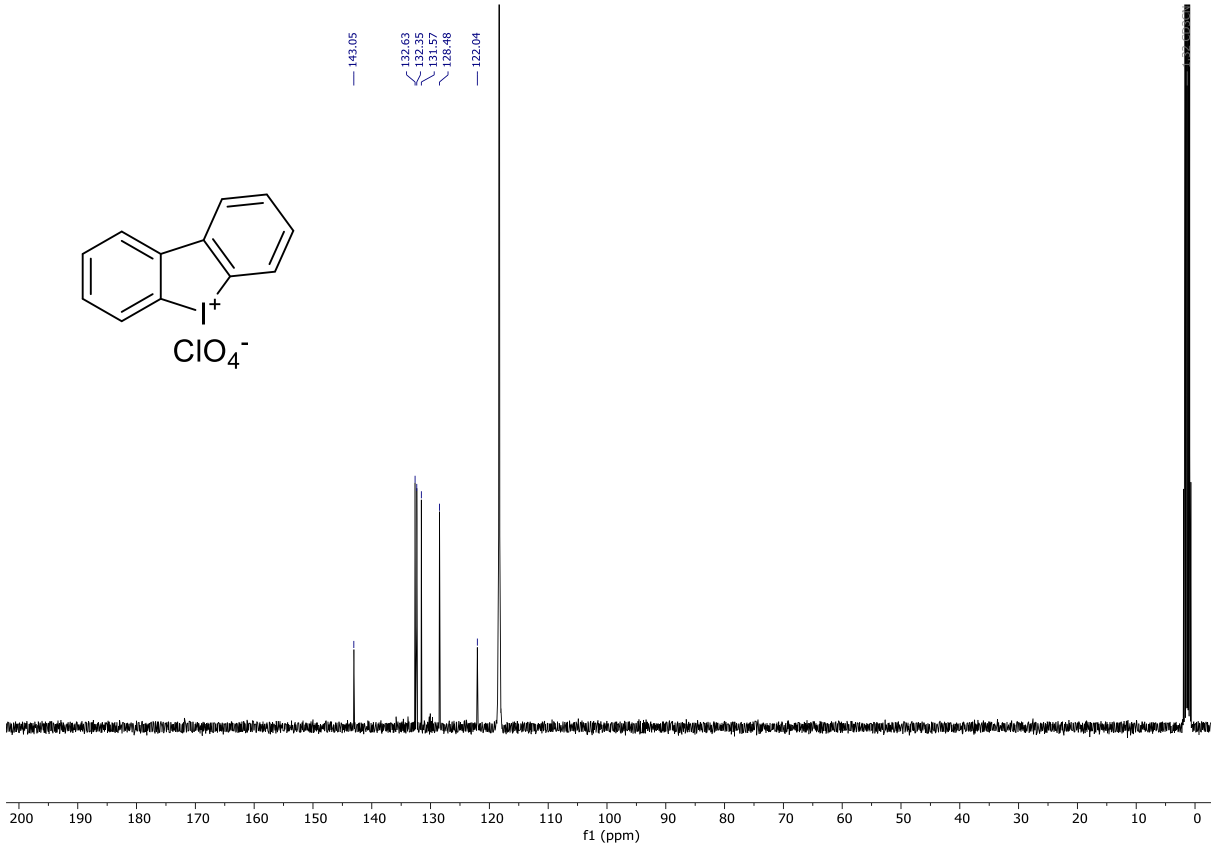

1-Methyldibenzo[*b*,*d*]iodol-5-ium perchlorate (**5b**-ClO_4), ^{1}H NMR spectrum (400 MHz, DMSO-d$_6$)

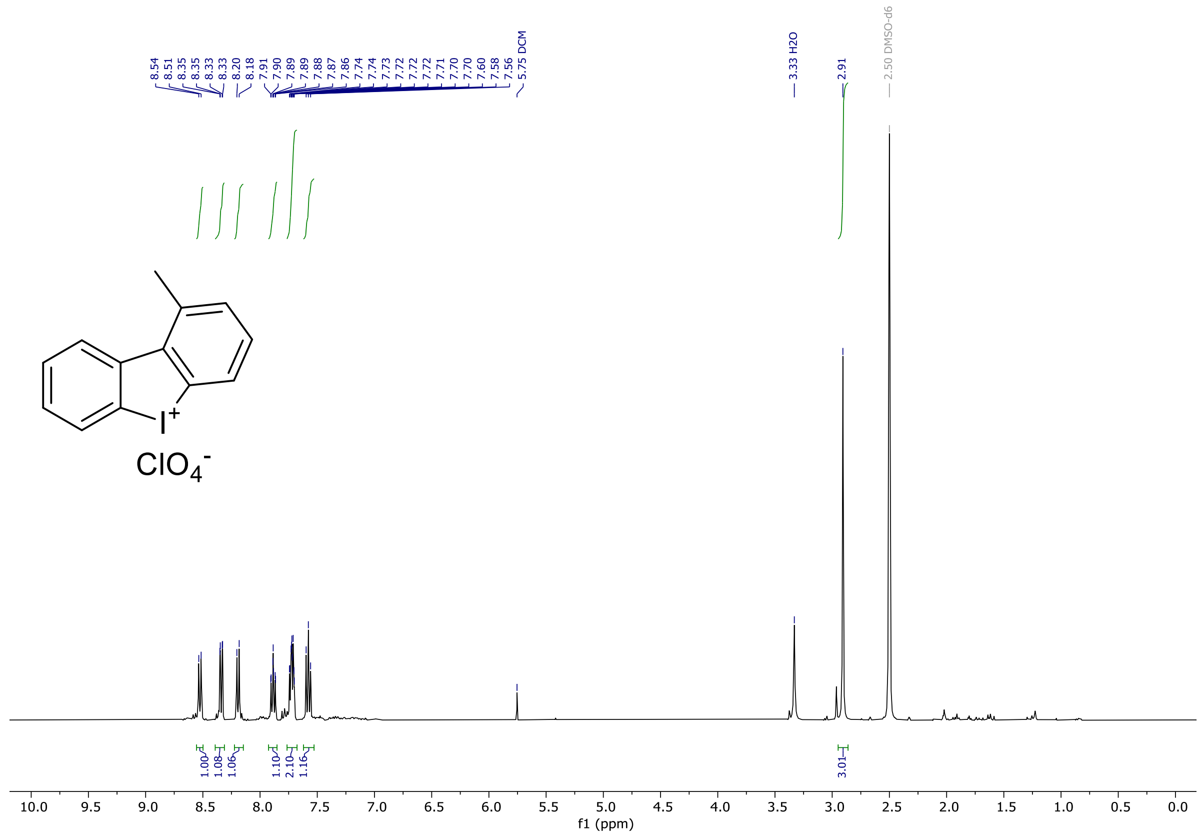

1-Methyldibenzo[*b*,*d*]iodol-5-ium perchlorate (**5b**-ClO_4), $^{13}C\{^{1}H\}$ NMR spectrum (101 MHz, DMSO-*d*$_6$)

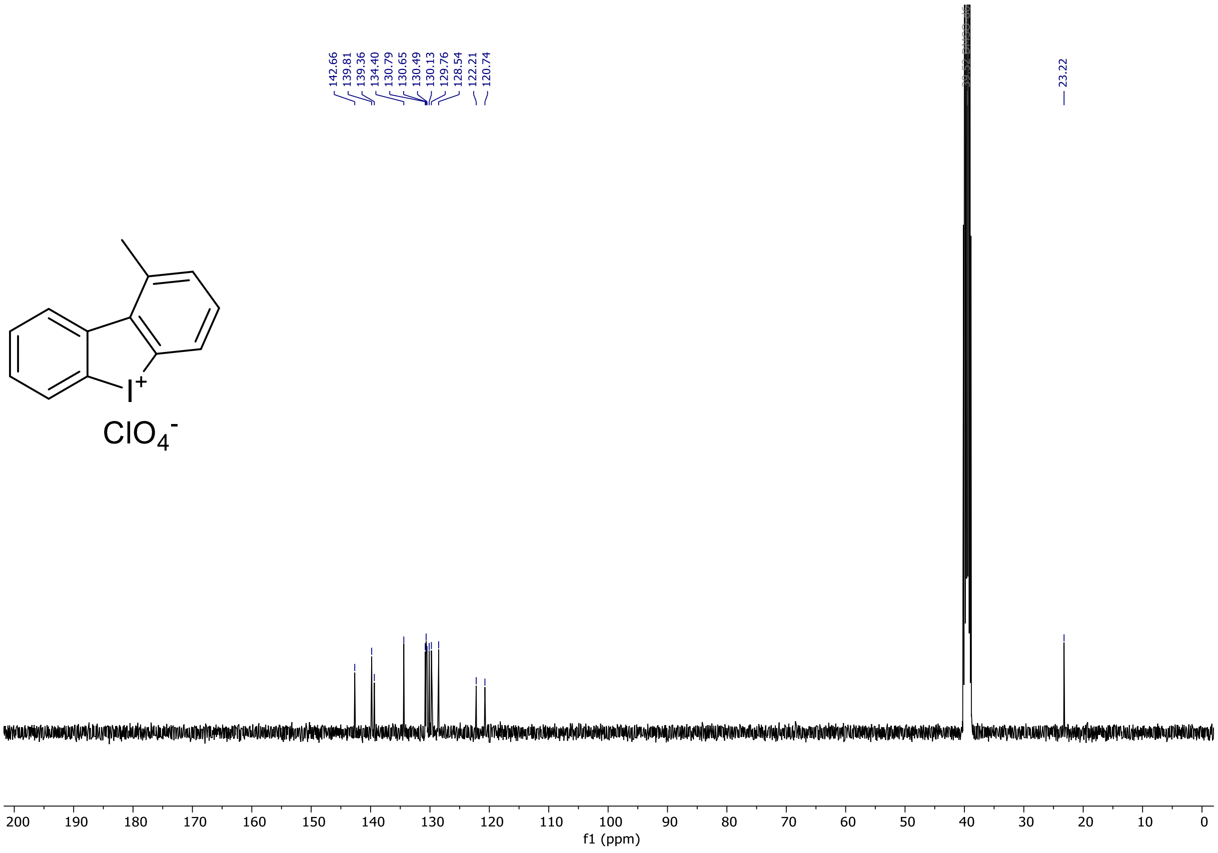

7-Chloro-2-methyldibenzo[*b*,*d*]iodol-5-ium perchlorate (**5c**-ClO_4), ^{1}H NMR spectrum (400 MHz, DMSO-d_6)

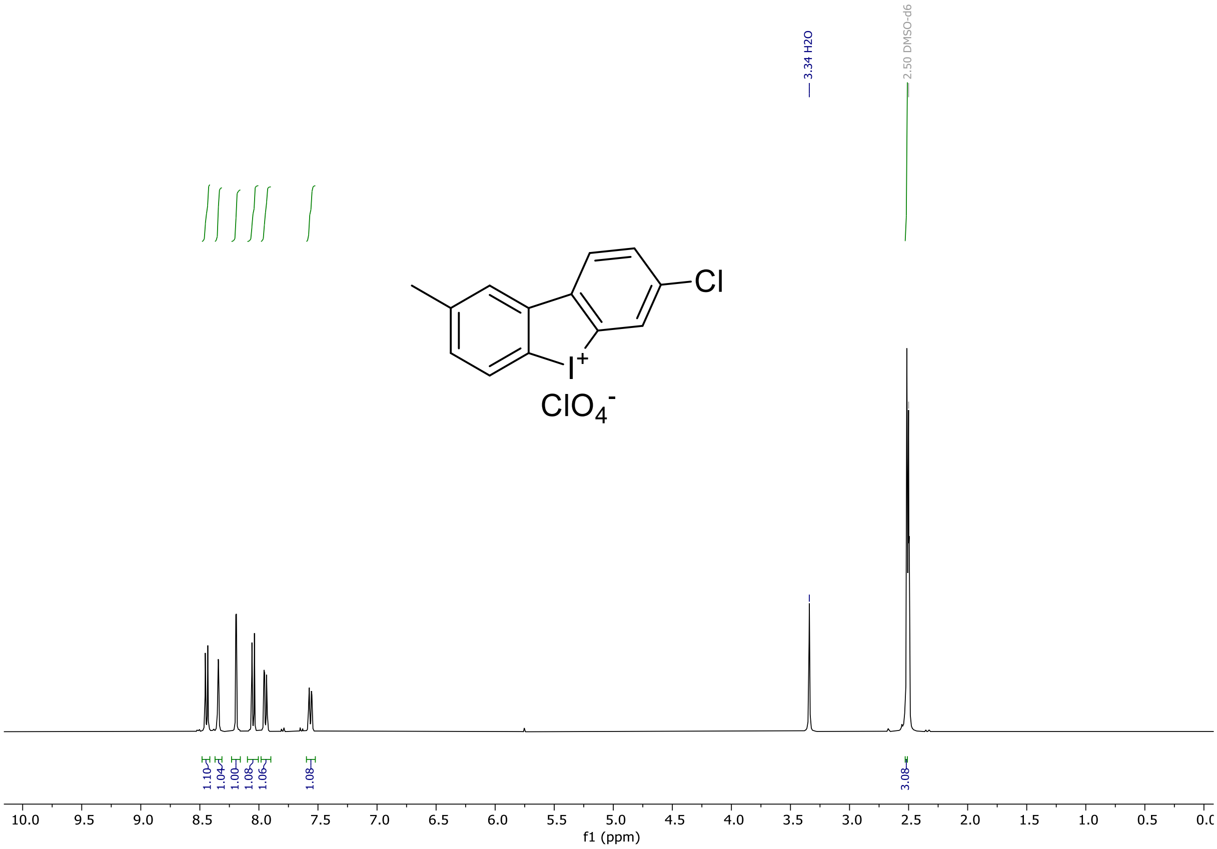

7-Chloro-2-methyldibenzo[*b*,*d*]iodol-5-ium perchlorate (**5c**-ClO_4), $^{13}C\{^1H\}$ NMR spectrum (101 MHz, DMSO-d_6)

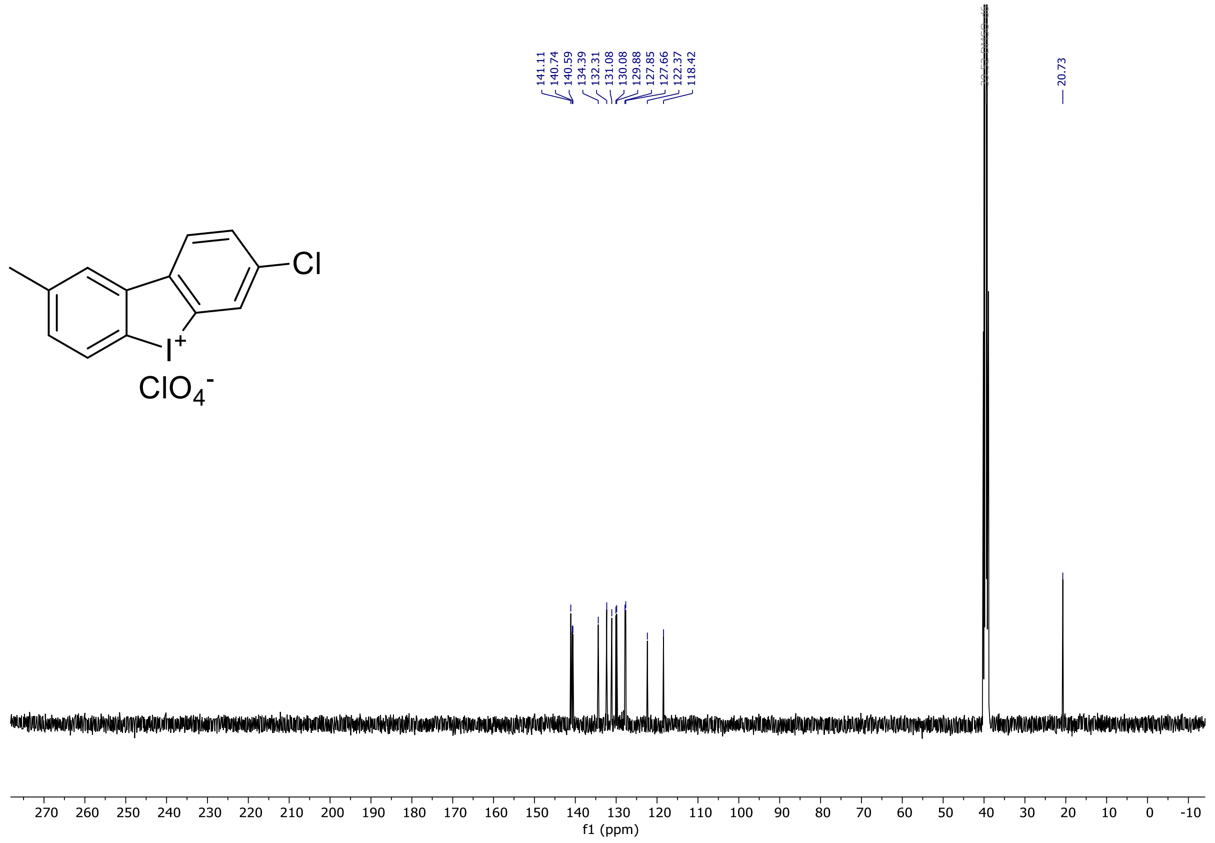

3-Chlorodibenzo[*b*,*d*]iodol-5-ium perchlorate (**5d**-ClO_4), ^1H NMR spectrum (400 MHz, CD_3CN)

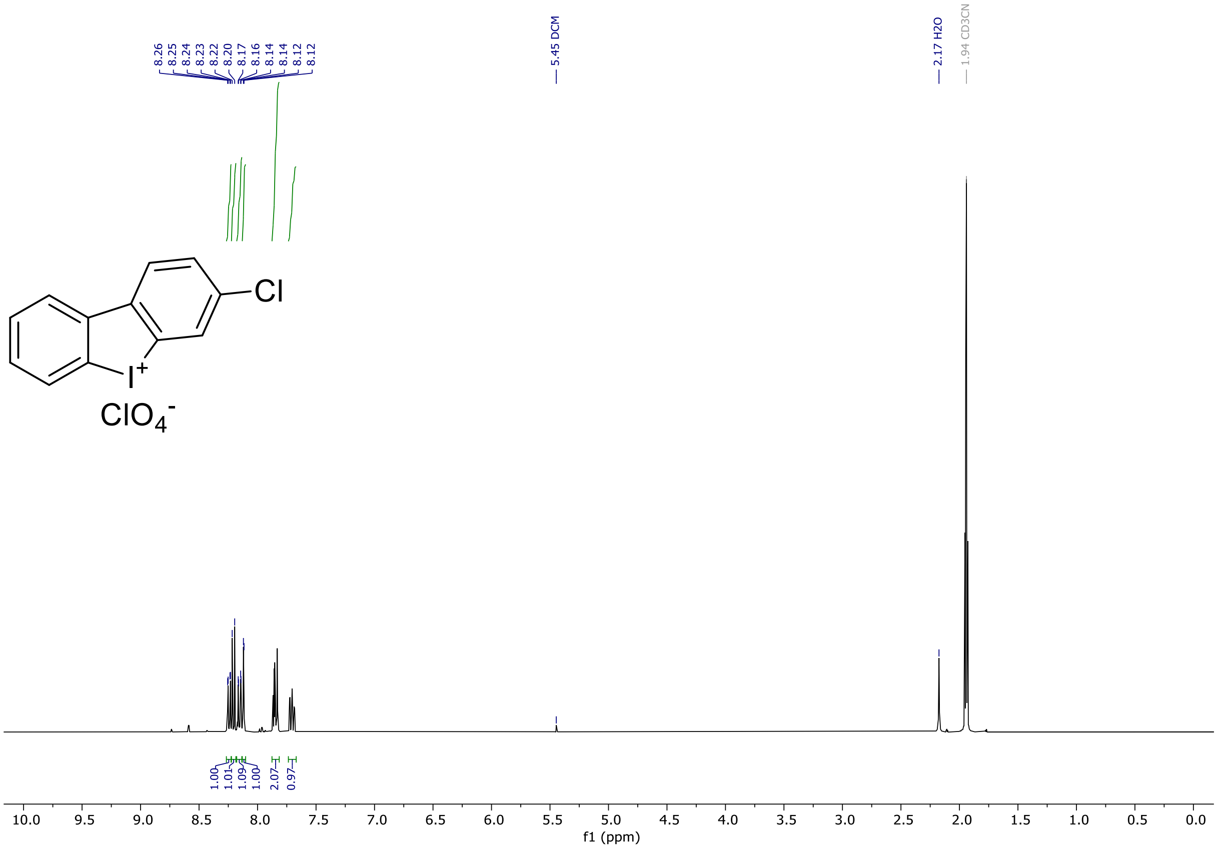

3-Chlorodibenzo[*b*,*d*]iodol-5-ium perchlorate (**5d**-ClO_4), $^{13}C\{^1H\}$ NMR spectrum (101 MHz, CD_3CN)

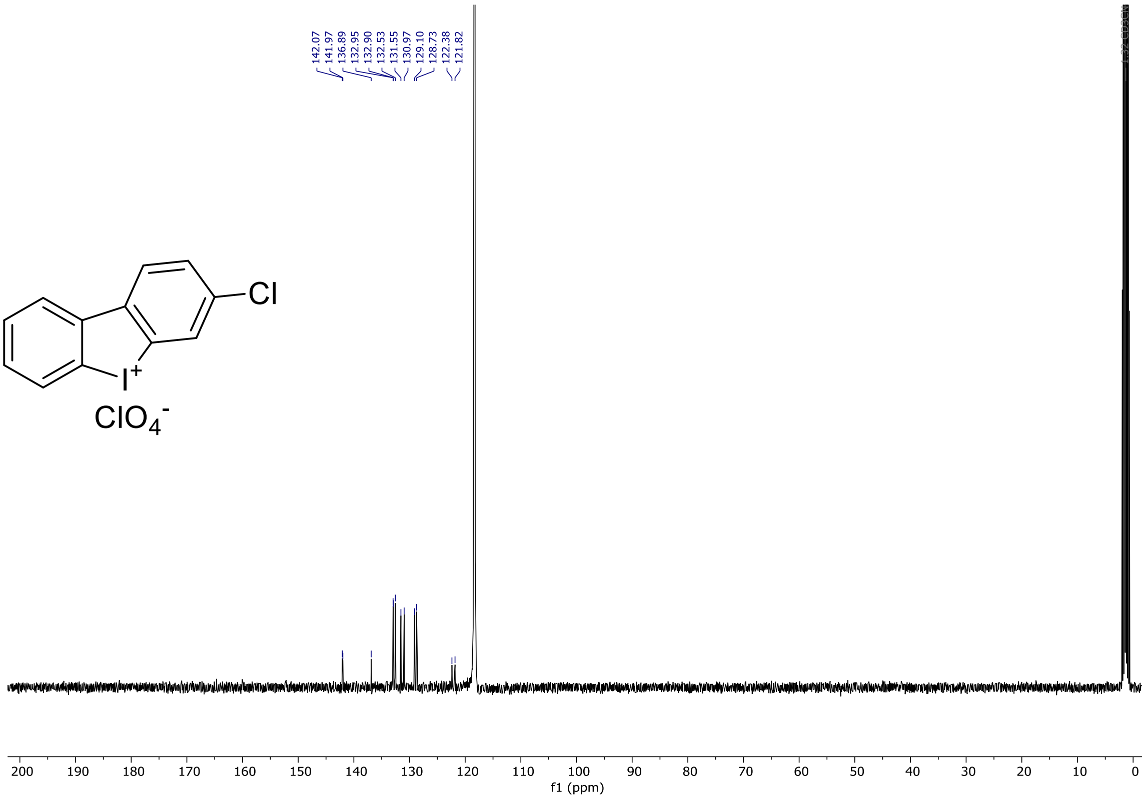

1-Bromo-4-phenoxybenzene (**7**), ^{1}H NMR spectrum (300 MHz, CD_2Cl_2)

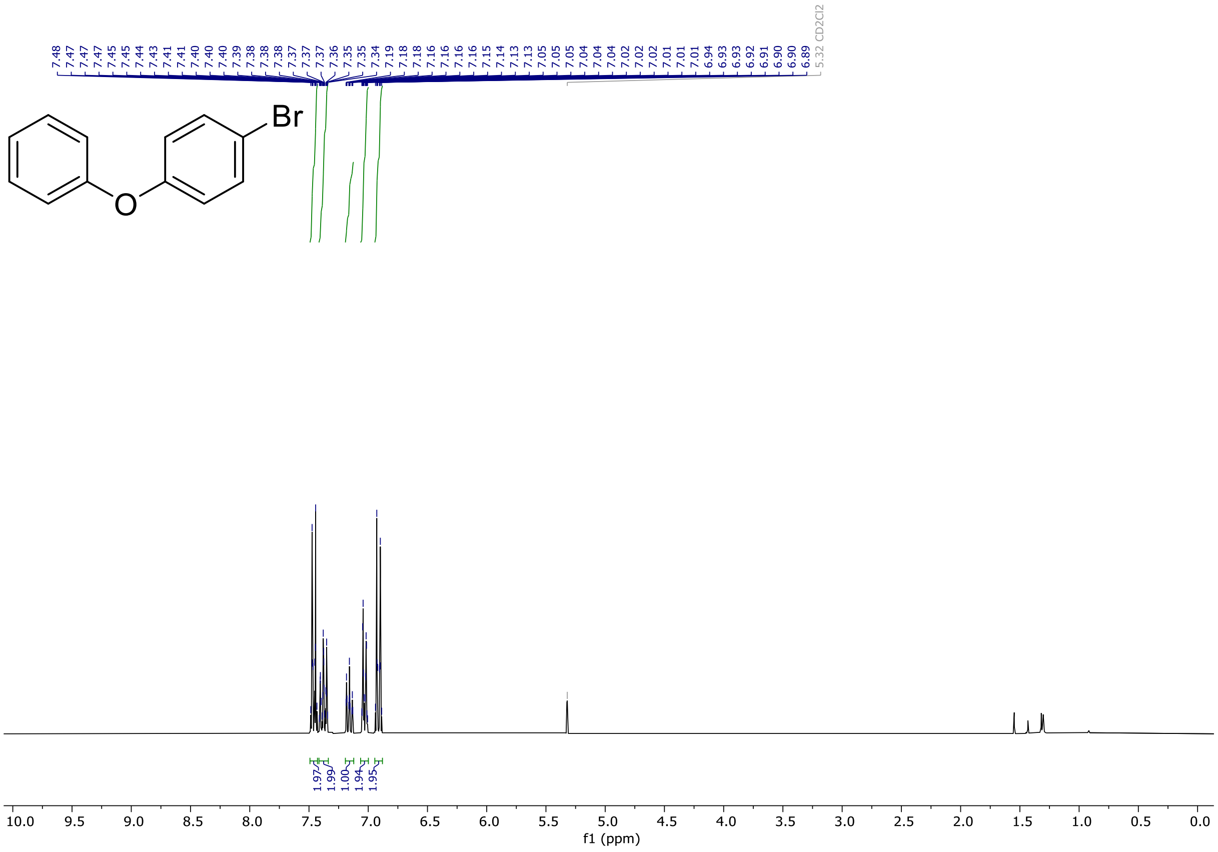

1-Bromo-4-phenoxybenzene (**7**), $^{13}C\{^{1}H\}$ NMR spectrum (75 MHz, CD_2Cl_2)

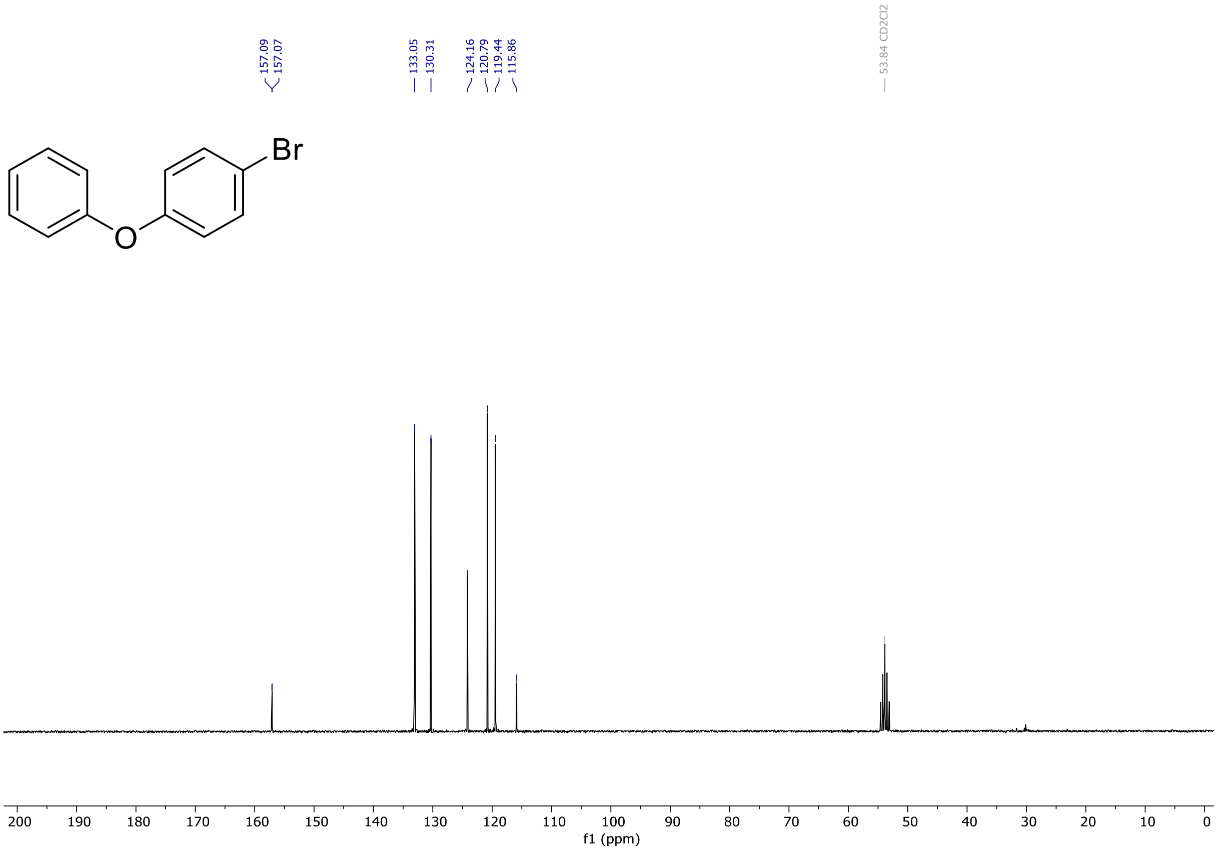

2-(4-Iodophenyl)-2-methyl-1,3-dioxolane, ^{1}H NMR spectrum (300 MHz, CD_2Cl_2)

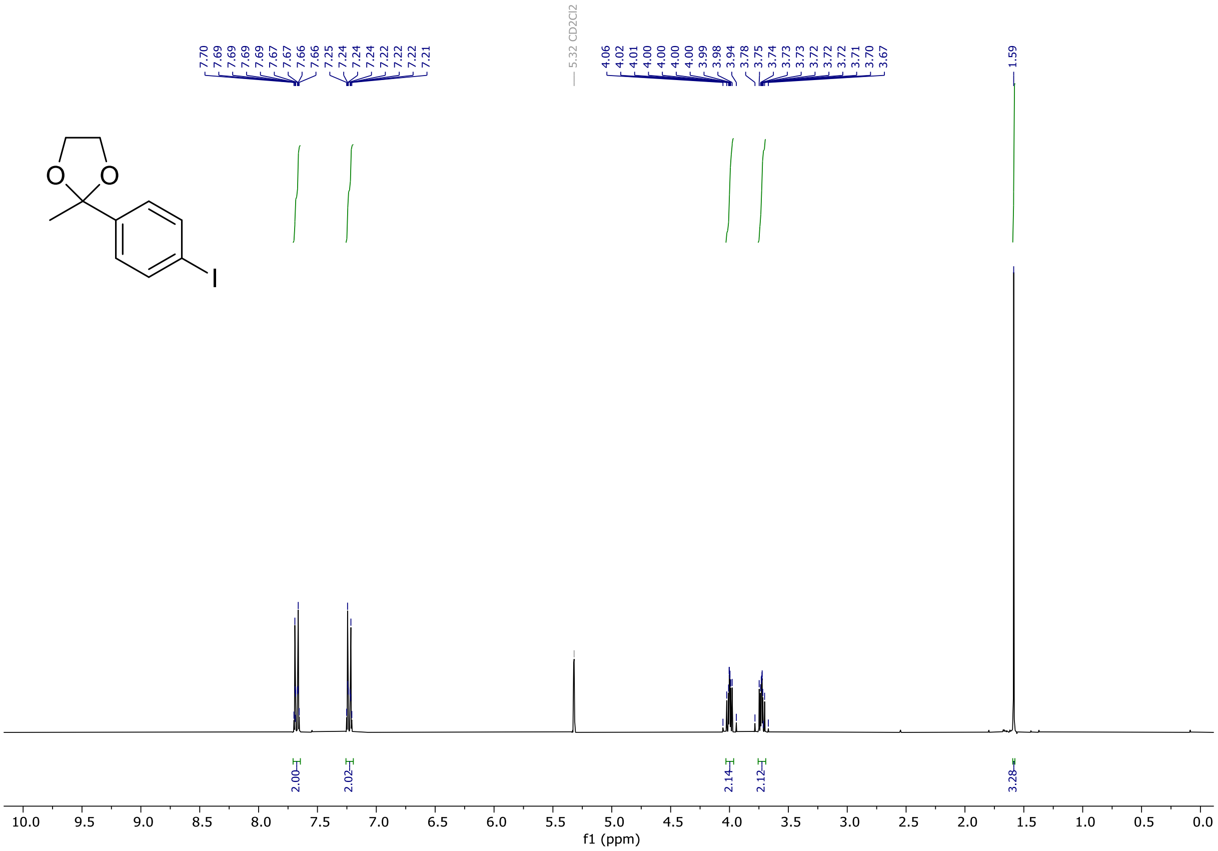

2-(4-Iodophenyl)-2-methyl-1,3-dioxolane, $^{13}C\{^{1}H\}$ NMR spectrum (75 MHz, CD_2Cl_2)

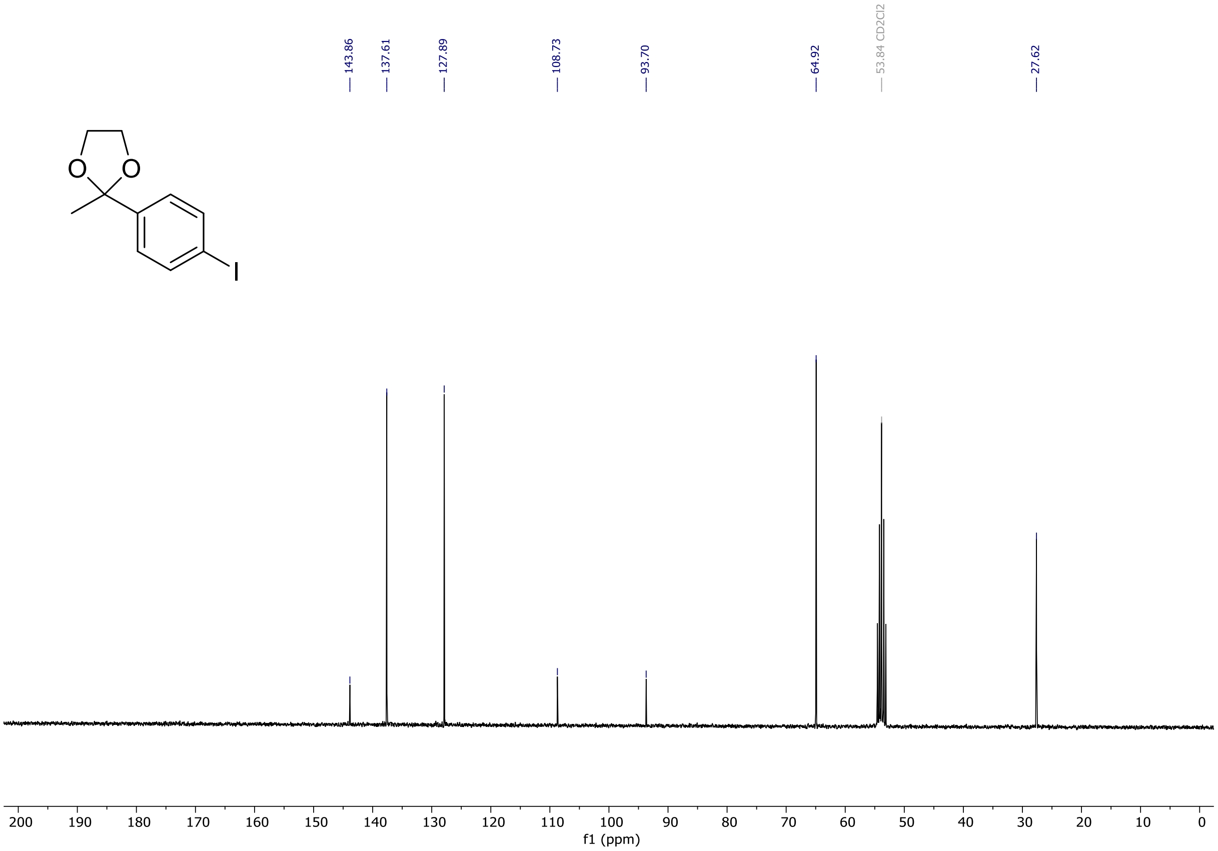

2-Amino-2'-methyl-1,1'-biphenyl (**A3b**), ^{1}H NMR spectrum (300 MHz, $CDCl_3$)

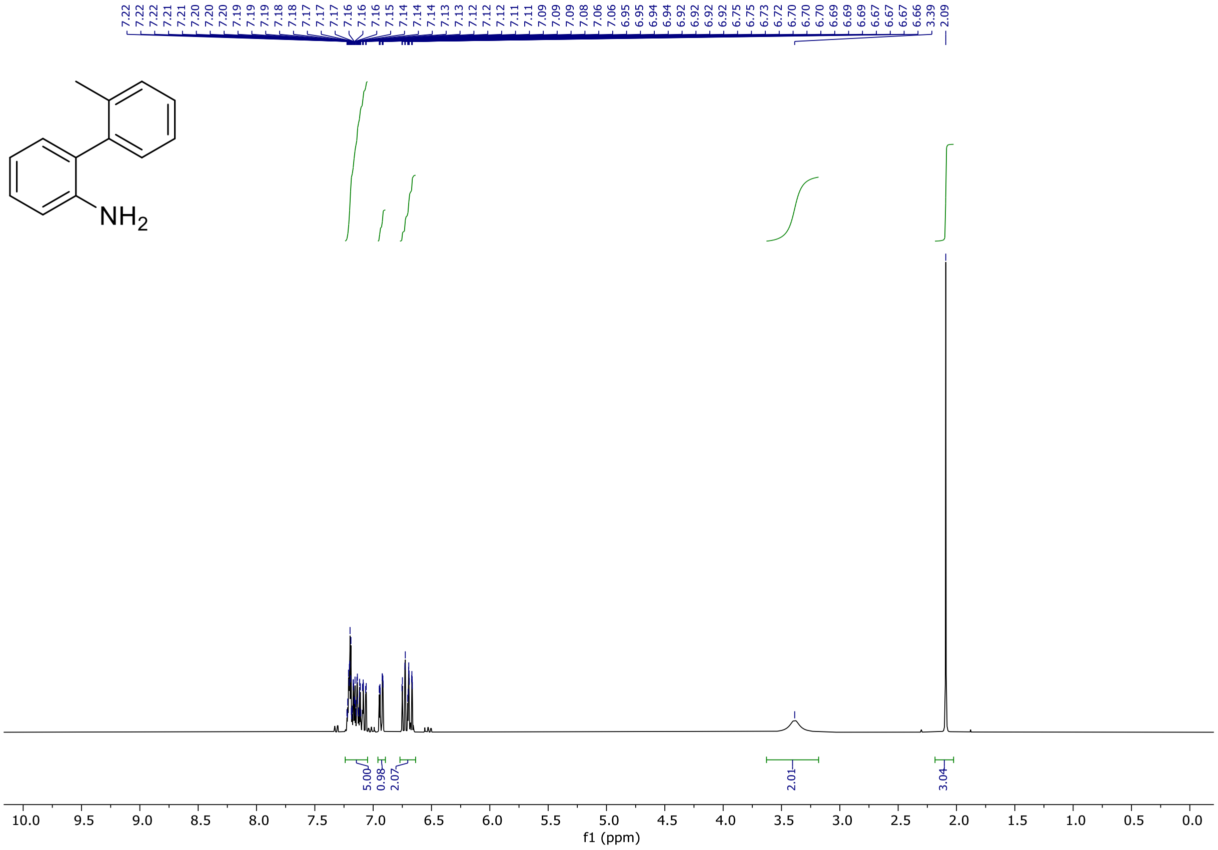

2-Amino-2'-methyl-1,1'-biphenyl (**A3b**), $^{13}C\{^{1}H\}$ NMR spectrum (75 MHz, $CDCl_3$)

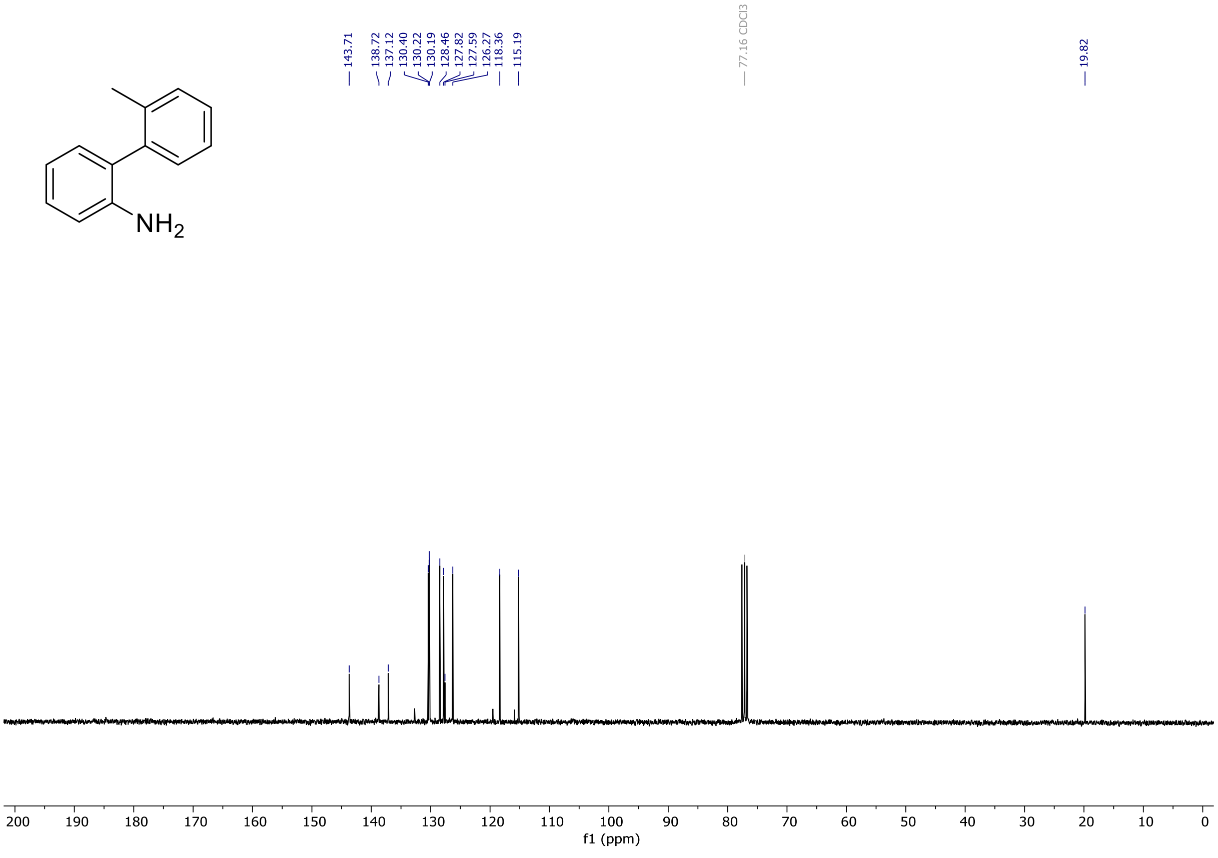

2-Amino-4'-chloro-5-methyl-1,1'-biphenyl (**A3c**), ^{1}H NMR spectrum (300 MHz, $CDCl_3$)

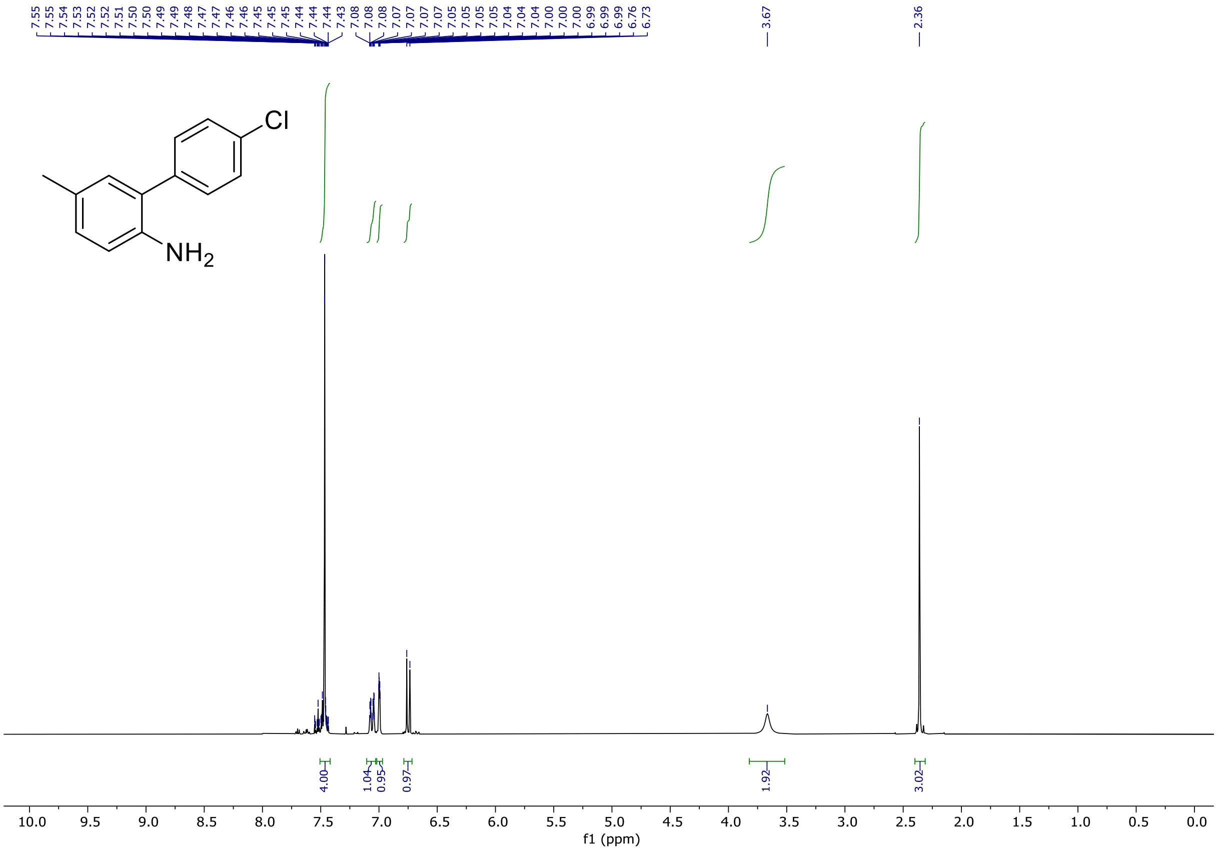

2-Amino-4'-chloro-5-methyl-1,1'-biphenyl (**A3c**), $^{13}C\{^{1}H\}$ NMR spectrum (75 MHz, $CDCl_3$)

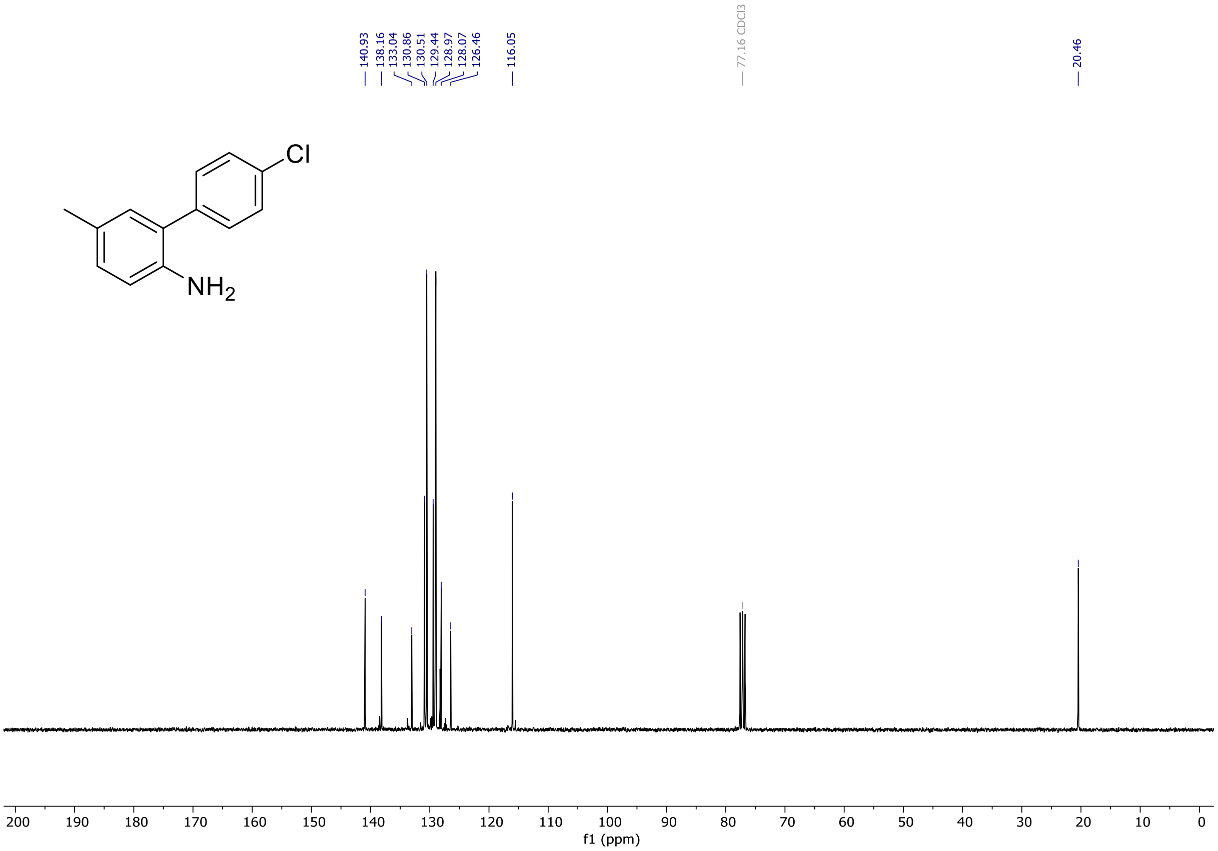

2-Iodo-2'-methyl-1,1'-biphenyl (**4b**), ^{1}H NMR spectrum (300 MHz, $CDCl_3$)

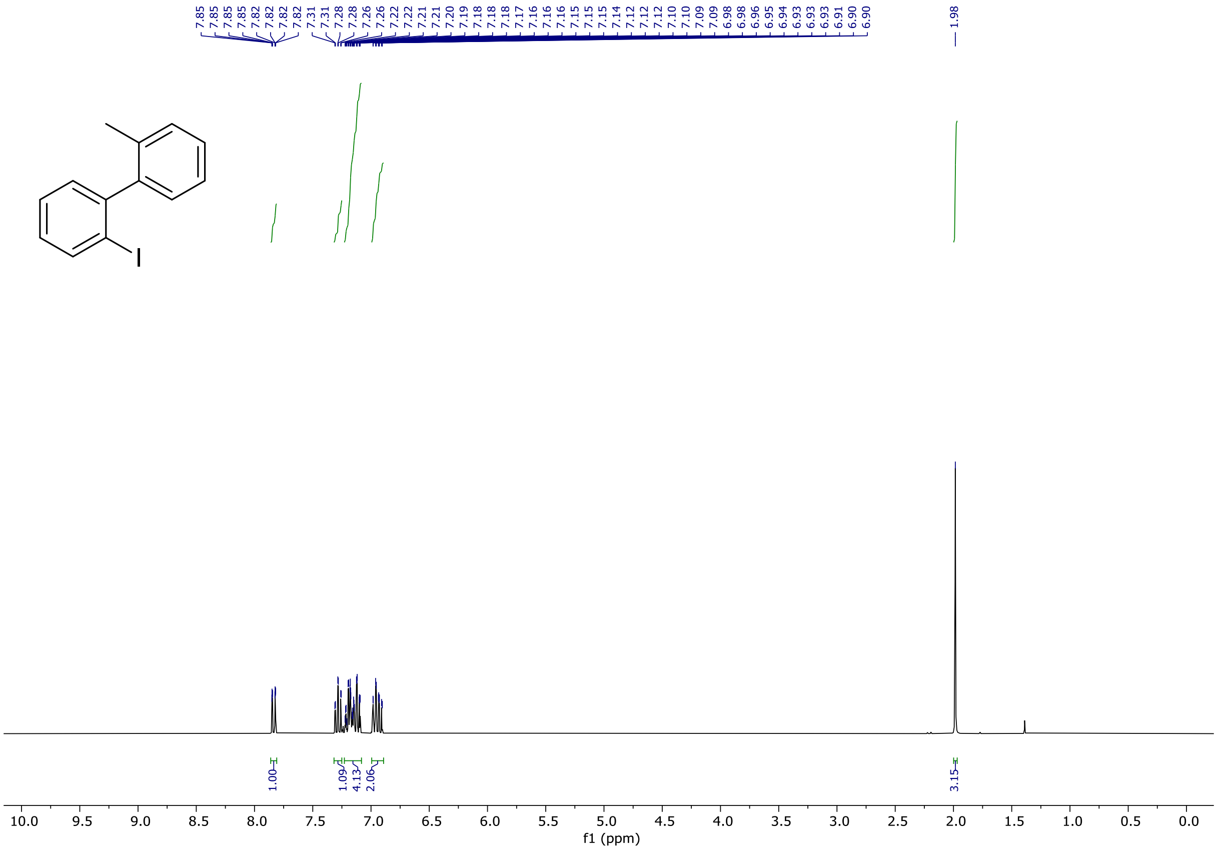

2-Iodo-2'-methyl-1,1'-biphenyl (**4b**), $^{13}C\{^{1}H\}$ NMR spectrum (75 MHz, $CDCl_3$)

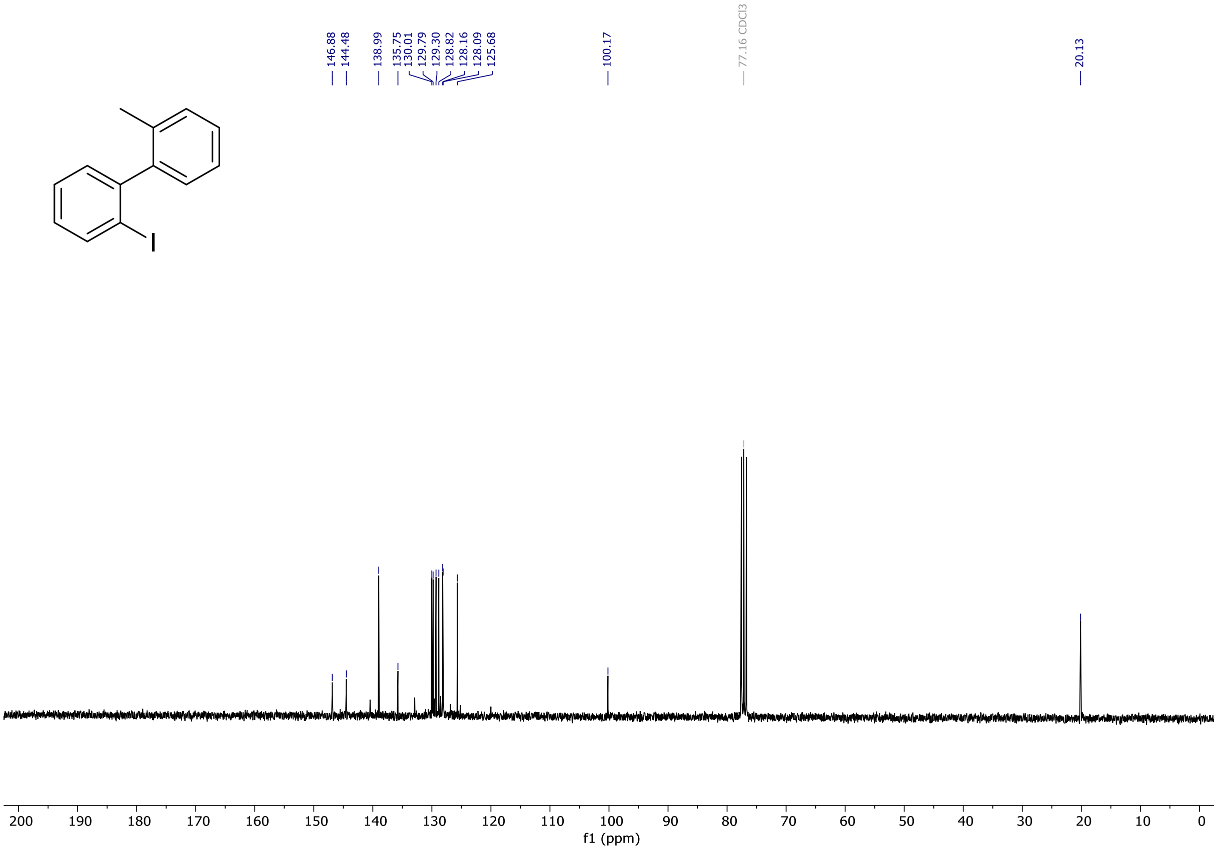

4'-Chloro-2-iodo-5-methyl-1,1'-biphenyl (**4c**), ^{1}H NMR spectrum (300 MHz, $CDCl_3$)

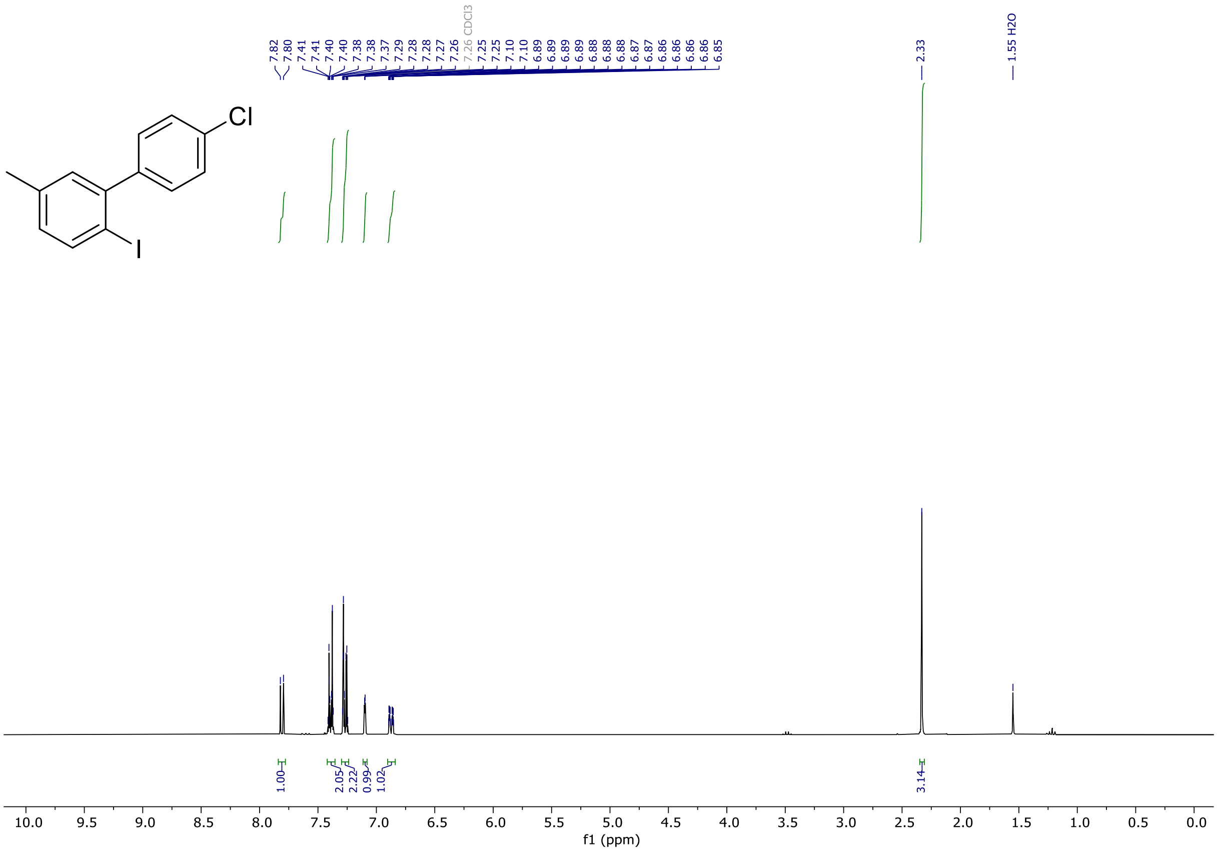

4'-Chloro-2-iodo-5-methyl-1,1'-biphenyl (**4c**), $^{13}C\{^{1}H\}$ NMR spectrum (75 MHz, $CDCl_3$)

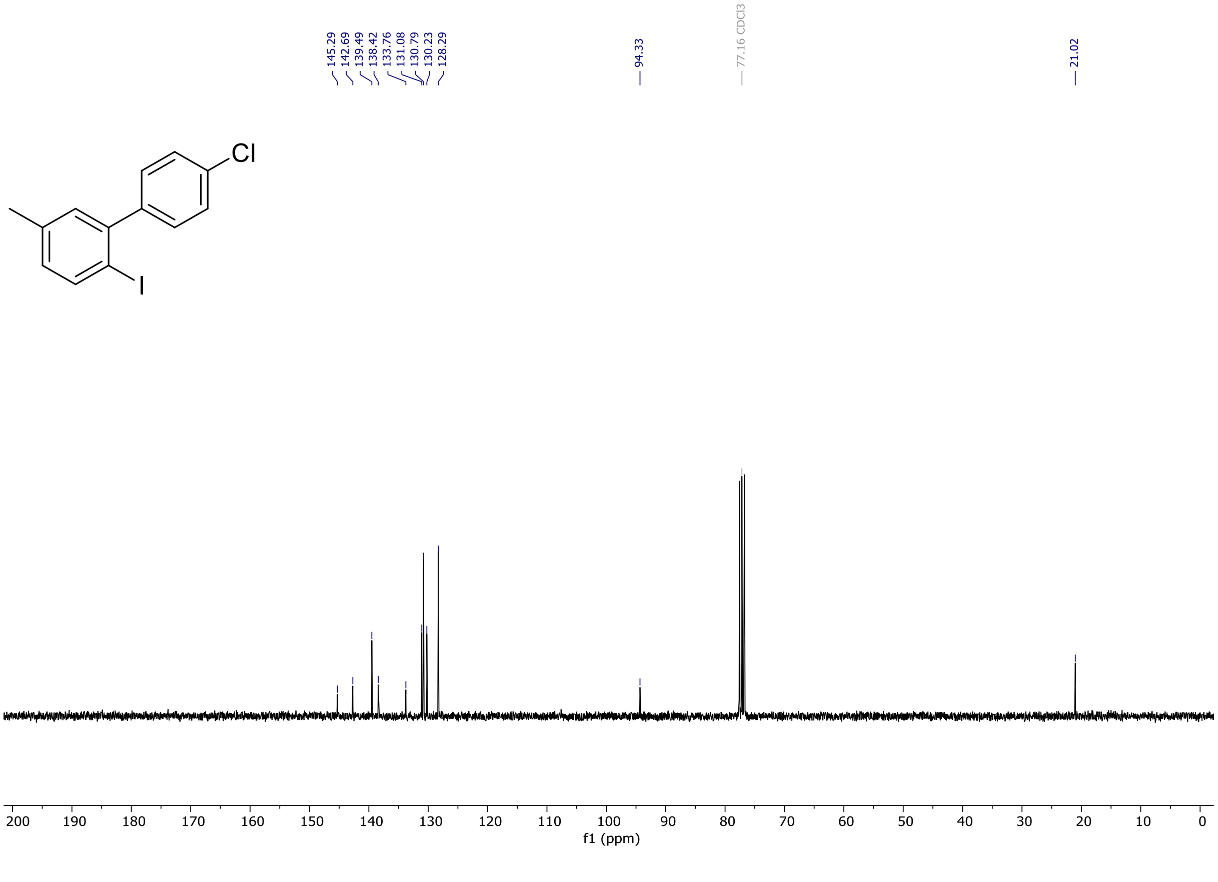

4'-Chloro-2-iodo-1,1'-biphenyl (**4d**), ^{1}H NMR spectrum (300 MHz, $CDCl_3$)

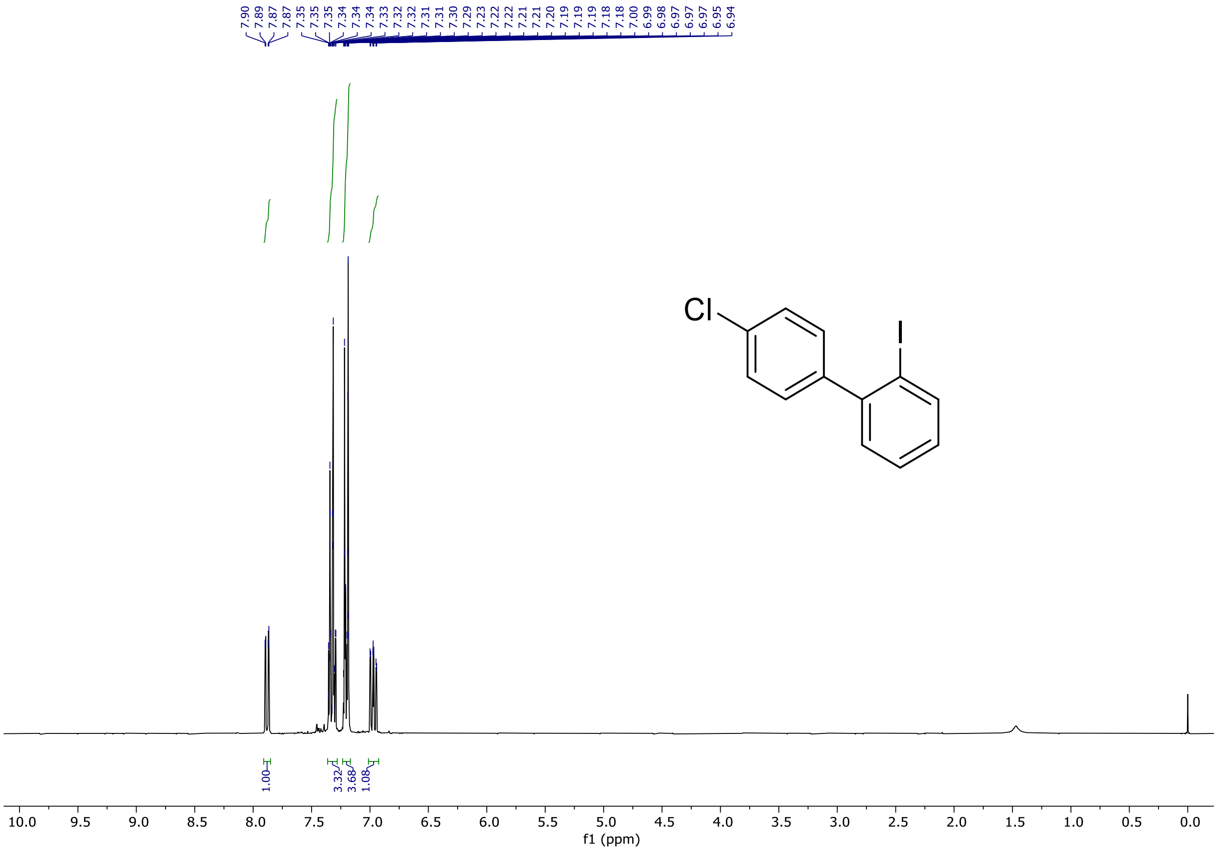

4'-Chloro-2-iodo-1,1'-biphenyl (**4d**), $^{13}C\{^{1}H\}$ NMR spectrum (75 MHz, $CDCl_3$)

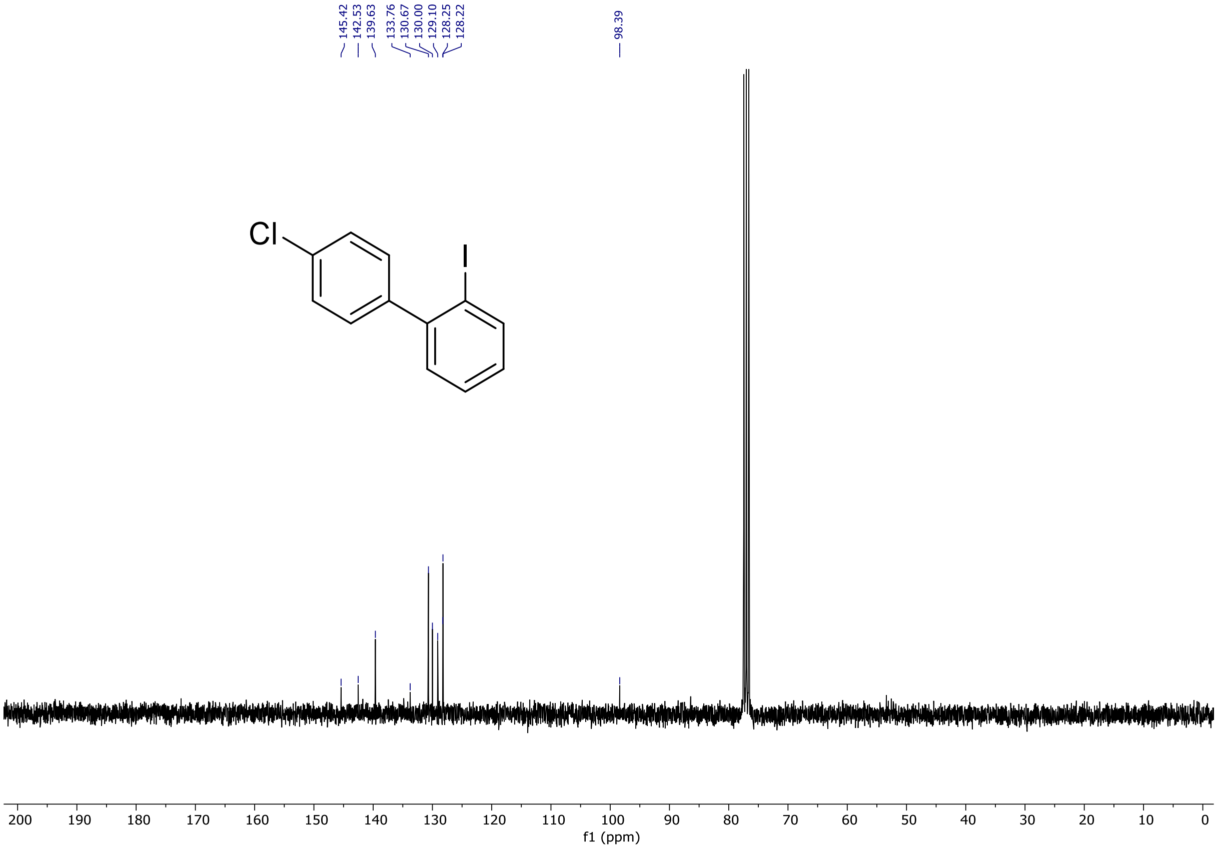

2,2'-Diiodobiphenyl (**4e**), ^{1}H NMR spectrum (400 MHz, $CDCl_3$)

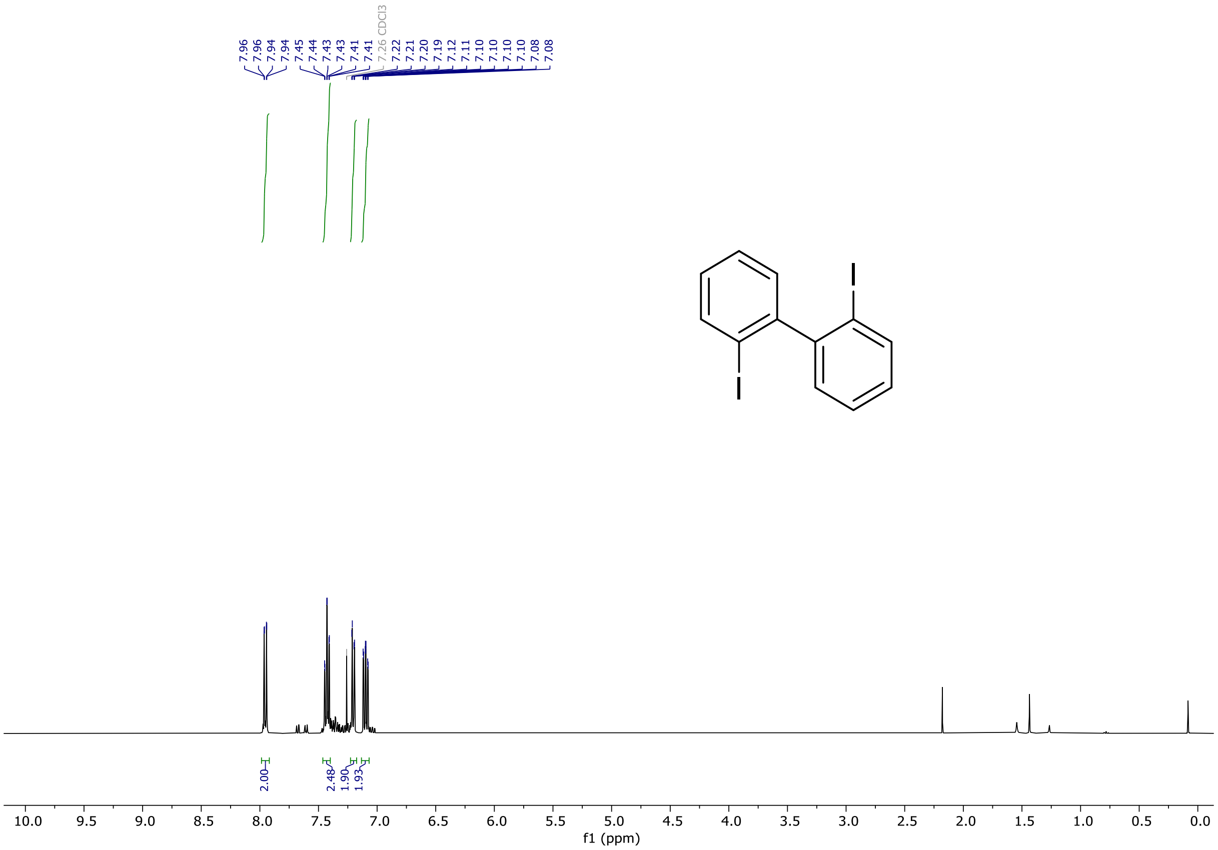

2,2'-Diiodobiphenyl (**4e**), $^{13}C\{^{1}H\}$ NMR spectrum (101 MHz, $CDCl_3$)

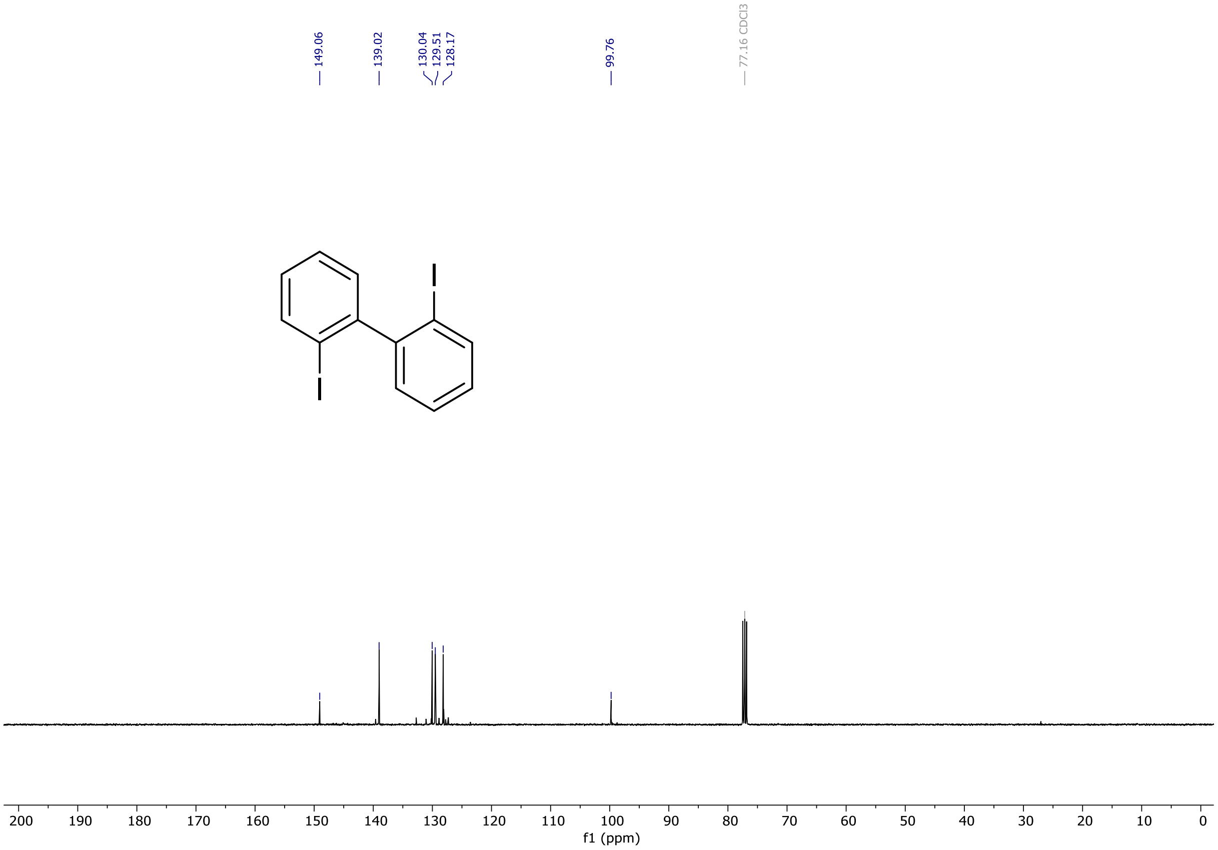

5. Powder XRD patterns

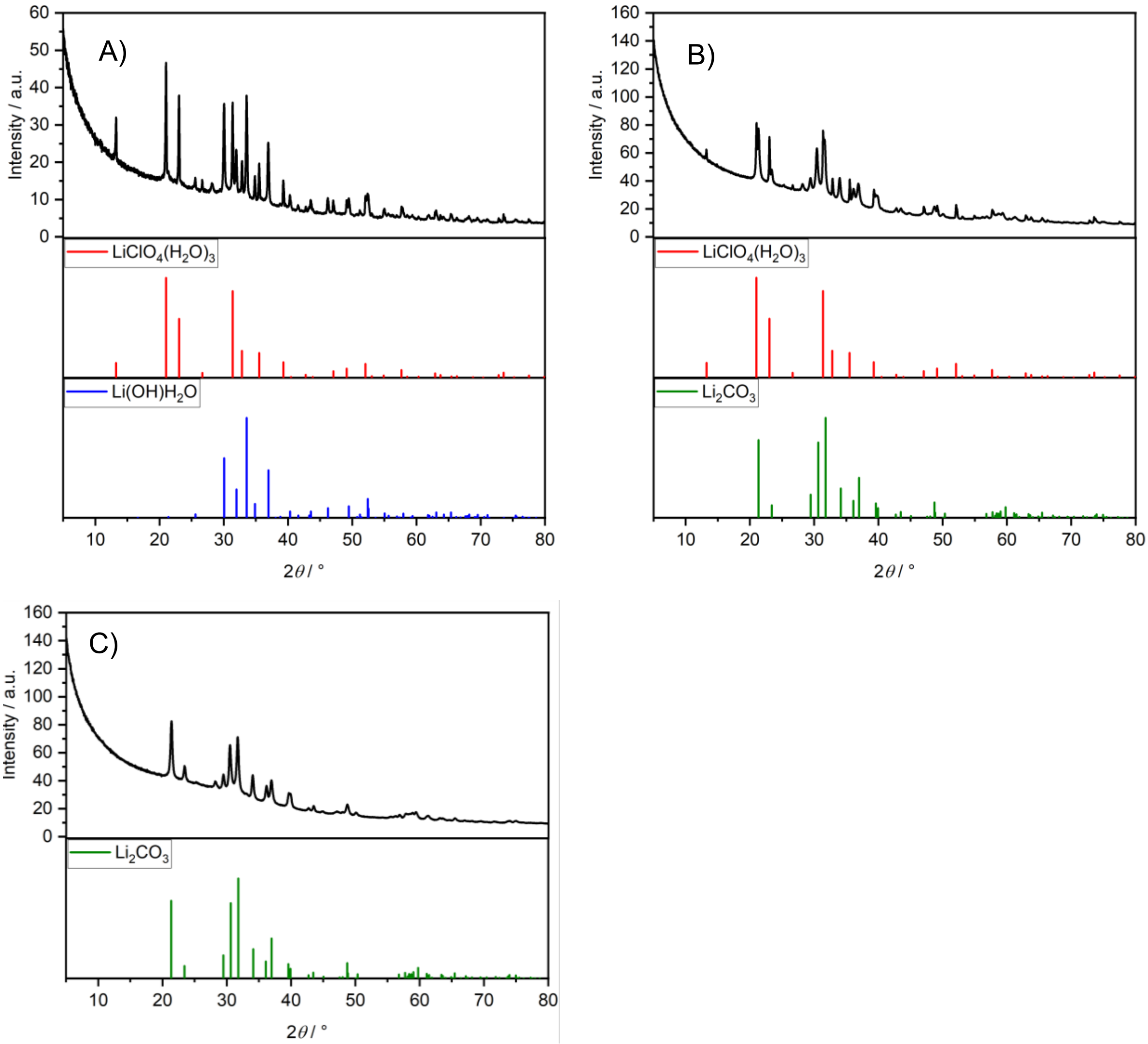

Figure S3. Powder-XRD patterns of the precipitates formed during electrolysis in the cathode compartment, showing the transition from LiOH to $LiCO_3$ upon exposure to air from A) to C). A) Initial measurement directly after electrolysis. B) Repeated measurement after 22 minutes using the same sample. C: Repeated measurement after 47 minutes using the same sample. Reference spectra taken from the literature.[11]

[11] a) Lin, Y.-Y.; Zhang, H.-Q.; Zheng, M.-P. The Crystal Structure of Zabuyelite. *Chin. Sci. Bull.* **1990**, *35* 489-492. b) Hermansson, K.; Thomas, J. O. The Experimental Electron Density in Lithium Hydroxide Monohydrate. *Acta Cryst.* **1982**, *38*, 2555. c) Sequeira, A.; Bernal, I.; Brown, I. D.; Faggiani, R. The structure of lithium perchlorate trihydrate $Li(H_2O)_3ClO_4$ – an X-ray and neutron diffraction study. *Acta Cryst.* **1975**, *31*, 1735-1739.

6. Analysis of the counter ions in 3c-PF_6

Upon isolation of **3c-PF_6**, a resinous material was obtained. ^{1}H NMR analysis of the freshly isolated material indicated formation of the desired diaryliodonium cation but in the corresponding ^{31}P-NMR spectrum, the typical septet of PF_6^- was not observed. Excerpts of the recorded spectra are depicted in Figure S4. The ^{31}P- and ^{19}F-NMR spectra of freshly isolated product suggest formation of hydrolysis/decomposition products derived from hexafluorophosphate (Figure S4, A and B). Decomposition of PF_6^- upon exposure to air, moisture, and temperature is well documented.[12] Conceivable decomposition products that are reflected by the coupling patterns are $PO_2F_2^-$ and PO_3F^{2-}. The ^{31}P and ^{19}F NMR spectra of aged samples do not show signals for $PO_3F_2^-$ anymore, but merely for PO_3F^{2-} and an additional signal that may be assigned to $H_2PO_4^-$. Furthermore, a doublet in the ^{1}H NMR spectrum (Figure S4E) indicates methyl phosphorofluoridate ($MeOPFO_2^-$) as a possible degradation product ($^3J_{H\text{-}P}$ = 12 Hz). The anionic species proposed above were also validated by HRMS analysis (see Table S2).

Table S2. Results of HRMS analysis of **3c-PF_6^-** showing different hydrolysis products of hexafluorophosphate.

Species	*m*/*z* calc.	*m*/*z* found
$H_2PO_4^-$	96.9696	96.9678
$PO_2F_2^-$	100.9609	100.9618
HPO_3F^-	98.9653	98.9646
$PO_2F(OMe)^-$	112.9809	112.9804

[12] a) Liu, M.; Vatamanu, J.; Chen, X.; Xing, L.; Xu, K.; Li, W. Hydrolysis of $LiPF_6$-Containing Electrolyte at High Voltage. *ACS Energy Lett.* **2021**, *6*, 2096-2102. b) Terborg, L.; Nowak, S.; Passerini, S.; Winter, M.; Karst, U.; Haddad, P. R.; Nesterenko, P. N. Ion chromatographic determination of hydrolysis products of hexafluorophosphate salts in aqueous solution. *Anal. Chim. Acta* **2012**, *714*, 121-126. c) Guillot, S. L.; Peña-Hueso, A.; Usrey, M. L.; Hamers, R. J. Thermal and Hydrolytic Decomposition Mechanisms of Organosilicon Electrolytes with Enhanced Thermal Stability for Lithium-Ion Batteries. J. Electrochem. Soc. 2017, 164, A1907-A1917.

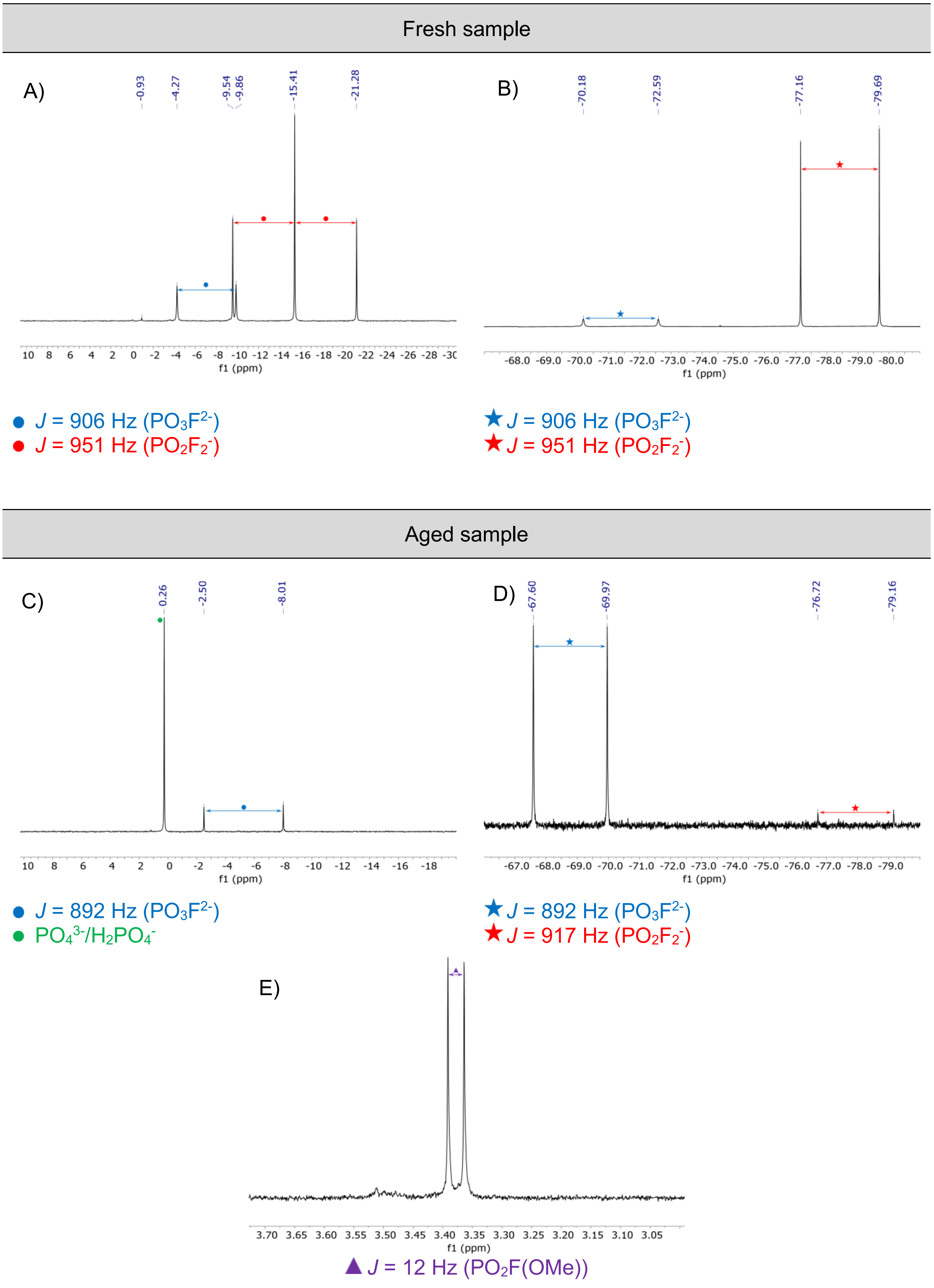

Figure S4. A, B) ^{19}F and ^{31}P NMR spectra of fresh **3c-PF$_6$** sample immediately after workup. C, D, E) ^{19}F, ^{31}P, and ^{1}H NMR spectra of same after storing for eight months.

7. Determination of redox potentials

Linear sweep voltammetry (LSV) was performed in a glass cell of 4 mL volume using an Autolab PGSTAT128N (Metrohm). A 0.1 M solution of $LiClO_4$ in CH_3CN (LiChrosolv® Reag. Ph Eur.) was used as the electrolyte. A Pt wire served as the counter electrode and a Ag/$AgNO_3$ electrode (Ag wire in 0.1 M $AgNO_3$ acetonitrile) [13] as the reference. A Pt microelectrode of 25 μm diameter was used as the working electrode. For analysis of each diaryliodonium salt, a fresh 2 mL solution of blank electrolyte was prepared, and the working electrode polished prior to the measurements. For this purpose, a polishing cloth was used with an aqueous suspension of 0.03 μm SiO_2 particles (Sensolytics).

Analysis of diaryl iodonium salts (**3**): Prior to adding the analyte, the working electrode was cleaned by a series of reduction scans (0 V → −1.5 V vs. Ag/$AgNO_3$) at a scan rate of 0.1 V s^{-1} until the current response stabilized. Then, the appropriate amount of desired diaryliodonium salt **3** (~0.3 mg, corresponding approx. to a concentration of 0.5 mM) was added to the solution and three LSVs were recorded in the same potential range. In most cases, the measured currents significantly decreased from cycle to cycle. This is due to grafting of the electrode surface by reduction to radical products [14] or potentially even blocking electrode surface by products of radical recombination/oligomerization. [15] Therefore, the very first potential sweep after diaryliodonium salt addition was always used for analysis. In the last step, ~1 mg of ferrocene (Acros Organics, 98%) was added to the electrolyte solution and cyclic voltammograms (−0.4 V → +0.2 V → −0.4 V → +0.2 V vs. Ag/$AgNO_3$) were recorded. From these voltammograms, $E_{1/2}$(Fc) of ferrocene oxidation was determined, corresponding to a formal potential of the Fc^+/Fc couple, $E^{\circ\prime}(Fc^+/Fc)$, which was used for referencing of the working electrode potential. The average determined values were $E^{\circ\prime}(Fc^+/Fc) = 0.047 \pm 0.008$ V vs. Ag/$AgNO_3$. All electrode potentials E in the paper are provided with respect to $E^{\circ\prime}(Fc^+/Fc)$ according to

$$E(\text{vs. } E^{\circ\prime}(Fc^+/Fc)) = E(\text{vs. Ag/AgNO}_3) - E^{\circ\prime}(Fc^+/Fc) \qquad (1)$$

After completion of the measurement series, the electrolyte solution was disposed, the cell washed with acetonitrile (3 x 2 mL) and the Pt microelectrode polished as described above. For determination of the diaryliodonium salt reduction potentials, the LSVs recorded in the presence of **3** were corrected for the background currents. In the ideal case, a reduction wave corresponding to **3** would have been obtained on the microelectrode due to hemispherical diffusion field, allowing for determination of the reduction potential as $E_{1/2}$ of the wave. However, this was not the case in the present experiments due to above mentioned blocking of the electrode surface. Moreover, in most of the cases, additional features were apparent in the LSVs at more negative potentials. Therefore, $E_{1/2}$ values (related to reduction potential) of diaryliodonium salts were approximated by the potential $E'_{1/2}(\mathbf{3})$ at which inflexion of the first wave/peak in the LSV occurs. This was determined as potential with the highest 1st derivative of the current $dI\ dE^{-1}$. An example of $E'_{1/2}(\mathbf{3})$ value determination in the case of **3f-ClO₄** is

[13] Pavlishchuk, V. V.; Addison, A. W. Conversion constants for redox potentials measured versus different reference electrodes in acetonitrile solutions at 25 °C. *Inorg. Chim. Acta* **2000**, *298*, 97-102.

[14] a) Steeno, R.; Rodriguez Gonzalez, M. C.; Eyly, S.; Thielemans, W.; Mali, K. S.; De Feyter, S. Covalent Functionalization of Carbon Surfaces: Diaryliodonium versus Aryldiazonium Chemistry. *Chem. Mater.* **2020**, *32*, 5246–5255. b) Matrab, T.; Combellas, C.; Kanoufi, F. Scanning electrochemical microscopy for the direct patterning of a gold surface with organic moieties derived from iodonium salt. *Electrochem. Commun.* **2008**, *10*, 1230-1234.

[15] Buriánek, J. D.; Kvicala, J.; Sekerova, L.; Müller, B. H.; Francke, R.; Bystron, T.; Determination of Diaryliodonium Species by Reverse Iodometric Titration with Ascorbic Acid. *Electroanalysis* **2023**, *35*, e202200376.

presented in Figure S5. A small reduction wave at approx. −0.3 V was assumed to be an impurity, as similar minor waves often appeared in the voltammograms.

Analysis of iodoarenes (**1**): Investigation of compounds **1** was carried out in the same fashion as the analysis of **3**. After cleaning the Pt microelectrode (25 μm diameter) by polishing as described above, a series of oxidation scans (0 V → +2.7 V vs. Ag/$AgNO_3$) was performed until stability of the current response was achieved. For determination of oxidation potentials of iodoarenes, ~2 mM solutions of iodoarene in 0.1 M $LiClO_4$ were used.

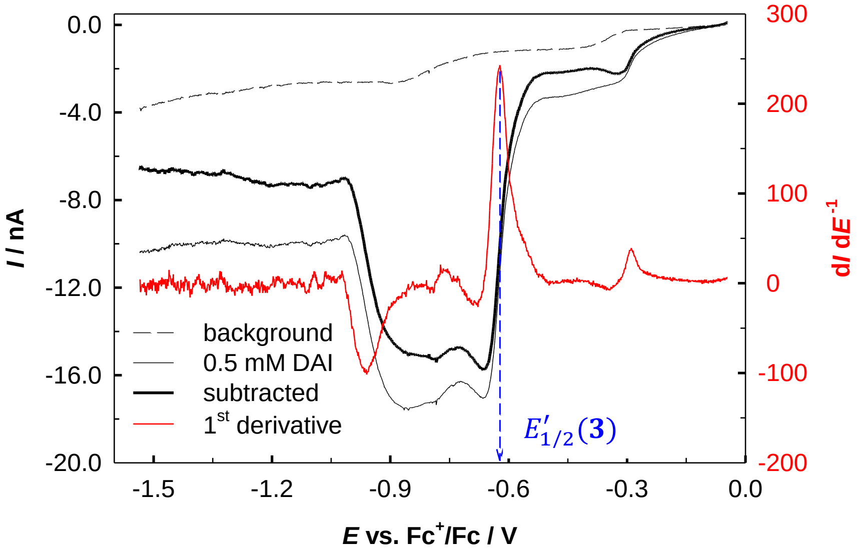

Figure S5. Example for determination of $E_{1/2'}$ value for **3f**-ClO_4. LSVs were recorded at room temperature in a 0.1 M $LiClO_4$ acetonitrile solution in the absence and presence (~0.5 mM) of **3f**-ClO_4 in the electrolyte. Scan rate: 0.1 V s^{-1}; Pt microelectrode as working electrode.
